# Supplementary material for: Early ICD implantation following out-of-hospital cardiac arrest: a retrospective cohort study from the Swedish Registry for Cardiopulmonary Resuscitation
Source: BMJ Open. 2024 Feb 2;14(2):e077137. doi: 10.1136/bmjopen-2023-077137 (PMC10840024; doi:10.1136/bmjopen-2023-077137)
Supplement: Supplementary data [file bmjopen-2023-077137supp004.pdf]

Supplementary Table 1. Baseline characteristics (complete table) in patients who were discharged after out-of-hospital cardiac arrest, in relation to ICD implantation

Variable	No ICD	ICD	P-value	SMD
	2046	959		
Women – n (%)	435 (21.3)	173 (18.1)	0.046	0.081
Age – mean (SD)	63.6 (13.7)	58.5 (15.4)	<0.001	0.347
Born abroad	317 (15.6)	148 (15.5)		
Disposable income, family – median (IQR)	8870.00 [6643.00, 11624.75]	9754.00 [7462.00, 12052.00]	<0.001	0.144
Disposable income, individual – median (IQR)	4454.00 [3062.00, 6171.00]	4951.00 [3353.00, 6296.00]	0.001	0.078
Not employed	978 (52.3)	384 (43.9)		
**Educational level**			0.001	0.203
Pre gymnasium <9 years	344 (18.2)	106 (11.8)		
Post gymnasium 3 years	218 (11.5)	123 (13.7)		
Post gymnasium 3 years or longer	240 (12.7)	124 (13.9)		
Married	1070 (52.7)	540 (56.3)		
**Coexisting conditions prior to OHCA**			Nan	
Hypertension	607 (29.7)	292 (30.4)	0.694	0.017
Ischemic heart disease	280 (13.7)	242 (25.2)	<0.001	0.295
Dyslipidemia	245 (12.0)	171 (17.8)	<0.001	0.165
Stable or unstable angina	213 (10.4)	168 (17.5)	<0.001	0.206
Atrial fibrillation	199 (9.7)	169 (17.6)	<0.001	0.231
Heart failure	181 (8.8)	174 (18.1)	<0.001	0.275
Acute myocardial infarction	178 (8.7)	161 (16.8)	<0.001	0.244
Type 2 diabetes	164 (8.0)	114 (11.9)	0.001	0.130
Alcohol dependency	150 (7.3)	62 (6.5)	0.431	0.034

Sultanian P, *et al. BMJ Open* 2024; 14:e077137. doi: 10.1136/bmjopen-2023-077137

Phobic syndromes	125 (6.1)	48 (5.0)	0.260	0.048
Manic episode	114 (5.6)	34 (3.5)	0.021	0.097
Malignant melanoma	93 (4.5)	34 (3.5)	0.241	0.051
Stroke	79 (3.9)	33 (3.4)	0.643	0.022
Transient ischemic attack (TIA)	63 (3.1)	46 (4.8)	0.025	0.088
Renal failure	73 (3.6)	32 (3.3)	0.830	0.013
Aortic stenosis	54 (2.6)	42 (4.4)	0.016	0.095
Type 1 diabetes	60 (2.9)	31 (3.2)	0.739	0.017
**Medications prior to OHCA**			Nan	
RAAS blockers	548 (26.8)	339 (35.3)	<0.001	0.186
Anticoagulants	405 (19.8)	331 (34.5)	<0.001	0.336
Beta-blockers	434 (21.2)	293 (30.6)	<0.001	0.214
Lipid lowering drugs	349 (17.1)	267 (27.8)	<0.001	0.261
Calcium channel blockers	310 (15.2)	105 (10.9)	0.002	0.125
Diuretics	250 (12.2)	129 (13.5)	0.374	0.037
Anti-acidic peptic drugs	209 (10.2)	87 (9.1)	0.360	0.039
**Location of cardiac arrest**			<0.001	0.477
Home	832 (40.7)	411 (42.9)		
Public place	644 (31.5)	447 (46.6)		
Other places	568 (27.8)	101 (10.5)		
**Prehospital interventions**				
Mechanical compressions	514 (26.2)	257 (27.7)	0.416	0.034
Intubation	295 (14.7)	147 (15.6)	0.591	0.023
Laryngeal airway	426 (33.9)	227 (38.7)	0.050	0.100
Defibrillated, any	1738 (85.8)	867 (91.1)	<0.001	0.165

Defibrillated, times – mean (SD)	2.57 (2.27)	2.99 (2.44)	<0.001	0.179
Adrenaline	719 (36.0)	411 (43.8)	<0.001	0.161
Amiodarone	352 (17.7)	246 (26.4)	<0.001	0.211
**Critical time intervals, minutes – median (IQR)**			Nan	
Arrest to EMS dispatch	2.00 [1.00, 3.00]	2.00 [1.00, 3.00]	0.361	0.065
Arrest to CPR start	0.00 [0.00, 2.00]	1.00 [0.00, 4.00]	<0.001	0.180
Arrest to first defibrillation	6.00 [1.00, 12.00]	10.00 [6.00, 13.00]	<0.001	0.330
Arrest to ambulance arrival	9.00 [6.00, 13.00]	9.00 [7.00, 14.00]	0.021	0.095
Emergency call to ambulance arrival	8.00 [5.00, 13.00]	7.00 [5.00, 11.00]	<0.001	0.245
Arrest to ROSC	11.00 [4.00, 18.00]	13.00 [8.50, 21.00]	<0.001	0.263
**Initial rhythm**			0.001	0.167
VF/pVT	1667 (92.6)	852 (96.2)		
PEA	65 (3.6)	12 (1.4)		
Asystole	69 (3.8)	22 (2.5)		
Circulation on hospital arrival	1932 (95.3)	913 (95.8)	0.614	0.024
Conscious on hospital arrival	1021 (50.9)	345 (36.8)	<0.001	0.288
**Circumstances at time of arrest**			Nan	
Witnessed arrest	1879 (92.7)	866 (91.4)	0.283	0.045
Bystander CPR	1080 (54.4)	743 (78.6)	<0.001	0.531
Telephone CPR	304 (51.3)	233 (57.0)	0.086	0.115
Bystander education			0.643	0.071
Laymen not CPR educated	150 (34.2)	109 (37.3)		
Laymen CPR educated	169 (38.6)	104 (35.6)		

Health care professional	119 (27.2)	79 (27.1)		
Bystander connected AED	167 (15.0)	93 (16.9)	0.354	0.052
Bystander used AED	128 (76.6)	77 (83.7)	0.239	0.177
Witnessed by ambulance	428 (39.3)	50 (10.0)	<0.001	0.724
Ambulance first on scene	1019 (84.6)	430 (77.2)	<0.001	0.190
**Inhospital interventions**			Nan	
PCI	1495 (74.0)	364 (38.9)	<0.001	0.756
CABG	99 (4.9)	34 (3.6)	0.148	0.063

Abbreviations: ROSC = Return of Spontaneous Circulation, PCI = Percutaneous coronary intervention, CABG = Coronary artery bypass grafting, ECMO = Extracorporeal membrane oxygenation, ICD = Implantable cardioverter defibrillator, VF = Ventricular fibrillation, PVT = Pulseless ventricular tachycardia, EMS = Emergency medical system (ambulance), PEA = Pulseless electrical activity, AED = Automated external defibrillator, EU= European Union. SMD = Standardized mean difference, is calculated as the difference in means between the intervention vs non-intervention group, divided of the SD for the covariate. Balance was considered for SMD < 0.1.

1
