# Supplementary material for: Early ICD implantation following out-of-hospital cardiac arrest: a retrospective cohort study from the Swedish Registry for Cardiopulmonary Resuscitation
Source: BMJ Open. 2024 Feb 2;14(2):e077137. doi: 10.1136/bmjopen-2023-077137 (PMC10840024; doi:10.1136/bmjopen-2023-077137)
Supplement: Supplementary data [file bmjopen-2023-077137supp002.pdf]

Supplementary Figure 2. Relative importance using Gradient Boosting

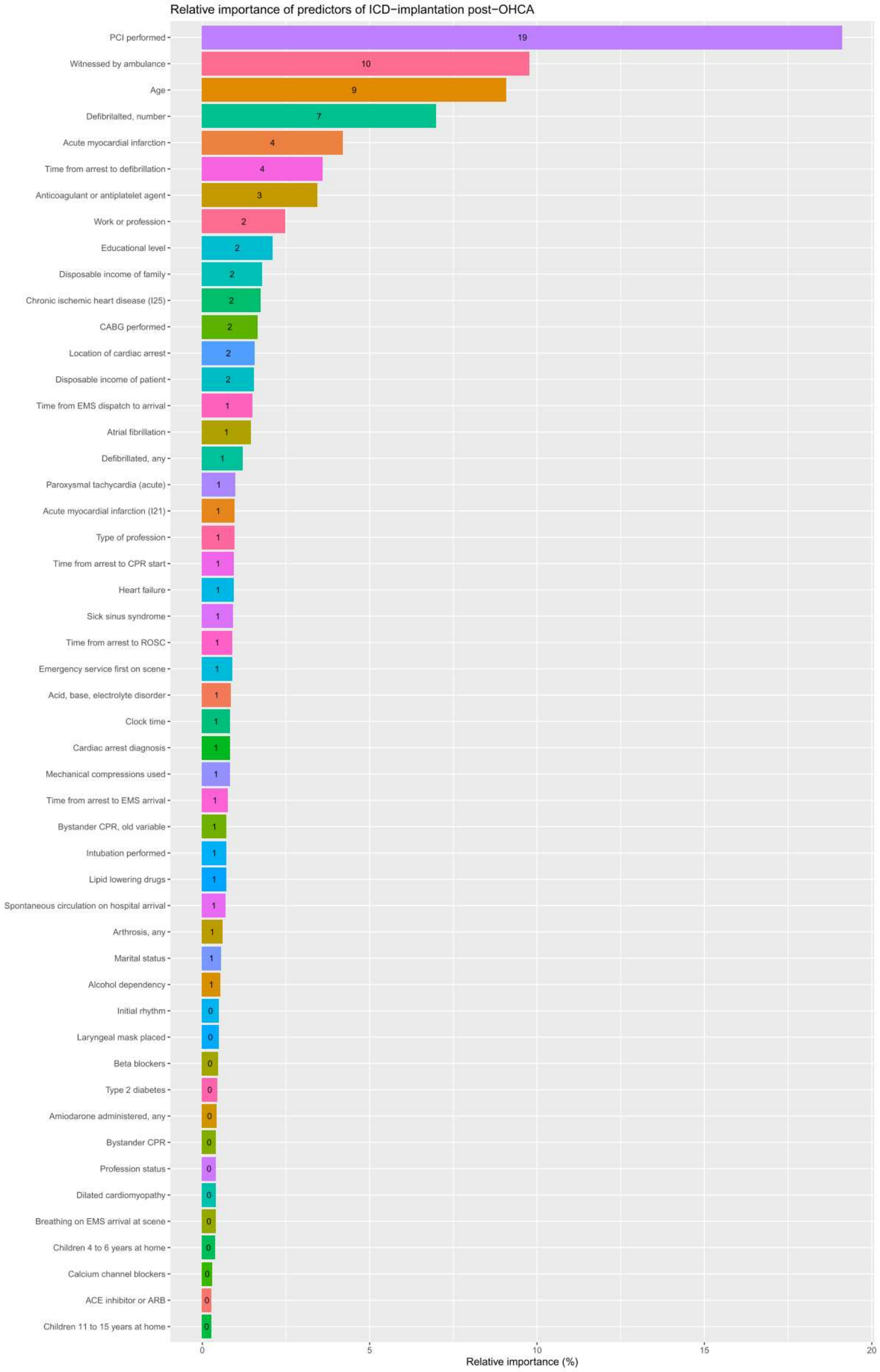

Supplementary Figure 2. Relative importance of top 50 predictors of ICD implantation using gradient boosting. The relative importance is defined as the predictors ability to predict the outcome (i.e. having an ICD at discharge). The direction of the association is not depicted, merely the strength of the association.
