# Supplementary material for: Early ICD implantation following out-of-hospital cardiac arrest: a retrospective cohort study from the Swedish Registry for Cardiopulmonary Resuscitation
Source: BMJ Open. 2024 Feb 2;14(2):e077137. doi: 10.1136/bmjopen-2023-077137 (PMC10840024; doi:10.1136/bmjopen-2023-077137)
Supplement: Supplementary data [file bmjopen-2023-077137supp003.pdf]

Supplementary Figure 3. Cox proportional hazards model for non-balanced covariates

(A)

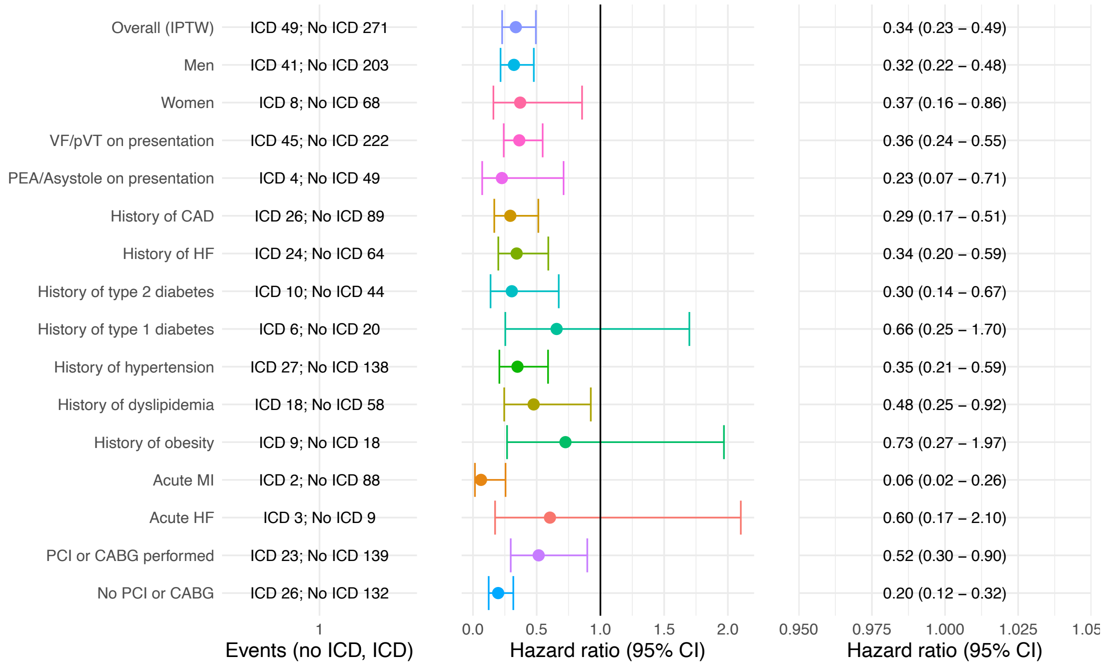

(B)

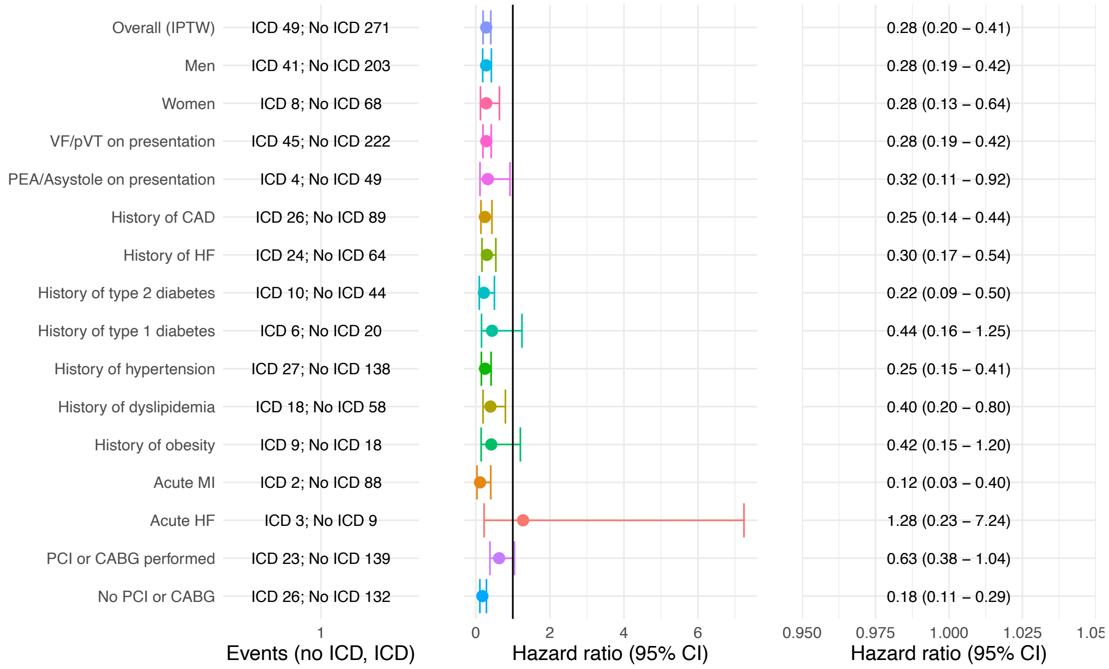

Supplementary figure 3. Cox proportional hazards model for death, recurrent OHCA or IHCA, with adjustments for non-balanced covariates. As evident, adding the non-balanced covariates to the Cox regression did not affect the results. (A) shows results from ATE analysis, and (B) shows results from ATT analysis.
