# Supplementary material for: Early ICD implantation following out-of-hospital cardiac arrest: a retrospective cohort study from the Swedish Registry for Cardiopulmonary Resuscitation
Source: BMJ Open. 2024 Feb 2;14(2):e077137. doi: 10.1136/bmjopen-2023-077137 (PMC10840024; doi:10.1136/bmjopen-2023-077137)
Supplement: Author's manuscript [file bmjopen-2023-077137.draft_revisions.pdf]

BMJ Open is committed to open peer review. As part of this commitment we make the peer review history of every article we publish publicly available.

When an article is published we post the peer reviewers' comments and the authors' responses online. We also post the versions of the paper that were used during peer review. These are the versions that the peer review comments apply to.

The versions of the paper that follow are the versions that were submitted during the peer review process. They are not the versions of record or the final published versions. They should not be cited or distributed as the published version of this manuscript.

BMJ Open is an open access journal and the full, final, typeset and author-corrected version of record of the manuscript is available on our site with no access controls, subscription charges or pay-per-view fees (http://bmjopen.bmj.com).

If you have any questions on BMJ Open's open peer review process please email info.bmjopen@bmj.com

Early ICD implantation and survival after out-of-hospital cardiac arrest

Journal:	*BMJ Open*
Manuscript ID	bmjopen-2023-077137
Article Type:	Original research
Date Submitted by the Author:	26-Jun-2023

Complete List of Authors:	Sultanian, Pedram; University of Gothenburg, Department of Molecular and Clinical Medicine<br>Lundgren, Peter; University of Gothenburg, Department of molecular and clinical medicine; Sahlgrenska University Hospital, Department of Cardiology<br>Rawshani, Aidin; University of Gothenburg, Department of molecular and clinical medicine<br>Möller, Sebastian; Sahlgrenska University Hospital, Department of Cardiology<br>Jafari, Arash Hadi; Halmstad Hospital, Department of Cardiology<br>David, Laura; Sahlgrenska University Hospital, Department of Cardiology<br>Yassinson, Shavan; Kungälvs sjukhus, Department of Cardiology<br>Myredal, Anna; University of Gothenburg, Department of molecular and clinical medicine<br>Rorsman, Cecilia; Varberg Sjukhus, Department of Cardiology<br>Taha, Amar; University of Gothenburg, Department of molecular and clinical medicine; Sahlgrenska University Hospital, Department of Cardiology<br>Ravn-Fischer, Annica; Institution of Molecular and Clinical Medicine, Sahlgrenska University Hospital<br>Martinsson, Andreas; University of Gothenburg, Department of molecular and clinical medicine; Sahlgrenska University Hospital, Department of Cardiology<br>Herlitz, Johan; Faculty of Caring Science, Work Life and Social Welfare, University of Borås<br>Rawshani, Araz; Institute of Medicine, Department of Molecular and Clinical Medicine

Keywords:	Out-of-Hospital Cardiac Arrest, Cardiac Epidemiology < CARDIOLOGY, Cardiopulmonary Resuscitation

SCHOLARONE™ Manuscripts

I, the Submitting Author has the right to grant and does grant on behalf of all authors of the Work (as defined in the below author licence), an exclusive licence and/or a non-exclusive licence for contributions from authors who are: i) UK Crown employees; ii) where BMJ has agreed a CC-BY licence shall apply, and/or iii) in accordance with the terms applicable for US Federal Government officers or employees acting as part of their official duties; on a worldwide, perpetual, irrevocable, royalty-free basis to BMJ Publishing Group Ltd ("BMJ") its licensees and where the relevant Journal is co-owned by BMJ to the co-owners of the Journal, to publish the Work in this journal and any other BMJ products and to exploit all rights, as set out in our licence.

The Submitting Author accepts and understands that any supply made under these terms is made by BMJ to the Submitting Author unless you are acting as an employee on behalf of your employer or a postgraduate student of an affiliated institution which is paying any applicable article publishing charge ("APC") for Open Access articles. Where the Submitting Author wishes to make the Work available on an Open Access basis (and intends to pay the relevant APC), the terms of reuse of such Open Access shall be governed by a Creative Commons licence – details of these licences and which Creative Commons licence will apply to this Work are set out in our licence referred to above.

Other than as permitted in any relevant BMJ Author's Self Archiving Policies, I confirm this Work has not been accepted for publication elsewhere, is not being considered for publication elsewhere and does not duplicate material already published. I confirm all authors consent to publication of this Work and authorise the granting of this licence.

For peer review only

Early ICD implantation and survival after out-of-hospital cardiac arrest

[1]Department of molecular and clinical medicine, Institute of medicine, University of Gothenburg, Gothenburg, Sweden
[2]The Swedish Registry for Cardiopulmonary Resuscitation, Gothenburg, Sweden
[3]Department of Cardiology, Sahlgrenska University Hospital, Gothenburg, Sweden
[4]Department of Cardiology, Halmstad Hospital, Halmstad, Sweden
[5]Department of Cardiology, Varberg Sjukhus, Varberg, Sweden
[6]Department of Cardiology, Kungälvs Sjukhus, Kungälv, Sweden
[7]The Wallenberg Centre for Molecular and Translational Medicine, Gothenburg, Sweden

Pedram Sultanian[1*], Peter Lundgren[1,3], Aidin Rawshani[1], Sebastian Möller[3], Arash Hadi Jafari[4], Laura David[3], Shavan Yassinson[6], Anna Myredal[1], Cecilia Rorsman[5], Amar Taha[1,3], Annica Ravn-Fischer[1,3], Andreas Martinsson[1,3], Johan Herlitz[1,2], Araz Rawshani[1-3]

For peer review only

* Corresponding author. Department of Molecular and Clinical Medicine, University of Gothenburg, Bruna stråket 16, 413 45 Gothenburg, Sweden. Tel: +46 735836248, Email: pedram.sultanian@gu.se

Abstract

Background

It is unclear whether an implantable cardioverter-defibrillator (ICD) is generally beneficial in survivors of out-of-hospital cardiac arrest (OHCA) of cardiac etiology.

Aim

To study the association between ICD implantation and survival in patients with cardiac etiology or initial shockable rhythm in OHCA by using the Swedish Registry for Cardiopulmonary Resuscitation (SRCR).

Methods

All cases in the SRCR during 2010 to 2020 with cardiac etiology or initial shockable rhythm discharged alive were included. We examined the association between ICD and survival using propensity score. Propensity scores (919 candidate predictors before feature selection) were estimated using gradient boosting, Bayesian additive regression trees, neural networks, extreme gradient boosting and logistic regression. Cox regression was used to model survival. The primary outcome was death or recurrent cardiac arrest.

Results

For peer review only

The 90-days event rate was 25% in the ICD group, compared with 52% in those without ICD. Regarding the average treatment effect (ATE) the overall hazard ratio 0.38 (95% CI 0.26-0.56) for ICD vs. no ICD. The overall average treatment effect on those treated (ATT) was 0.31 (95% CI 0.22-0.44). In the 15 subgroup analyses, all point estimates were below 1, with the most pronounced association noted in those with a history of acute myocardial infarction (HR 0.07 [95% CI 0.02-0.32]). All ATE and ATT estimates were aligned.

Conclusions

Mortality in survivors of OHCA occurs early after discharge, and prompt implantation of an ICD is associated with 62% lower probability of death and recurrent cardiac arrest overall.

For peer review only

Introduction

An implantable cardioverter-defibrillator (ICD) is indicated in primary and secondary prevention of sudden cardiac arrest (SCA) from ventricular fibrillation (VF) or ventricular tachycardia (VT). ICDs are considered in patients with persisting significant risk of VT/VF if guideline-directed medical therapy (GDMT) and invasive procedures are insufficient to eliminate future risk of VF/VT. Several randomized trials have compared ICD with GDMT for primary prevention of SCA. With regards to secondary prevention, the Antiarrhythmics Versus Implantable Defibrillators (AVID) demonstrated that an ICD is superior to amiodarone for preventing a recurrent cardiac arrests.[1,2] Guidelines currently recommend that an ICD should be considered in cases without transient or reversible causes of SCA due to VF/VT, hemodynamically unstable VT, sustained VT, provided that life-expectancy is 1 year or longer.

It is still unclear whether SCA and ventricular arrhythmias during an acute myocardial infarction should prompt ICD implantation. It is currently believed that such cases are not at

For peer review only

significant future risk of SCA. However, the underlying substrate (myocardial scar tissue) may persist even if revascularization is successful. A range of factors that are not routinely assessed may influence future risk of ventricular arrhythmias.[1-8] This was presumably demonstrated in the AVID registry, which showed that patients not randomized to ICD, with transient or reversible causes of SCA, had comparable or possibly inferior outcomes compared to those randomized to ICD.[7]

We studied the association between early ICD implantation and survival among patients surviving an out-of-hospital cardiac arrest (OHCA) due to cardiac etiology, or with an initial shockable rhythm.

Methods

All cases enrolled in the Swedish Registry for Cardiopulmonary Resuscitation (SRCR) from Jan 1st 2010 to June 11th 2020 were assessed (n=54956, of whom 54568 had a first OHCA). A total of 3851 with data on ICD implantation were discharged alive. After including only cases with cardiac etiology or initial shockable rhythm, 3005 patients remained in the final study population.

Exposure

Implantation of an ICD during hospitalization was recorded as a binary variable in the registry.

For peer review only

Outcomes

The primary outcomes were the composite of death, recurrent OHCA or IHCA (in-hospital cardiac arrest). Mortality data was retrieved from the Swedish Cause of Death Registry, which has complete ascertainment. Recurrent OHCA and new IHCA events were retrieved using the SRCR, with follow-up ending in June 11th 2020.

Variable selection

A total of 919 candidate predictors were assessed. These included 41 predictors relating to patient phenotype, initial presentation, pre-hospital and in-hospital management (43 predictors), socioeconomic variables (43 predictors), medications (19 agents), previous co-existing conditions and diagnoses received during index hospitalization (814 diagnoses), refer to Table 1 and Supplementary Table 1 and 2 for further details. We removed variables with zero variance, as well as factor variables with >30 levels. With regards to the latter, all variables represented socioeconomic data, which were still adequately represented by remaining variables. The final data set contained 438 candidate predictors of ICD implantation.

Feature selection

We removed variables with zero variance, as well as factor variables with >30 levels.

We used both random forest (RF) and gradient boosting (GBM) to identify the 50 most important predictors of ICD implantation. This was done by computing two separate models with all 438 predictors, using ICD implantation as the dependent variable. The random number generator was not fixed in order to ensure reproducibility across computations. We

For peer review only

121 checked the consistency between random forest and gradient boosting, knowing that these
122 methods yield different variable importance.

123

124 **Propensity score and inverse probability of treatment weighting (IPTW)**

125 We estimated propensity scores and generated balancing weights to estimate the average
126 treatment effect (ATE) and average treatment effect on the treated (ATT). Weights were
127 generated using gradient boosting (GBM), extreme gradient boosting (XGBOOST), Bayesian
128 additive regression trees (BART), neural networks and logistic regression. Each modelling
129 framework was used to model ICD implantation using the 50 top predictors identified by
130 GBM as well as RF, in separate models. Tree-based methods were tuned with regards to tree
131 depth, number of trees and learning rate. Tuning was optimized to minimize covariate
132 standardized mean difference. Standardized mean differences (SMD) below 0.1 were
133 considered non-consequential.[9] The weights were used in a Cox proportional hazards
134 models to calculate hazard ratios for the endpoints (death, recurrent OHCA, IHCA) from
135 discharge to end of follow-up. Subgroup analyses were performed in relation to age, sex,
136 comorbidities and patient characteristics relating to the event.

137

138 Results

139 **Baseline characteristics**

140 A total of 3005 survivors of OHCA were included, of whom 959 had an ICD implanted and
141 2046 were discharged without an ICD. Mean age was 58.5 years and 63.6 years in cases with

For peer review only

For peer review only - http://bmjopen.bmj.com/site/about/guidelines.xhtml

and without ICD implantation, respectively. Women constituted 21% of cases without ICD implanted, compared with 18% of those with an ICD implanted (Table 1).

A history of hypertension was equally prevalent. Previous ischemic heart disease, dyslipidemia, angina pectoris, atrial fibrillation, heart failure, myocardial infarction and diabetes were more prevalent prior to OHCA in cases who received an ICD. Cardiovascular medications were also more common in patients who received an ICD (Table 1).

Pre-hospital defibrillations were performed in 91.1% of cases who received an ICD, compared with 85.8% of those who did not receive an ICD. Adrenaline and amiodarone were more frequently administered in cases who received an ICD. The initial rhythm was shockable in 96.2% of cases who received an ICD, compared with 92.6% of cases who did not. The vast majority (95.8% and 95.3% in those with and without ICD, respectively) in both groups displayed signs of spontaneous circulation at arrival to hospital. PCI was performed more frequently in those who did not receive an ICD (Table 1).

Survival

A total of 320 deaths, 138 recurrent OHCAs and 70 IHCAs occurred after discharge. The most pronounced survival trait was noted for cases who did not receive an ICD, of whom the majority of deaths occurred early after discharge (Figure 1). In men and women, 45% and 58% of those who experienced the composite endpoint did so within 90%s, with the majority doing so within 30 days. In relation to ICD implantation, of those experiencing events, 25% did so in the ICD group, compared with 52% in the group not receiving an ICD.

For peer review only

Modelling results

After removal of predictors with zero variance as well as predictors with >30 factor levels, 438 candidate predictors of ICD implantation remained. Regarding predictors with >30 factor levels, we noted that the information contained in these were available in other predictors that were retained (virtually all conveying information of socio-demographics).

RF and GBM identified (in separate models) 50 predictors with greatest influence in ICD implantation. The predictors not shared by both frameworks were the following: educational level, individual income, time from emergency dispatch to EMS arrival, clock at time of cardiac arrest, use of mechanical compressions, intubation, use of laryngeal intubation, marital status, alcohol misuse, type 2 diabetes, children aged 4 to 6 and 11 to 15 at home, use of calcium channel blockers. Supplementary Figures 1 and 2 shows variable importance plots. Figure 2 presents variable importance plots for each model.

Covariate balance

Covariate balance obtained using GBM and top 50 predictors identified by RF yielded the best covariate balance, as judged by differences in standardized mean difference, coefficient of variation and distribution of weights. Bayesian additive regression trees yielded the second best balance. Figure 2 displays the unadjusted and weight-adjusted covariate balance across all ten models (left panel), along with the RF derived variable importance (right panel). After adjusting for weights, the GBM model had not balanced the following covariates: history of arthrosis, breathing at EMS arrival, OHCA witnessed by ambulance, use of anticoagulants prior to OHCA, place of cardiac arrest and arrhythmias during hospitalization. The latter was the 41st most important predictor among the 50 most

For peer review only - http://bmjopen.bmj.com/site/about/guidelines.xhtml

important, and included diagnoses such as sick sinus syndrome, brady-tachy arrhythmias, premature beats and ventricular fibrillation (ICD-10 code I.49). That variable was not among the 50 most important predictors for the GBM model.

Association between ICD implantation and outcomes

All point estimates, for both ATE and ATT analyses were below 1.0, with the vast majority being significant, favoring ICD implantation (Figure 3).

With regards to ATE, the overall hazard ratio (95% CI) for ICD vs no ICD was 0.38 (0.26-0.56). The point estimate was 0.40 among women, although not statistically significant (8 events occurred in females with ICD). For cases with VF/pVT (pulseless ventricular tachycardia) as initial rhythm HR was 0.42 (0.28-0.63). For cases with PEA/asystole HR was 0.18 (0.06-0.58). HR for those with a history of CAD, heart failure and diabetes were 0.32 (0.20-0.53), 0.36 (0.22-0.61), 0.30 (0.13-0.69), respectively. The lowest point estimates were noted for cases with an acute MI, as well as those who did not undergo PCI or CABG.

With regards to ATT, the hazard ratios for ICD vs no ICD were aligned with those obtained for ATE.

With regards to non-balanced covariate, adding them to the Cox regression did not affect any point estimate (Supplementary Figure 3).

For peer review only

Discussion

The benefit of an implantable cardioverter-defibrillator (ICD) after sudden cardiac arrest (SCA) is still being unraveled. Current clinical guidelines suggests that patients developing ventricular arrythmias due to a cardiac event, in which the underlying condition resolves does not have an increased risk for future SCA, thus not eligible for ICD therapy. However, previous reports have suggested otherwise, showing these individuals have an elevated risk for future malignant arrhythmias.[2,7,8] These ideas are supported by and described in the Antiarrhythmics Versus Implantable Defibrillators (AVID) study.[1] In this study, we approached this issue by examining the average treatment effect (ATE) and the average treatment effect on those treated (ATT) in cases with OHCA of presumed cardiac etiology, or with an initial shockable rhythm. Our results clearly demonstrate that those discharged with an ICD were at substantially lower risk of death or recurrent cardiac arrest, with all hazard ratios around 0.3, suggesting a 70% reduction in the probability of death or recurrent arrest. While these results encourage a broader use of ICDs, one cannot exclude confounding by indication given the observational nature of the data.

We found that the highest incidence rate for death or recurrent cardiac arrest was seen early after discharge (Figure 1). Aligned with these results, a Danish study (concluded during 2007-2011) found that patients with early ICD implantation (during hospitalization) had a higher survival probability post discharge, with hazard ratios similar to ours; hazard ratio for death in the ICD group *vs.* no ICD was 0.44 (95% CI 0.23–0.88).[11] Other studies have presented similar trends in results.[1,2,10,11]

For peer review only

European clinical guidelines recommends ICD implantation for patients with malignant arrhythmias after an acute myocardial infarction with pre-existing left ventricular dysfunction or incomplete revascularization (*class IIb C indication*).[12-16] ICD implantation is recommended regardless of etiology for secondary prevention in patients with dilated cardiomyopathy or hypokinetic non-dilated cardiomyopathy in survivors of OHCA with an initial shockable rhythm, or with hemodynamically significant monomorphic ventricular tachycardia (*class I recommendation*).[16] After a cardiac arrest, patients are followed at an outpatient clinic and if left ventricular ejection fraction is not restored adequately, then an ICD is recommended. However, our study suggests that this initial time period after discharge carries the highest risk for these patients and future guidelines should take this into consideration.[10]

In this study, we also performed 15 subgroup analyses (Figure 3) for both ATE and ATT. All analyses demonstrated similar results, with a strong reduction in the probability of death or recurrent cardiac arrest in individuals receiving an ICD. Notable exceptions to this was the non-significance in the subgroups consisting of women, type 1 diabetes, obesity, acute heart failure, and those undergoing PCI or CABG. With regards to women, although the ATE estimate was not significant, the ATT was clearly significant (HR 0.30 [95% CI 0.13-0.66]). Regarding individuals with type 1 diabetes, obesity, acute heart failure, PCI or CABG performed, it is likely that we were underpowered to assess these subgroups.

Another interesting finding was that PEA/asystole on presentation was associated with a lower risk of outcome in the ICD group. This may be explained by the fact that ambulance response times are increasing which is strongly associated with reductions in the rate of

For peer review only

For peer review only - http://bmjopen.bmj.com/site/about/guidelines.xhtml

cases presenting with shockable rhythms.[17] Hence, a significant proportion of those with asystole may have VF/pVT as their instigating event, but deteriorate to non-shockable rhythms by the time the first ECG is recorded. Moreover, in these cases we do not have data on subsequent (after ROSC) occurrences of ventricular arrhythmias, which would increase their likelihood of receiving an ICD.

Limitations

This study is a registry-based study, which hampers our ability to draw causal inferences. We do not have access to exact timing of ICD implantation. We do not have information on in-hospital ECG monitoring data. Residual confounding and confounding by indication are important caveats of propensity score methods. Confounding by indication in this context implies that the propensity score method may be unable to completely remove confounders related to the decision to implant an ICD (e.g patients having longer expected survival, etc).

Randomized trials are warranted to further unravel the optimal use of ICDs post cardiac arrest. Our study suggests that on a population and individual level, broad use of ICDs are associated with markedly lower risk of death and recurrent cardiac arrest after OHCA, such that ICD therapy was associated with a 70% reduction in the probability of these outcomes.

Funding

Swedish Research Council (2019–02019), Swedish state under the agreement between the Swedish government, and the county councils (ALFGBG-971482), and The Wallenberg Centre for Molecular and Translational Medicine.

For peer review only - http://bmjopen.bmj.com/site/about/guidelines.xhtml

Conflict of interest

None declared

Data availability statement

Data is available upon request, please contact PS, AR or JH for further details.

References

1. Douglas P. Zipes et al. A Comparison of antiarrhythmic-drug therapy with implantable defibrillators in patients resuscitated from near-fatal ventricular arrhythmias. N Eng J Med. 1997;337:1576–1583.

2. Goldenberg, I. *et al.* Long-term benefit of primary prevention with an implantable cardioverter-defibrillator: an extended 8-year follow-up study of the Multicenter Automatic Defibrillator Implantation Trial II. *Circulation* **122**, 1265–1271 (2010).

3. Schmidt, A. *et al.* Infarct Tissue Heterogeneity by Magnetic Resonance Imaging Identifies Enhanced Cardiac Arrhythmia Susceptibility in Patients With Left Ventricular Dysfunction. *Circulation* **115**, 2006–2014 (2007).

4. Tamaki, S. *et al.* Cardiac Iodine-123 Metaiodobenzylguanidine Imaging Predicts Sudden Cardiac Death Independently of Left Ventricular Ejection Fraction in Patients With Chronic Heart Failure and Left Ventricular Systolic Dysfunction. *Journal of the American College of Cardiology* **53**, 426–435 (2009).

5. Yan, A. T. *et al.* Characterization of the Peri-Infarct Zone by Contrast-Enhanced

For peer review only

Cardiac Magnetic Resonance Imaging Is a Powerful Predictor of Post–Myocardial Infarction Mortality. *Circulation* **114**, 32–39 (2006).

6. Myerburg, R. J. & Junttila, M. J. Sudden Cardiac Death Caused by Coronary Heart Disease. *Circulation* **125**, 1043–1052 (2012).

7. Wyse D.George *et al.* Life-threatening ventricular arrhythmias due to transient or correctable causes: high risk for death in follow-up. *Journal of the American College of Cardiology* **38**, 1718–1724 (2001).

8. Viskin, S., Halkin, A. & Olgin, J. E. Treatable causes of sudden death: not really 'treatable' or not really the cause? *J Am Coll Cardiol* **38**, 1725–1727 (2001).

9. Austin, P. C. An Introduction to Propensity Score Methods for Reducing the Effects of Confounding in Observational Studies. *Multivariate Behavioral Research* **46**, 399–424 (2011).

10. Matilde Winther-Jensen et al. Implantable caridioverter defibrillator and survival after out of hospital cardiac arrest due to acute myocardial infarction in Denmark in the years 2001-2012, a nationwide study, European Heart Journal: Acute Cardiovascular Care 2017, Vol 6(2) 144-154

11. Helle Søholm, Early ICD implantation in cardiac arrest survivors with acute coronary syndrome – predictors of implantation, ICD-therapy and long-term survival. Scandinavian Cardiovascular Journal. 2021, VOL. 55, NO. 4, 205-212.

12. Connolly SJ, Gent M, Roberts RS, et al. Canadian Implantable Defibrillator Study (CIDS): a randomized trial of the implantable cardioverter defibrillator against amiodarone. Circulation. 2000;101(11):1297–1302.

For peer review only

13. Kuck KH, Cappato R, Siebels J, et al. Randomized comparison of antiarrhythmic drug therapy with implantable defibrillators in patients resuscitated from cardiac arrest: the Cardiac Arrest Study Hamburg (CASH)). Circulation. 2000;102(7):748–754.

14. Priori SG, Blomstrom-Lundqvist C, Mazzanti A, et al. 2015 ESC Guidelines for the management of patients with ventricular arrhythmias and the prevention of sudden cardiac death: The Task Force for the Management of Patients with Ventricular Arrhythmias and the Prevention of Sudden Cardiac Death of the European Society of Cardiology (ESC). Endorsed by: Association for European Paediatric and Congenital Cardiology (AEPC). Eur Heart J. 2015;36(41):2793–2867.

15. Zipes DP, Camm AJ, Borggrefe M, et al. ACC/AHA/ESC 2006 guidelines for management of patients with ventricular arrhythmias and the prevention of sudden cardiac death: A report of the American College of Cardiology/American Heart Association Task Force and the European Society of Cardiology Committee for Practice Guidelines (Writing Committee to Develop guidelines for management of patients with ventricular arrhythmias and the prevention of sudden cardiac death) developed in collaboration with the European Heart Rhythm Association and the Heart Rhythm Society. Europace. 2006; 8(9):746–837.

16. Task force members of the European Heart Journal, 2022 ESC Guidelines for the management of patients with ventricular arrhythmias and the prevention of sudden cardiac death, European Heart Journal (2022) 43, 3997-4126

17. M Jerkeman, P Sultanian et al. Trends in survival after cardiac arrest: a Swedish nationwide study over 30 years. European Heart Journal, Volume 43, Issue 46, 7 December 2022, Pages 4817–4829

For peer review only

360 **Figures**

361 **Figure 1. Survival curves after discharge among survivors of out-of-hospital cardiac arrest**

For peer review only

For peer review only - http://bmjopen.bmj.com/site/about/guidelines.xhtml

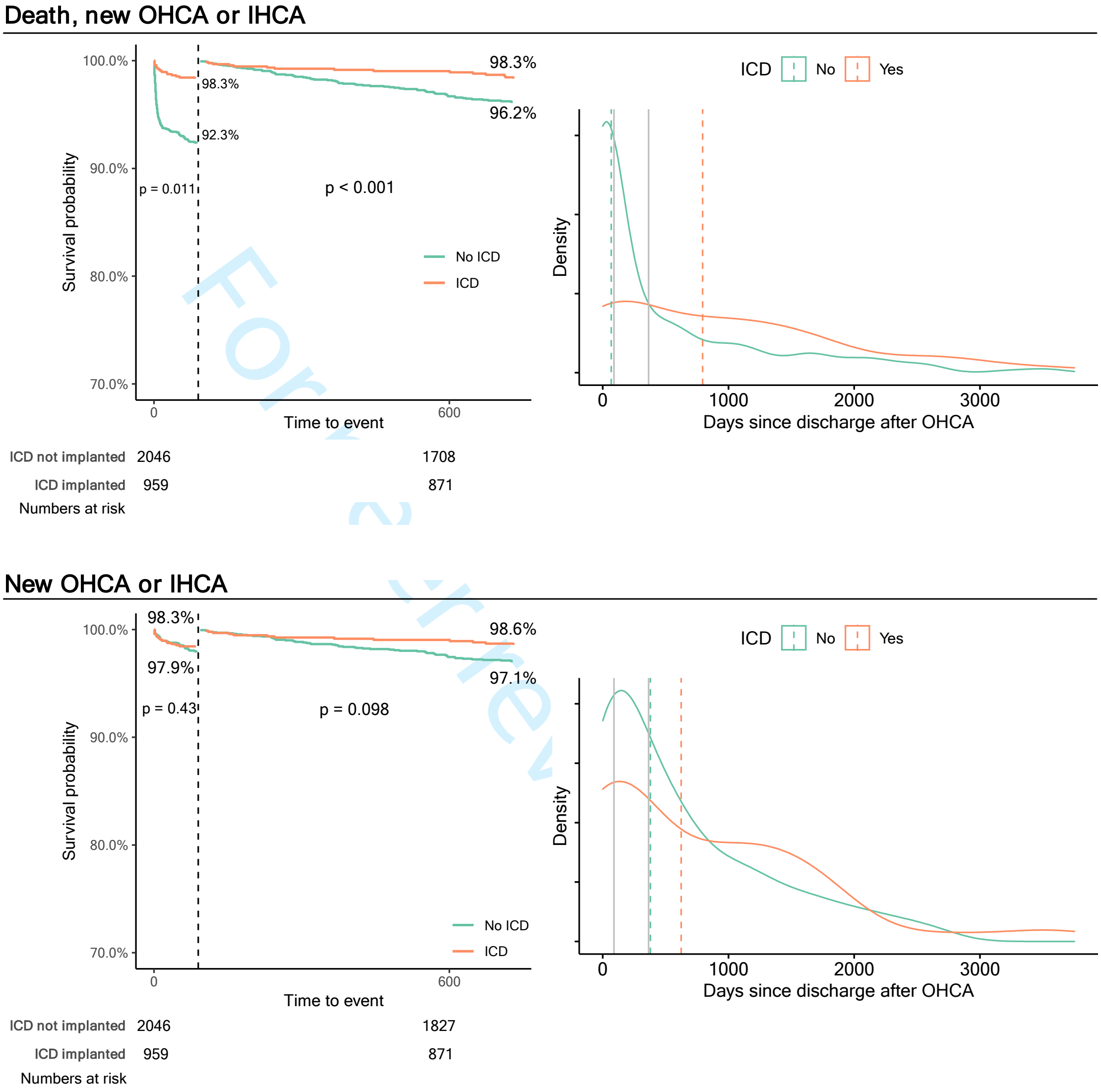

For peer review only - http://bmjopen.bmj.com/site/about/guidelines.xhtml

Figure 2. Covariate balance obtained by 10 different modeling frameworks

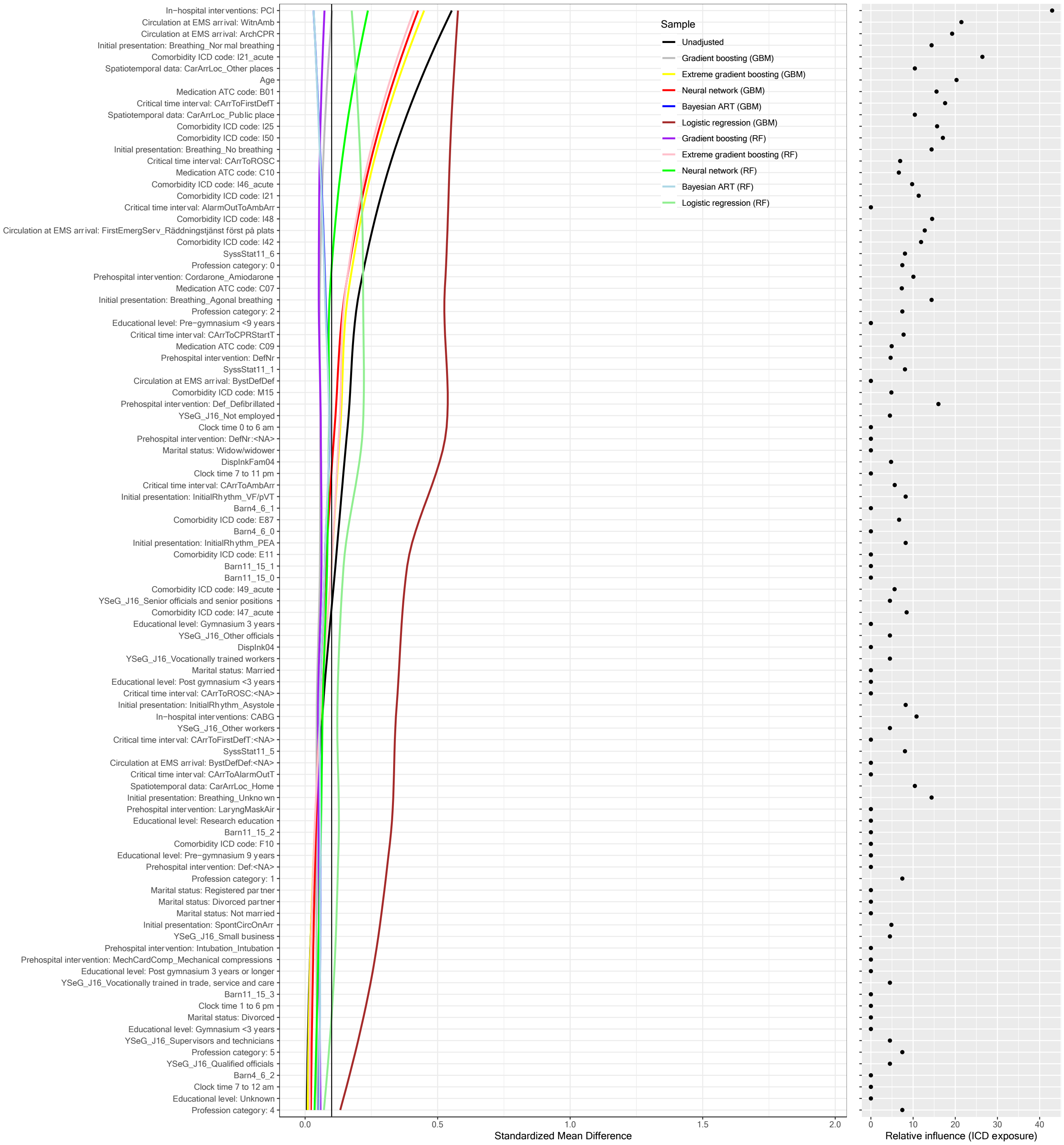

Left panel: covariate balance as assessed by standardized mean differences. Right panel: Relative importance for each variable.

Figure 3. Cox proportional hazards model for death, recurrent OHCA or IHCA

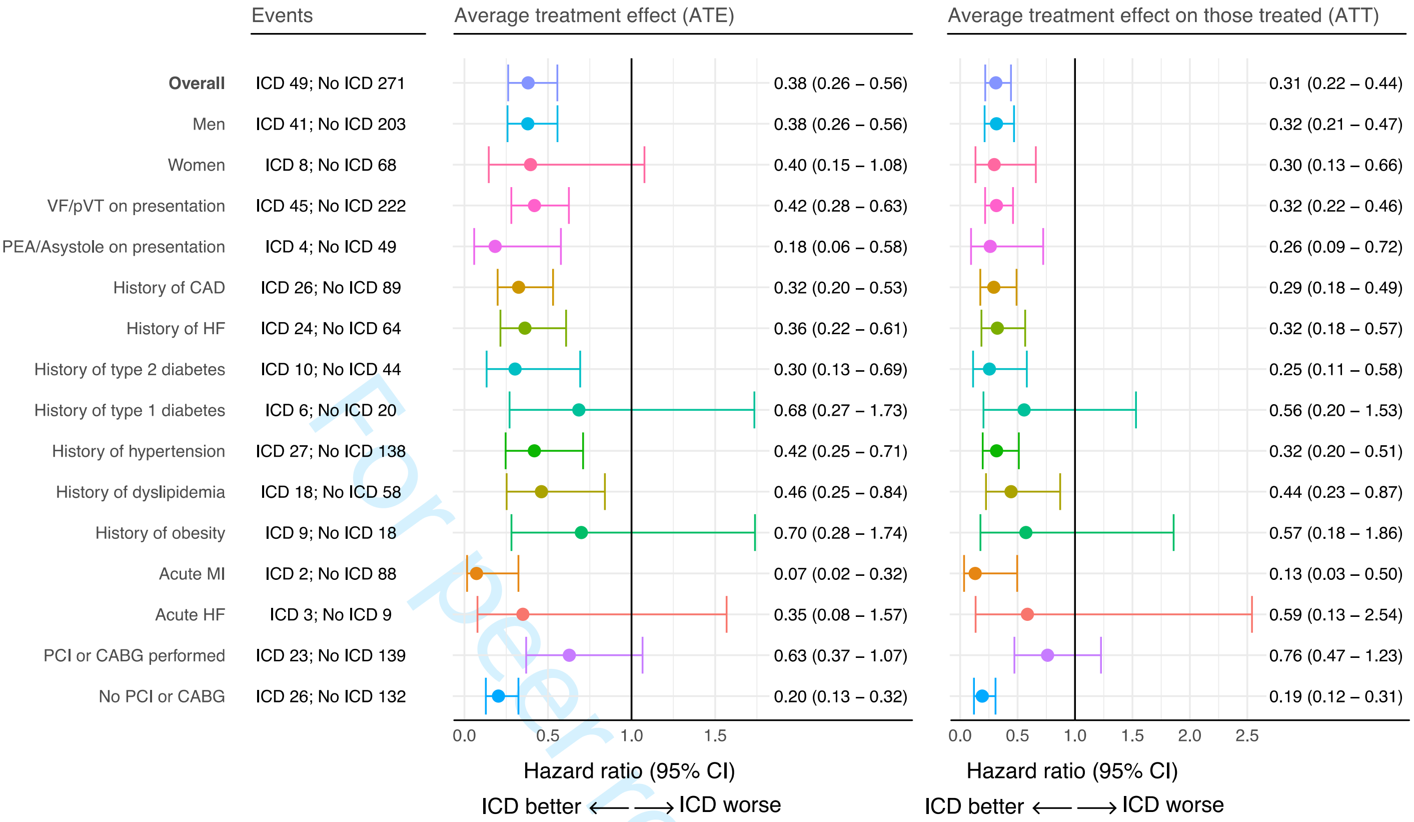

For peer review only

398 **Tables**

Table 1. Baseline characteristics in patients who were discharged after out-of-hospital cardiac arrest, in relation to ICD implantation

Var	No ICD	ICD	P-value	SMD
	2046	959		
Women – n (%)	435 (21.3)	173 (18.1)	0.046	0.081
Age – mean (SD)	63.6 (13.7)	58.5 (15.4)	<0.001	0.347
Born abroad	317 (15.6)	148 (15.5)		
Disposable income, family – median (IQR)	8870.00 [6643.00, 11624.75]	9754.00 [7462.00, 12052.00]	<0.001	0.144
Disposable income, individual – median (IQR)	4454.00 [3062.00, 6171.00]	4951.00 [3353.00, 6296.00]	0.001	0.078
Not employed	978 (52.3)	384 (43.9)		
**Educational level**			0.001	0.203
Pre gymnasium <9 years	344 (18.2)	106 (11.8)		
Post gymnasium 3 years	218 (11.5)	123 (13.7)		
Post gymnasium 3 years or longer	240 (12.7)	124 (13.9)		
Married	1070 (52.7)	540 (56.3)		
**Coexisting conditions prior to OHCA**			Nan	
Hypertension	607 (29.7)	292 (30.4)	0.694	0.017
Ischemic heart disease	280 (13.7)	242 (25.2)	<0.001	0.295
Dyslipidemia	245 (12.0)	171 (17.8)	<0.001	0.165
Stable or unstable angina	213 (10.4)	168 (17.5)	<0.001	0.206
Atrial fibrillation	199 (9.7)	169 (17.6)	<0.001	0.231
Heart failure	181 (8.8)	174 (18.1)	<0.001	0.275
Acute myocardial infarction	178 (8.7)	161 (16.8)	<0.001	0.244

For peer review only - http://bmjopen.bmj.com/site/about/guidelines.xhtml

Type 2 diabetes	164 (8.0)	114 (11.9)	0.001	0.130
Alcohol dependency	150 (7.3)	62 (6.5)	0.431	0.034
Phobic syndromes	125 (6.1)	48 (5.0)	0.260	0.048
Manic episode	114 (5.6)	34 (3.5)	0.021	0.097
Malignant melanoma	93 (4.5)	34 (3.5)	0.241	0.051
Stroke	79 (3.9)	33 (3.4)	0.643	0.022
Transient ischemic attack (TIA)	63 (3.1)	46 (4.8)	0.025	0.088
Renal failure	73 (3.6)	32 (3.3)	0.830	0.013
Aortic stenosis	54 (2.6)	42 (4.4)	0.016	0.095
Type 1 diabetes	60 (2.9)	31 (3.2)	0.739	0.017
**Medications prior to OHCA**			Nan	
RAAS blockers	548 (26.8)	339 (35.3)	<0.001	0.186
Anticoagulants	405 (19.8)	331 (34.5)	<0.001	0.336
Beta-blockers	434 (21.2)	293 (30.6)	<0.001	0.214
Lipid lowering drugs	349 (17.1)	267 (27.8)	<0.001	0.261
Calcium channel blockers	310 (15.2)	105 (10.9)	0.002	0.125
Diuretics	250 (12.2)	129 (13.5)	0.374	0.037
Anti-acidic peptic drugs	209 (10.2)	87 (9.1)	0.360	0.039
**Location of cardiac arrest**			<0.001	0.477
Home	832 (40.7)	411 (42.9)		
Public place	644 (31.5)	447 (46.6)		
Other places	568 (27.8)	101 (10.5)		
**Prehospital interventions**				
Mechanical compressions	514 (26.2)	257 (27.7)	0.416	0.034

For peer review only - http://bmjopen.bmj.com/site/about/guidelines.xhtml

Intubation	295 (14.7)	147 (15.6)	0.591	0.023
Laryngeal airway	426 (33.9)	227 (38.7)	0.050	0.100
Defibrillated, any	1738 (85.8)	867 (91.1)	<0.001	0.165
Defibrillated, times – mean (SD)	2.57 (2.27)	2.99 (2.44)	<0.001	0.179
Adrenaline	719 (36.0)	411 (43.8)	<0.001	0.161
Amiodarone	352 (17.7)	246 (26.4)	<0.001	0.211
**Critical time intervals, minutes – median (IQR)**			Nan	
Arrest to EMS dispatch	2.00 [1.00, 3.00]	2.00 [1.00, 3.00]	0.361	0.065
Arrest to CPR start	0.00 [0.00, 2.00]	1.00 [0.00, 4.00]	<0.001	0.180
Arrest to first defibrillation	6.00 [1.00, 12.00]	10.00 [6.00, 13.00]	<0.001	0.330
Arrest to ambulance arrival	9.00 [6.00, 13.00]	9.00 [7.00, 14.00]	0.021	0.095
Emergency call to ambulance arrival	8.00 [5.00, 13.00]	7.00 [5.00, 11.00]	<0.001	0.245
Arrest to ROSC	11.00 [4.00, 18.00]	13.00 [8.50, 21.00]	<0.001	0.263
**Initial rhythm**			0.001	0.167
VF/pVT	1667 (92.6)	852 (96.2)		
PEA	65 (3.6)	12 (1.4)		
Asystole	69 (3.8)	22 (2.5)		
Circulation on hospital arrival	1932 (95.3)	913 (95.8)	0.614	0.024
Conscious on hospital arrival	1021 (50.9)	345 (36.8)	<0.001	0.288
**Circumstances at time of arrest**			Nan	
Witnessed arrest	1879 (92.7)	866 (91.4)	0.283	0.045
Bystander CPR	1080 (54.4)	743 (78.6)	<0.001	0.531

For peer review only - http://bmjopen.bmj.com/site/about/guidelines.xhtml

Telephone CPR	304 (51.3)	233 (57.0)	0.086	0.115
Bystander education			0.643	0.071
Laymen not CPR educated	150 (34.2)	109 (37.3)		
Laymen CPR educated	169 (38.6)	104 (35.6)		
Health care professional	119 (27.2)	79 (27.1)		
Bystander connected AED	167 (15.0)	93 (16.9)	0.354	0.052
Bystander used AED	128 (76.6)	77 (83.7)	0.239	0.177
Witnessed by ambulance	428 (39.3)	50 (10.0)	<0.001	0.724
Ambulance first on scene	1019 (84.6)	430 (77.2)	<0.001	0.190
**Inhospital interventions**			Nan	
PCI	1495 (74.0)	364 (38.9)	<0.001	0.756
CABG	99 (4.9)	34 (3.6)	0.148	0.063

Abbreviations: rosc = return of spontaneous circulation, pci = percutaneous coronary interventions, cabg = coronary artery bypass grafting, ecmo = extracorporeal membrane oxygenation, icd = implantable cardioverter defibrillator, vf = ventricular fibrillation, pvt = pulseless ventricular tachycardia, ems = emergency medical system (ambulance), pea = pulseless electrical activity, aed = automated external defibrillator, eu=european union.

For peer review only

For peer review only - http://bmjopen.bmj.com/site/about/guidelines.xhtml

Supplementary Figure 1. Relative importance of top 50 predictors of ICD implantation using random forest

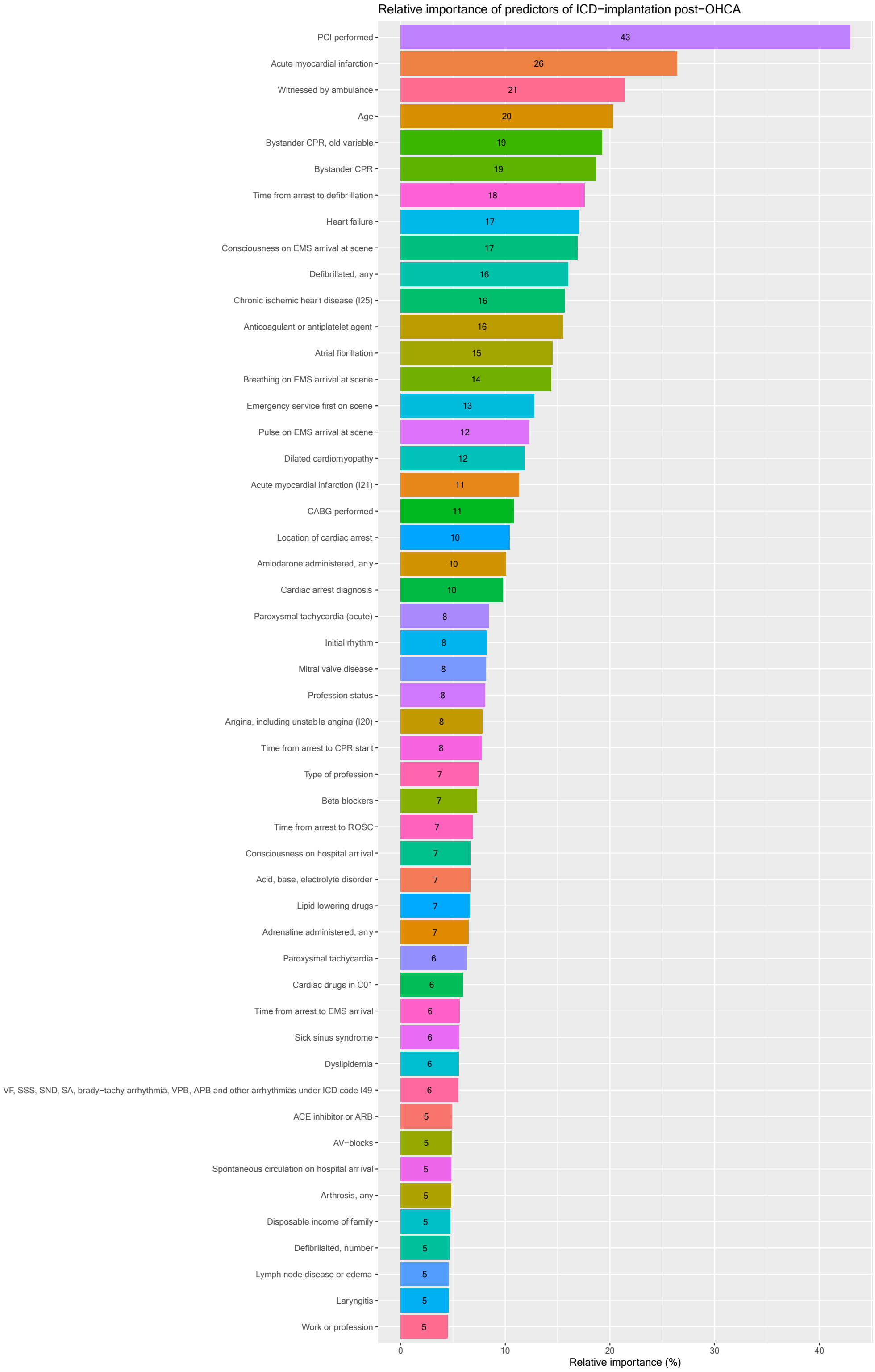

Supplementary Figure 2. Relative importance of top 50 predictors of ICD implantation using gradient boosting

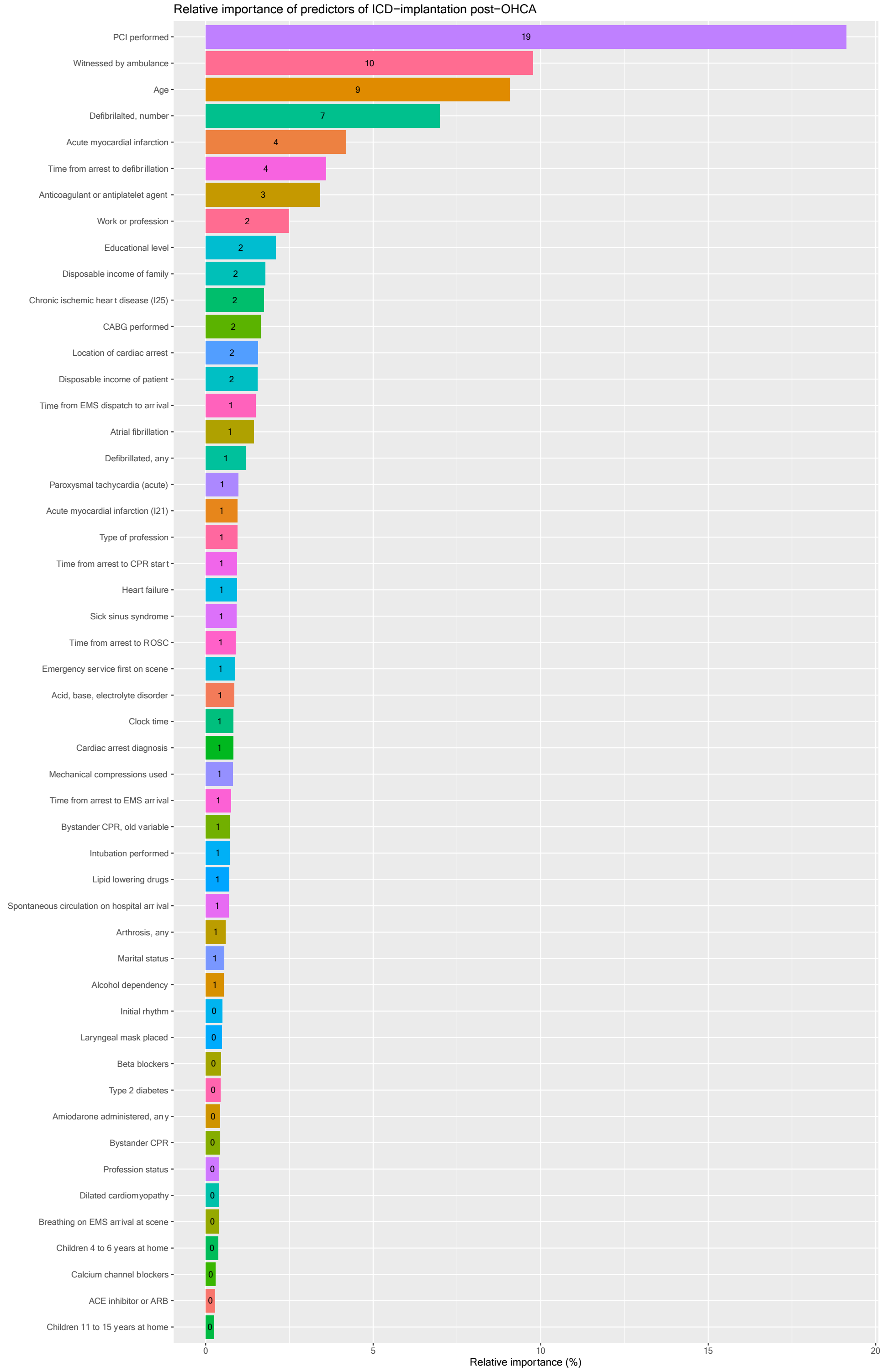

Supplementary Figure 3. Cox proportional hazards model for death, recurrent OHCA or IHCA, with further adjustment for non-balanced covariates

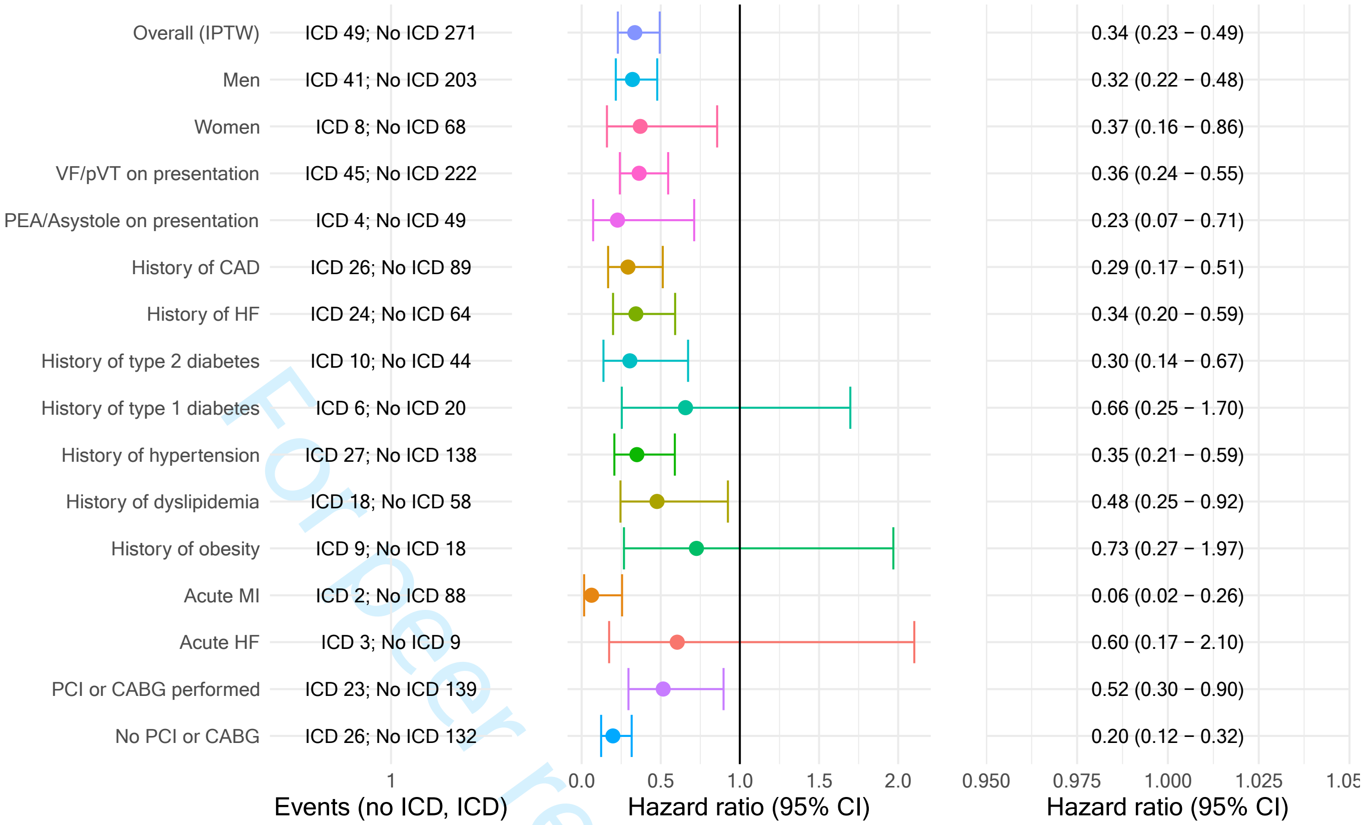

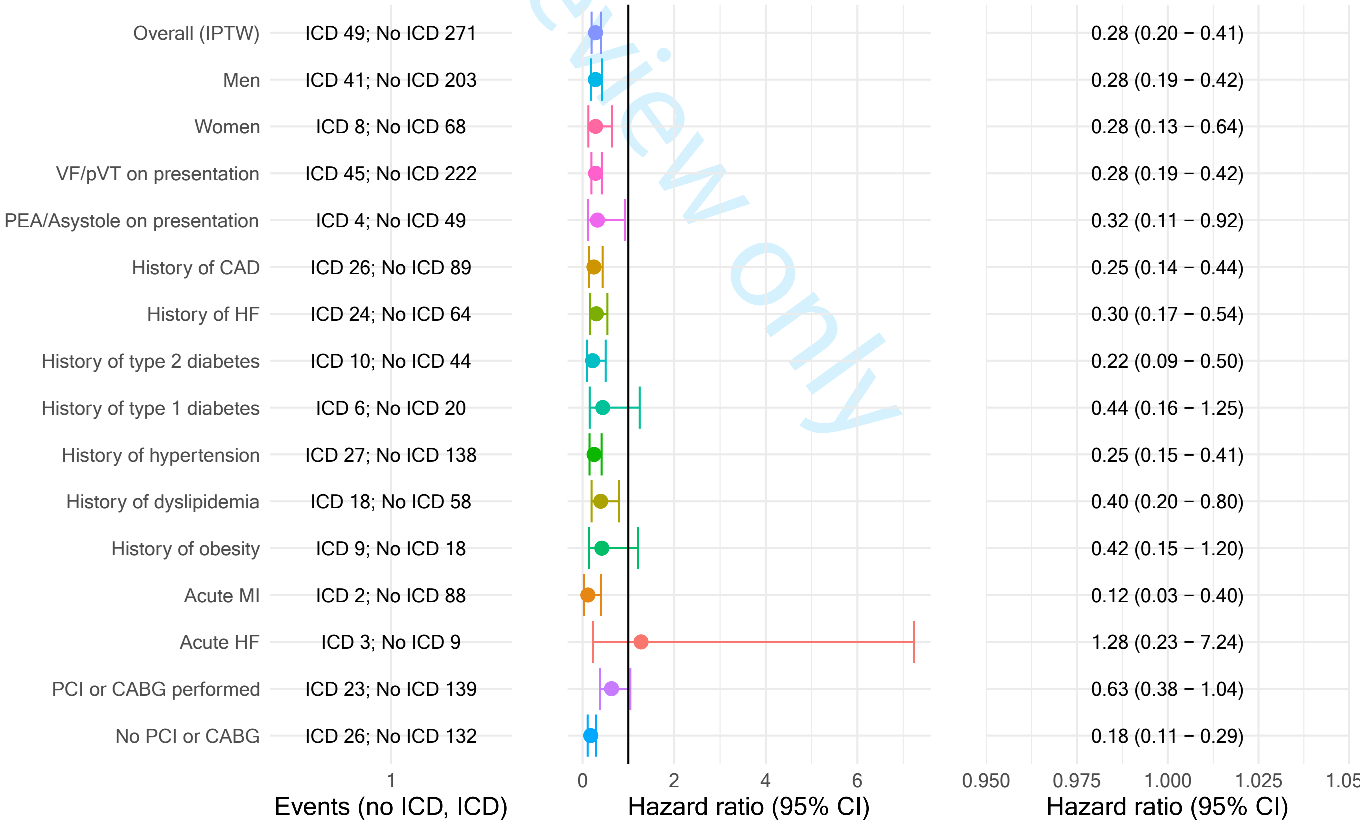

For peer review only

Tables

Table 1. Baseline characteristics in patients who were discharged after out-of-hospital cardiac arrest, in relation to ICD implantation

Var	No ICD	ICD	P-value	SMD
	2046	959		
Women – n (%)	435 (21.3)	173 (18.1)	0.046	0.081
Age – mean (SD)	63.6 (13.7)	58.5 (15.4)	<0.001	0.347
Born abroad	317 (15.6)	148 (15.5)		
Disposable income, family – median (IQR)	8870.00 [6643.00, 11624.75]	9754.00 [7462.00, 12052.00]	<0.001	0.144
Disposable income, individual – median (IQR)	4454.00 [3062.00, 6171.00]	4951.00 [3353.00, 6296.00]	0.001	0.078
Not employed	978 (52.3)	384 (43.9)		
**Educational level**			0.001	0.203
Pre gymnasium <9 years	344 (18.2)	106 (11.8)		
Post gymnasium 3 years	218 (11.5)	123 (13.7)		
Post gymnasium 3 years or longer	240 (12.7)	124 (13.9)		
Married	1070 (52.7)	540 (56.3)		
**Coexisting conditions prior to OHCA**			Nan	
Hypertension	607 (29.7)	292 (30.4)	0.694	0.017
Ischemic heart disease	280 (13.7)	242 (25.2)	<0.001	0.295
Dyslipidemia	245 (12.0)	171 (17.8)	<0.001	0.165
Stable or unstable angina	213 (10.4)	168 (17.5)	<0.001	0.206
Atrial fibrillation	199 (9.7)	169 (17.6)	<0.001	0.231
Heart failure	181 (8.8)	174 (18.1)	<0.001	0.275
Acute myocardial infarction	178 (8.7)	161 (16.8)	<0.001	0.244
Type 2 diabetes	164 (8.0)	114 (11.9)	0.001	0.130
Alcohol dependency	150 (7.3)	62 (6.5)	0.431	0.034
Phobic syndromes	125 (6.1)	48 (5.0)	0.260	0.048

For peer review only

Manic episode	114 (5.6)	34 (3.5)	0.021	0.097
Malignant melanoma	93 (4.5)	34 (3.5)	0.241	0.051
Stroke	79 (3.9)	33 (3.4)	0.643	0.022
Transient ischemic attack (TIA)	63 (3.1)	46 (4.8)	0.025	0.088
Renal failure	73 (3.6)	32 (3.3)	0.830	0.013
Aortic stenosis	54 (2.6)	42 (4.4)	0.016	0.095
Type 1 diabetes	60 (2.9)	31 (3.2)	0.739	0.017
**Medications prior to OHCA**			Nan	
RAAS blockers	548 (26.8)	339 (35.3)	<0.001	0.186
Anticoagulants	405 (19.8)	331 (34.5)	<0.001	0.336
Beta-blockers	434 (21.2)	293 (30.6)	<0.001	0.214
Lipid lowering drugs	349 (17.1)	267 (27.8)	<0.001	0.261
Calcium channel blockers	310 (15.2)	105 (10.9)	0.002	0.125
Diuretics	250 (12.2)	129 (13.5)	0.374	0.037
Anti-acidic peptic drugs	209 (10.2)	87 (9.1)	0.360	0.039
**Location of cardiac arrest**			<0.001	0.477
Home	832 (40.7)	411 (42.9)		
Public place	644 (31.5)	447 (46.6)		
Other places	568 (27.8)	101 (10.5)		
**Prehospital interventions**				
Mechanical compressions	514 (26.2)	257 (27.7)	0.416	0.034
Intubation	295 (14.7)	147 (15.6)	0.591	0.023
Laryngeal airway	426 (33.9)	227 (38.7)	0.050	0.100
Defibrillated, any	1738 (85.8)	867 (91.1)	<0.001	0.165
Defibrillated, times – mean (SD)	2.57 (2.27)	2.99 (2.44)	<0.001	0.179
Adrenaline	719 (36.0)	411 (43.8)	<0.001	0.161
Amiodarone	352 (17.7)	246 (26.4)	<0.001	0.211
**Critical time intervals, minutes – median (IQR)**			Nan	

For peer review only

Arrest to EMS dispatch	2.00 [1.00, 3.00]	2.00 [1.00, 3.00]	0.361	0.065
Arrest to CPR start	0.00 [0.00, 2.00]	1.00 [0.00, 4.00]	<0.001	0.180
Arrest to first defibrillation	6.00 [1.00, 12.00]	10.00 [6.00, 13.00]	<0.001	0.330
Arrest to ambulance arrival	9.00 [6.00, 13.00]	9.00 [7.00, 14.00]	0.021	0.095
Emergency call to ambulance arrival	8.00 [5.00, 13.00]	7.00 [5.00, 11.00]	<0.001	0.245
Arrest to ROSC	11.00 [4.00, 18.00]	13.00 [8.50, 21.00]	<0.001	0.263
**Initial rhythm**			0.001	0.167
VF/pVT	1667 (92.6)	852 (96.2)		
PEA	65 (3.6)	12 (1.4)		
Asystole	69 (3.8)	22 (2.5)		
Circulation on hospital arrival	1932 (95.3)	913 (95.8)	0.614	0.024
Conscious on hospital arrival	1021 (50.9)	345 (36.8)	<0.001	0.288
**Circumstances at time of arrest**			Nan	
Witnessed arrest	1879 (92.7)	866 (91.4)	0.283	0.045
Bystander CPR	1080 (54.4)	743 (78.6)	<0.001	0.531
Telephone CPR	304 (51.3)	233 (57.0)	0.086	0.115
Bystander education			0.643	0.071
Laymen not CPR educated	150 (34.2)	109 (37.3)		
Laymen CPR educated	169 (38.6)	104 (35.6)		
Health care professional	119 (27.2)	79 (27.1)		
Bystander connected AED	167 (15.0)	93 (16.9)	0.354	0.052
Bystander used AED	128 (76.6)	77 (83.7)	0.239	0.177
Witnessed by ambulance	428 (39.3)	50 (10.0)	<0.001	0.724
Ambulance first on scene	1019 (84.6)	430 (77.2)	<0.001	0.190
**Inhospital interventions**			Nan	
PCI	1495 (74.0)	364 (38.9)	<0.001	0.756
CABG	99 (4.9)	34 (3.6)	0.148	0.063

Abbreviations: rosc = return of spontaneous circulation, pci = percutaneous coronary interventions, cabg = coronary artery bypass grafting, ecmo = extracorporeal membrane oxygenation, icd = implantable cardioverter defibrillator, vf = ventricular

For peer review only - http://bmjopen.bmj.com/site/about/guidelines.xhtml

fibrillation, pvt = pulseless ventricular tachycardia, ems = emergency medical system (ambulance), pea = pulseless electrical activity, aed = automated external defibrillator, eu=european union.

For peer review only

Figure 1. Survival curves after discharge among survivors of out-of-hospital cardiac arrest

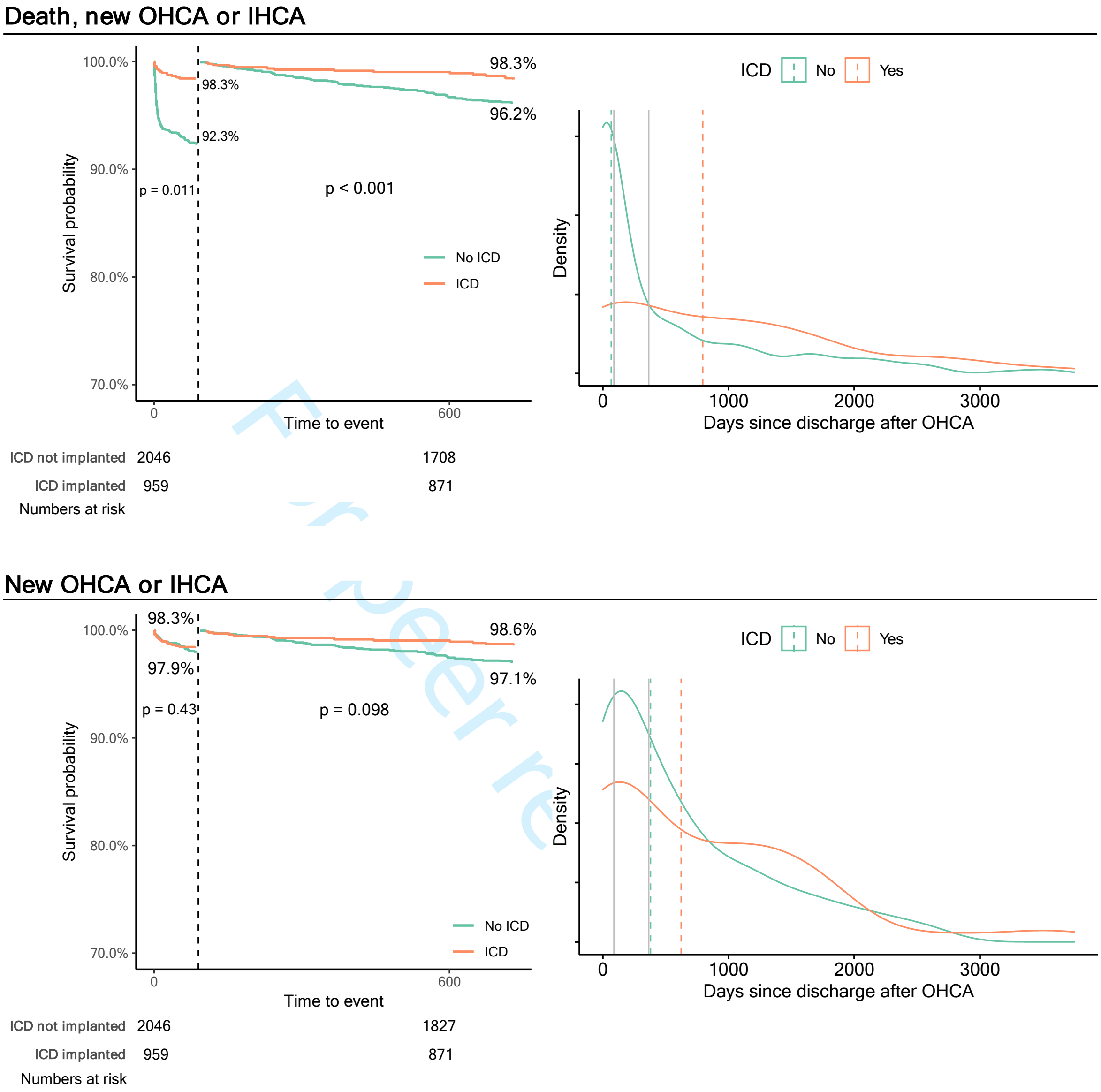

For peer review only

Figure 2. Covariate balance obtained by 10 different modeling frameworks

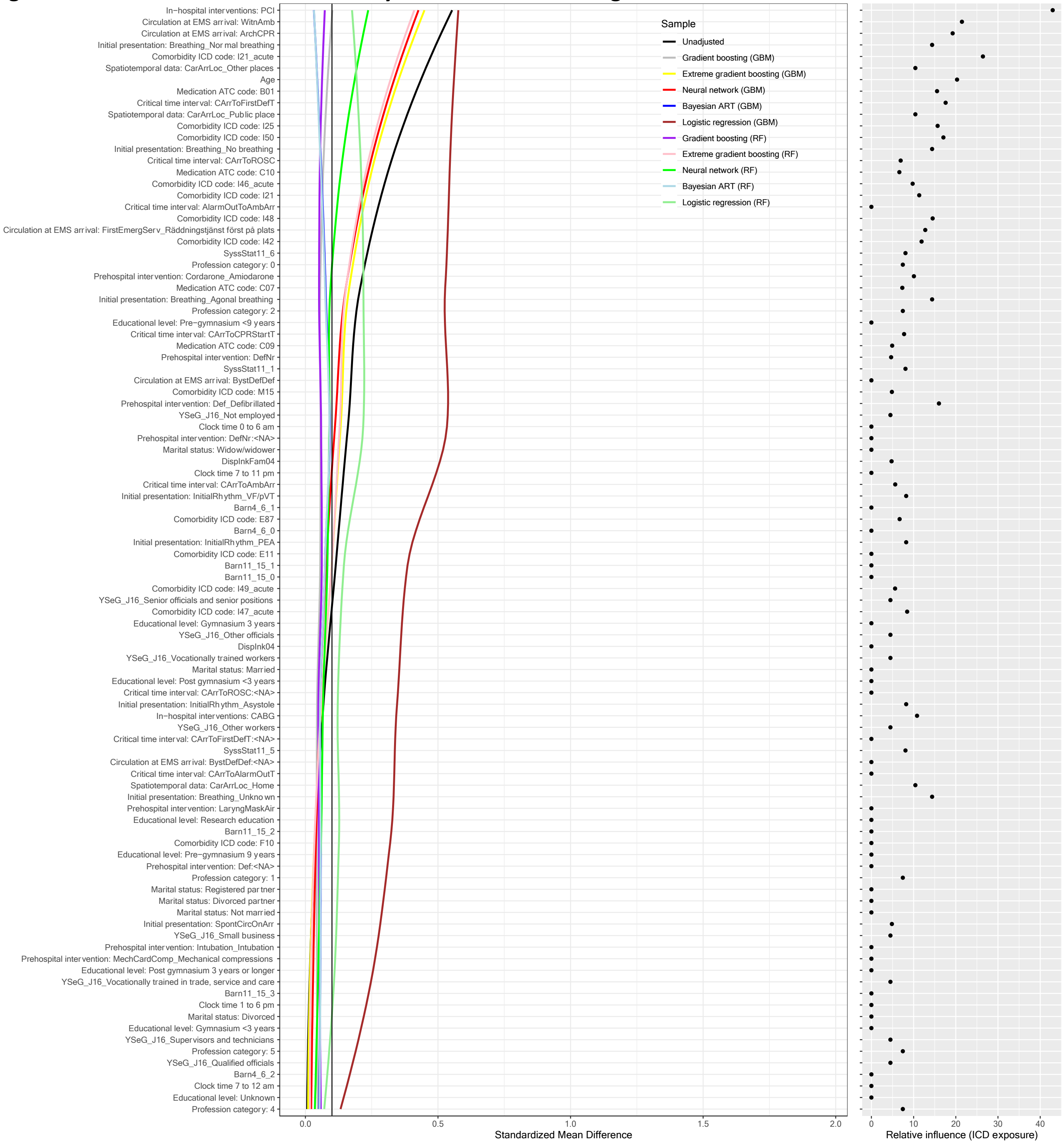

Fig 2. Left panel: covariate balance as assessed by standardized mean differences. Right panel: Relative importance for each variable.

Figure 3. Cox proportional hazards model for death, recurrent OHCA or IHCA

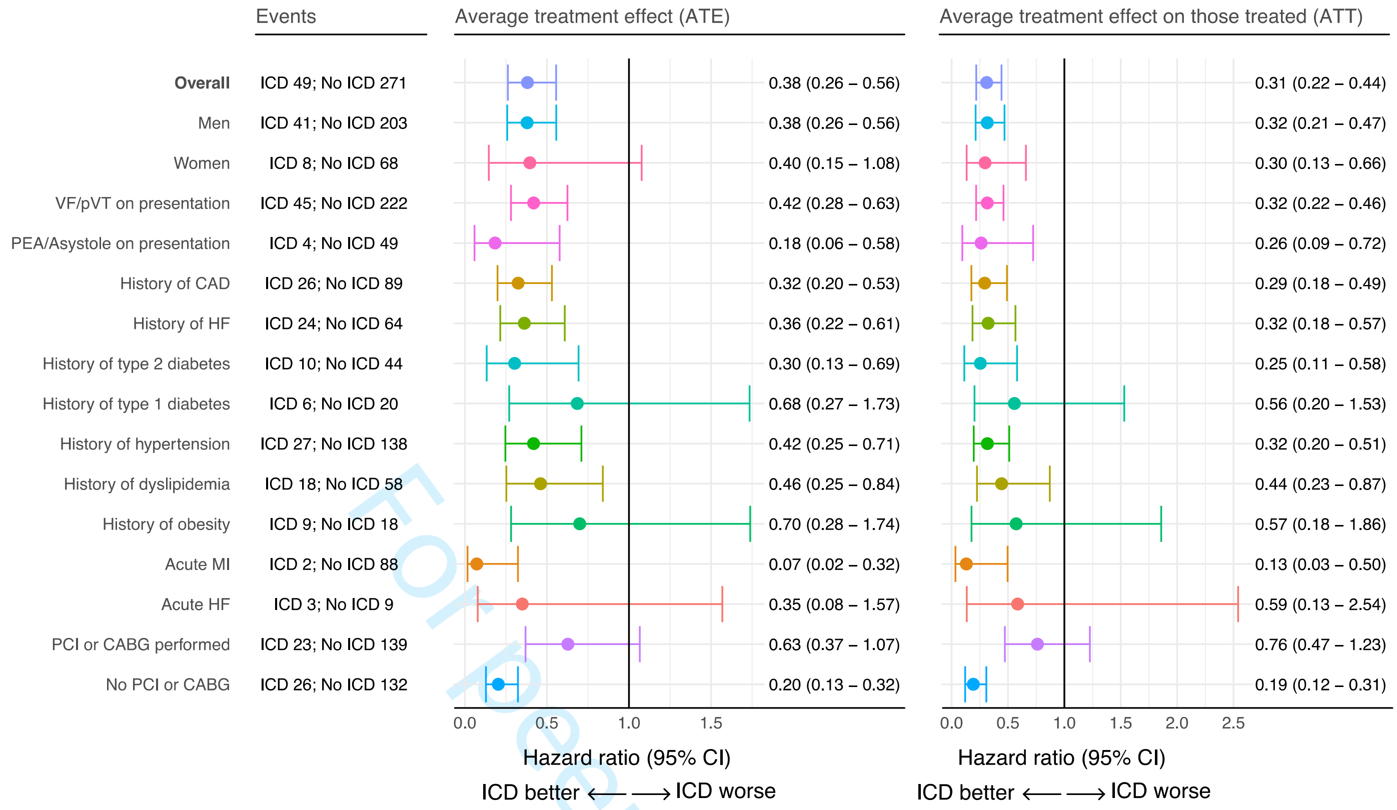

For peer review only

Supplementary files

Supplementary Figure 1. Relative importance of top 50 predictors of ICD implantation using random forest

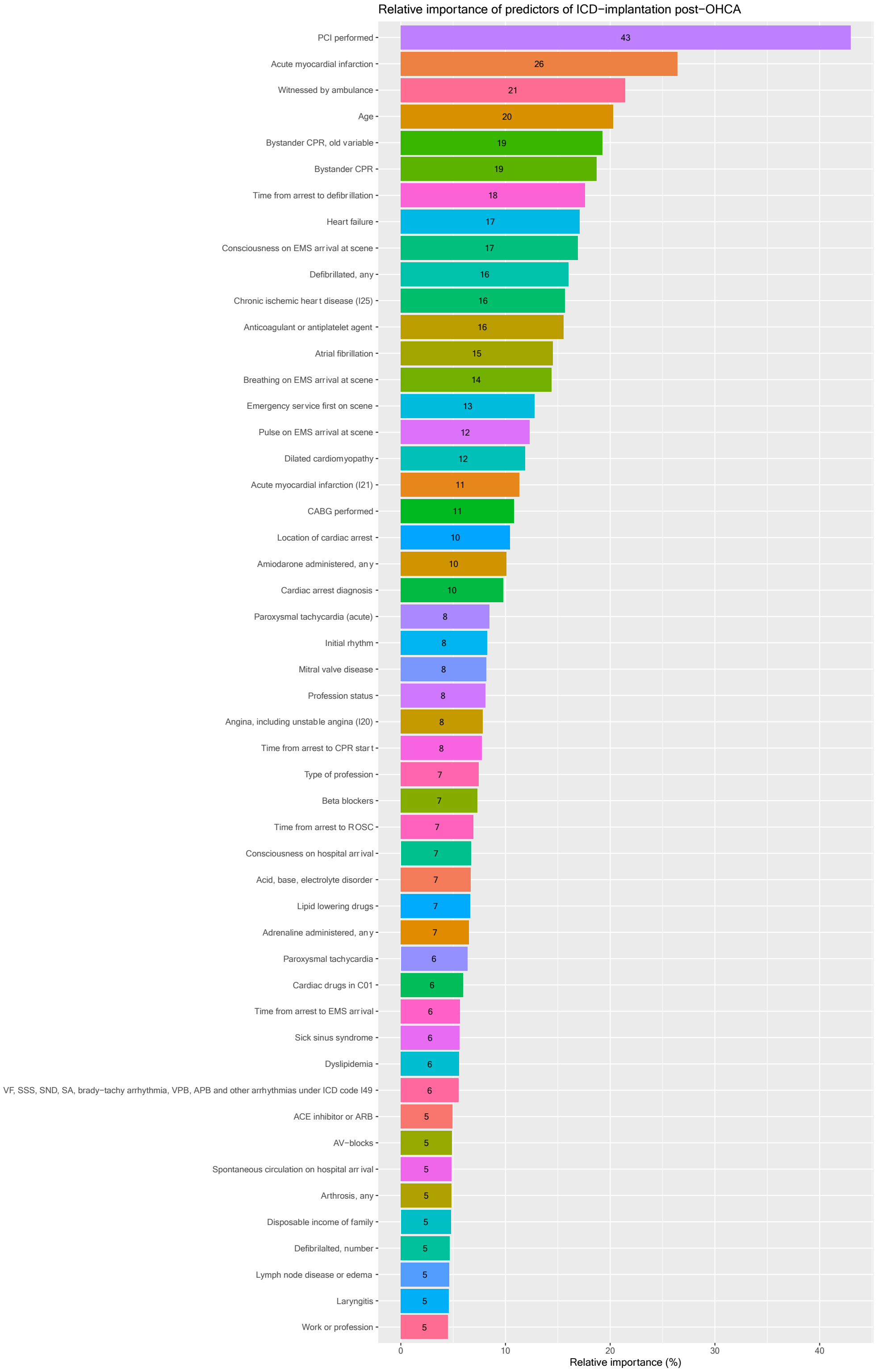

Supplementary Figure 2. Relative importance of top 50 predictors of ICD implantation using gradient boosting

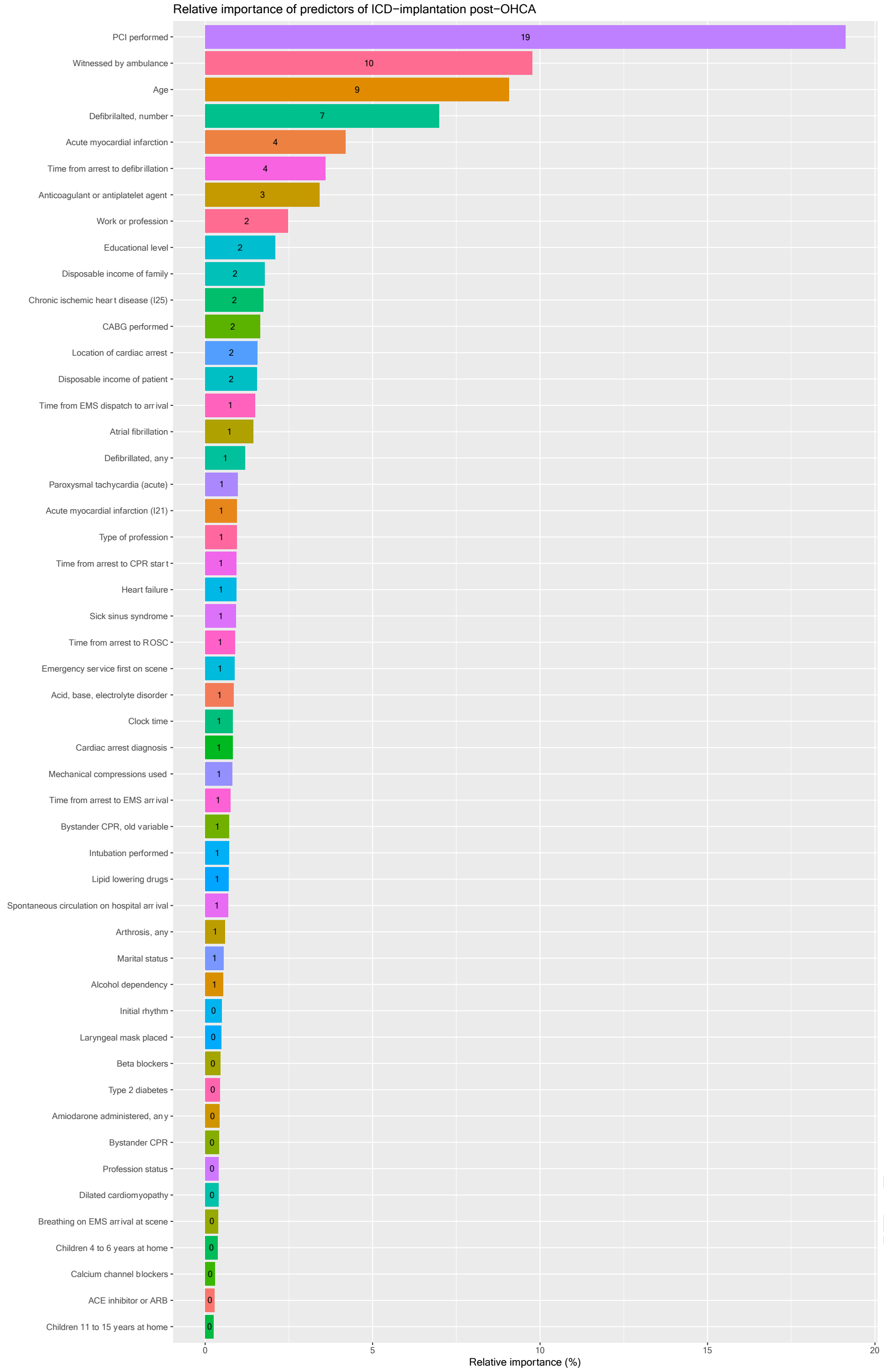

Supplementary Figure 3. Cox proportional hazards model for death, recurrent OHCA or IHCA, with further adjustment for non-balanced covariates

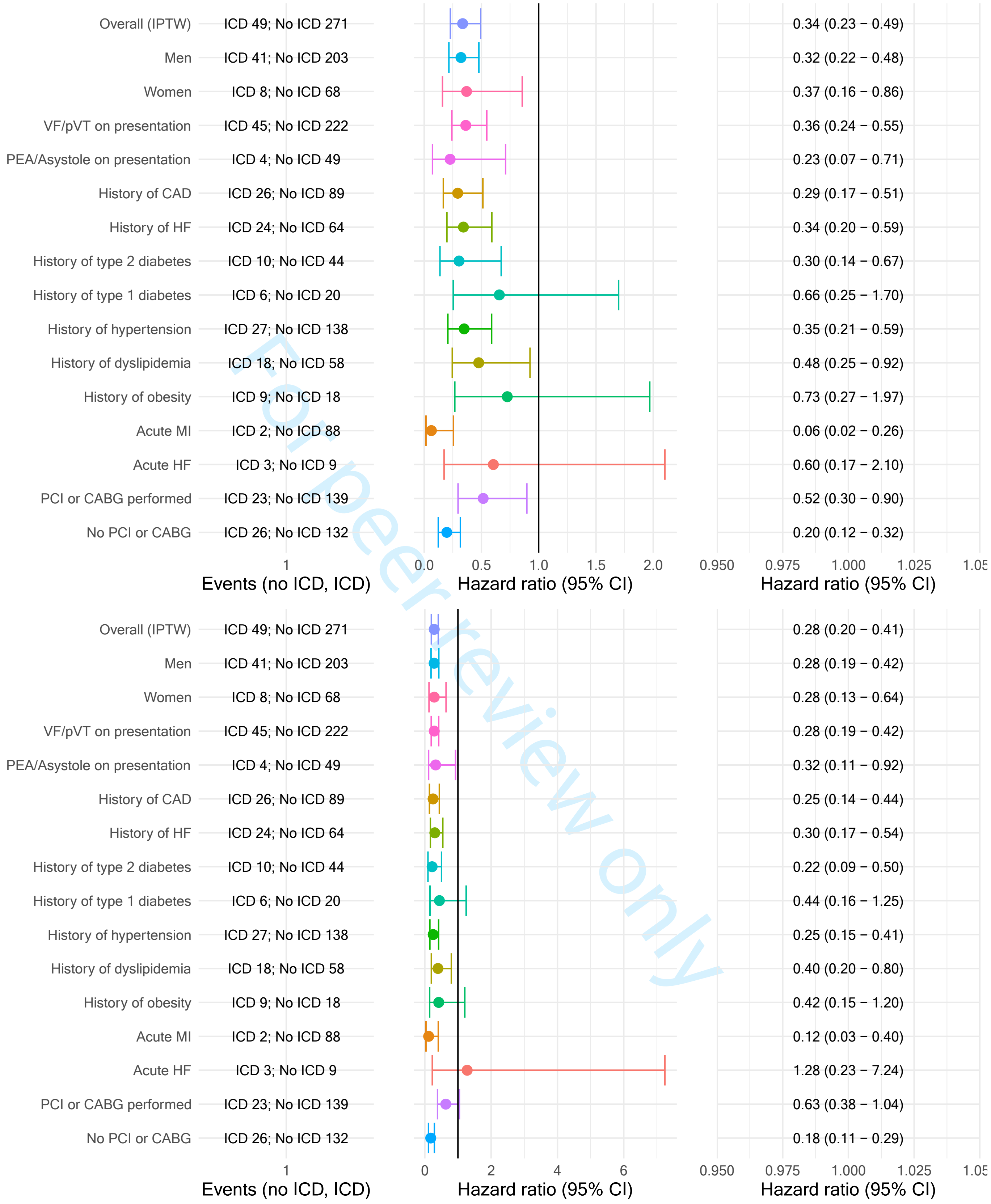

For peer review only

STROBE Statement—checklist of items that should be included in reports of observational studies

	Item No	Recommendation	Page No
**Title and abstract**	1	(*a*) Indicate the study's design with a commonly used term in the title or the abstract	2
		(*b*) Provide in the abstract an informative and balanced summary of what was done and what was found	2,3
**Introduction**			
Background/rationale	2	Explain the scientific background and rationale for the investigation being reported	2,3
Objectives	3	State specific objectives, including any prespecified hypotheses	2
**Methods**			
Study design	4	Present key elements of study design early in the paper	2,3,5-7
Setting	5	Describe the setting, locations, and relevant dates, including periods of recruitment, exposure, follow-up, and data collection	5
Participants	6	(*a*) *Cohort study*—Give the eligibility criteria, and the sources and methods of selection of participants. Describe methods of follow-up<br>*Case-control study*—Give the eligibility criteria, and the sources and methods of case ascertainment and control selection. Give the rationale for the choice of cases and controls<br>*Cross-sectional study*—Give the eligibility criteria, and the sources and methods of selection of participants	5-7
		(*b*) *Cohort study*—For matched studies, give matching criteria and number of exposed and unexposed<br>*Case-control study*—For matched studies, give matching criteria and the number of controls per case	5-7
Variables	7	Clearly define all outcomes, exposures, predictors, potential confounders, and effect modifiers. Give diagnostic criteria, if applicable	5-7, 10-13
Data sources/ measurement	8*	For each variable of interest, give sources of data and details of methods of assessment (measurement). Describe comparability of assessment methods if there is more than one group	5-7
Bias	9	Describe any efforts to address potential sources of bias	10-13
Study size	10	Explain how the study size was arrived at	5
Quantitative variables	11	Explain how quantitative variables were handled in the analyses. If applicable, describe which groupings were chosen and why	5-10
Statistical methods	12	(*a*) Describe all statistical methods, including those used to control for confounding	5-10
		(*b*) Describe any methods used to examine subgroups and interactions	5-10
		(*c*) Explain how missing data were addressed	5
		(*d*) *Cohort study*—If applicable, explain how loss to follow-up was addressed<br>*Case-control study*—If applicable, explain how matching of cases and controls was addressed<br>*Cross-sectional study*—If applicable, describe analytical methods taking account of sampling strategy	
		(*e*) Describe any sensitivity analyses	

Continued on next page

For peer review only - http://bmjopen.bmj.com/site/about/guidelines.xhtml

**Results**			
Participants	13*	(a) Report numbers of individuals at each stage of study—eg numbers potentially eligible, examined for eligibility, confirmed eligible, included in the study, completing follow-up, and analysed	5,7
		(b) Give reasons for non-participation at each stage	
		(c) Consider use of a flow diagram	
Descriptive data	14*	(a) Give characteristics of study participants (eg demographic, clinical, social) and information on exposures and potential confounders	7-10
		(b) Indicate number of participants with missing data for each variable of interest	
		(c) *Cohort study*—Summarise follow-up time (eg, average and total amount)	
Outcome data	15*	*Cohort study*—Report numbers of outcome events or summary measures over time	8
		Case-control study—Report numbers in each exposure category, or summary measures of exposure	
		Cross-sectional study—Report numbers of outcome events or summary measures	
Main results	16	(*a*) Give unadjusted estimates and, if applicable, confounder-adjusted estimates and their precision (eg, 95% confidence interval). Make clear which confounders were adjusted for and why they were included	7-10
		(*b*) Report category boundaries when continuous variables were categorized	
		(*c*) If relevant, consider translating estimates of relative risk into absolute risk for a meaningful time period	
Other analyses	17	Report other analyses done—eg analyses of subgroups and interactions, and sensitivity analyses	12
**Discussion**			
Key results	18	Summarise key results with reference to study objectives	10-13
Limitations	19	Discuss limitations of the study, taking into account sources of potential bias or imprecision. Discuss both direction and magnitude of any potential bias	12,13
Interpretation	20	Give a cautious overall interpretation of results considering objectives, limitations, multiplicity of analyses, results from similar studies, and other relevant evidence	10-13
Generalisability	21	Discuss the generalisability (external validity) of the study results	12,13
**Other information**			
Funding	22	Give the source of funding and the role of the funders for the present study and, if applicable, for the original study on which the present article is based	13

*Give information separately for cases and controls in case-control studies and, if applicable, for exposed and unexposed groups in cohort and cross-sectional studies.

Note: An Explanation and Elaboration article discusses each checklist item and gives methodological background and published examples of transparent reporting. The STROBE checklist is best used in conjunction with this article (freely available on the Web sites of PLoS Medicine at http://www.plosmedicine.org/, Annals of Internal Medicine at http://www.annals.org/, and Epidemiology at http://www.epidem.com/). Information on the STROBE Initiative is available at www.strobe-statement.org.

For peer review only

Early ICD implantation following out-of-hospital cardiac arrest – A retrospective cohort study from the Swedish Registry for Cardiopulmonary Resuscitation

Journal:	*BMJ Open*
Manuscript ID	bmjopen-2023-077137.R1
Article Type:	Original research
Date Submitted by the Author:	19-Oct-2023
Complete List of Authors:	Sultanian, Pedram; University of Gothenburg, Department of Molecular and Clinical Medicine<br>Lundgren, Peter; University of Gothenburg, Department of molecular and clinical medicine; Sahlgrenska University Hospital, Department of Cardiology<br>Rawshani, Aidin; University of Gothenburg, Department of molecular and clinical medicine<br>Möller, Sebastian; Sahlgrenska University Hospital, Department of Cardiology<br>Jafari, Arash Hadi; Halmstad Hospital, Department of Cardiology<br>David, Laura; Sahlgrenska University Hospital, Department of Cardiology<br>Yassinson, Shavan; Kungälvs sjukhus, Department of Cardiology<br>Myredal, Anna; University of Gothenburg, Department of molecular and clinical medicine<br>Rorsman, Cecilia; Varberg Sjukhus, Department of Cardiology<br>Taha, Amar; University of Gothenburg, Department of molecular and clinical medicine; Sahlgrenska University Hospital, Department of Cardiology<br>Ravn-Fischer, Annica; Institution of Molecular and Clinical Medicine, Sahlgrenska University Hospital<br>Martinsson, Andreas; University of Gothenburg, Department of molecular and clinical medicine; Sahlgrenska University Hospital, Department of Cardiology<br>Herlitz, Johan; Faculty of Caring Science, Work Life and Social Welfare, University of Borås<br>Rawshani, Araz; Institute of Medicine, Department of Molecular and Clinical Medicine
**Primary Subject Heading**:	Cardiovascular medicine
Secondary Subject Heading:	Epidemiology
Keywords:	Out-of-Hospital Cardiac Arrest, Cardiac Epidemiology < CARDIOLOGY, Cardiopulmonary Resuscitation

I, the Submitting Author has the right to grant and does grant on behalf of all authors of the Work (as defined in the below author licence), an exclusive licence and/or a non-exclusive licence for contributions from authors who are: i) UK Crown employees; ii) where BMJ has agreed a CC-BY licence shall apply, and/or iii) in accordance with the terms applicable for US Federal Government officers or employees acting as part of their official duties; on a worldwide, perpetual, irrevocable, royalty-free basis to BMJ Publishing Group Ltd ("BMJ") its licensees and where the relevant Journal is co-owned by BMJ to the co-owners of the Journal, to publish the Work in this journal and any other BMJ products and to exploit all rights, as set out in our licence.

The Submitting Author accepts and understands that any supply made under these terms is made by BMJ to the Submitting Author unless you are acting as an employee on behalf of your employer or a postgraduate student of an affiliated institution which is paying any applicable article publishing charge ("APC") for Open Access articles. Where the Submitting Author wishes to make the Work available on an Open Access basis (and intends to pay the relevant APC), the terms of reuse of such Open Access shall be governed by a Creative Commons licence – details of these licences and which Creative Commons licence will apply to this Work are set out in our licence referred to above.

Other than as permitted in any relevant BMJ Author's Self Archiving Policies, I confirm this Work has not been accepted for publication elsewhere, is not being considered for publication elsewhere and does not duplicate material already published. I confirm all authors consent to publication of this Work and authorise the granting of this licence.

For peer review only

Early ICD implantation following out-of-hospital cardiac arrest – A retrospective cohort study from the Swedish Registry for Cardiopulmonary Resuscitation

[1]Department of molecular and clinical medicine, Institute of medicine, University of Gothenburg, Gothenburg, Sweden
[2]The Swedish Registry for Cardiopulmonary Resuscitation, Gothenburg, Sweden
[3]Department of Cardiology, Sahlgrenska University Hospital, Gothenburg, Sweden
[4]Department of Cardiology, Halmstad Hospital, Halmstad, Sweden
[5]Department of Cardiology, Varberg Sjukhus, Varberg, Sweden
[6]Department of Cardiology, Kungälvs Sjukhus, Kungälv, Sweden
[7]The Wallenberg Centre for Molecular and Translational Medicine, Gothenburg, Sweden

Pedram Sultanian[1*], Peter Lundgren[1,3], Aidin Rawshani[1], Sebastian Möller[3], Arash Hadi Jafari[4], Laura David[3], Shavan Yassinson[6], Anna Myredal[1], Cecilia Rorsman[5], Amar Taha[1,3], Annica Ravn-Fischer[1,3], Andreas Martinsson[1,3], Johan Herlitz[1,2], Araz Rawshani[1-3]

* Corresponding author. Department of Molecular and Clinical Medicine, University of Gothenburg, Bruna stråket 16, 413 45 Gothenburg, Sweden. Tel: +46 735836248, Email: pedram_sultanian@hotmail.com

For peer review only

Abstract

Background: It is unclear whether an implantable cardioverter-defibrillator (ICD) is generally beneficial in survivors of out-of-hospital cardiac arrest (OHCA).

Objective: We studied the association between ICD implantation prior to discharge and survival in patients with cardiac etiology or initial shockable rhythm in OHCA.

Design: We conducted a retrospective cohort study in the Swedish Registry for Cardiopulmonary Resuscitation. Treatment associations were estimated using propensity scores. We used gradient boosting, Bayesian additive regression trees, neural networks, extreme gradient boosting and logistic regression to generate multiple propensity scores. We selected the model yielding maximum covariate balance to obtain weights, which were used in a Cox regression to calculate hazard ratios (HR) for death or recurrent cardiac arrest.

Participants: All cases discharged alive during 2010 to 2020 with a cardiac etiology or initial shockable rhythm were included. A total of 959 individuals were discharged with an ICD, and 2,046 were discharged without one.

Results: Among those experiencing events, 25% did so within 90 days in the ICD group, compared with 52% in the other group. All HRs favored ICD implantation. The overall HR (95% CI) for ICD vs no ICD was 0.38 (0.26-0.56). The HR was 0.42 (0.28-0.63) in cases with initial shockable rhythm; 0.18 (0.06-0.58) in non-shockable rhythm; 0.32 (0.20-0.53) in cases with a history of coronary artery disease; 0.36 (0.22-0.61) in heart failure and 0.30 (0.13-0.69) in those diabetes. Similar associations were noted in all subgroups.

Conclusion: Among survivors of OHCA, those discharged with an ICD had approximately 60% lower risk of death or recurrent cardiac arrest. A randomized trial is warranted to study this further.

For peer review only

For peer review only - http://bmjopen.bmj.com/site/about/guidelines.xhtml

Strengths and limitations of this study

- **Large and comprehensive dataset**: The study included all cases enrolled in a nationwide registry, with very high coverage, including cases over a long period (2010-2020).
- **State-of-the-art data analysis**: We used a comprehensive data driven machine learning approach to generate propensity scores, and based our estimates on the score yielding maximum covariate balance.
- **Retrospective cohort study, the nature of data**: This study is a retrospective cohort study which limits causal inferences.
- **Timing of ICD implantation**: ICD at discharge was defined as having an ICD at hospital discharge, without specifying when the ICD had been implanted, such that the results should be interpreted as the association between having an ICD at discharge and survival.

For peer review only

Introduction

An implantable cardioverter-defibrillator (ICD) is indicated in primary and secondary prevention of sudden cardiac arrest (SCA) from ventricular fibrillation (VF) or ventricular tachycardia (VT). ICDs are considered in patients with persisting significant risk of VT/VF if guideline-directed medical therapy (GDMT) and invasive procedures are insufficient to eliminate future risk of VF/VT. Several randomized trials have compared ICD with GDMT for primary prevention of SCA. With regards to secondary prevention, the Antiarrhythmics Versus Implantable Defibrillators (AVID) demonstrated that an ICD is superior to amiodarone for preventing a recurrent cardiac arrest [1,2]. Guidelines currently recommend that an ICD should be considered in cases without transient or reversible causes of SCA due to VF/VT, hemodynamically unstable VT, sustained VT, provided that life-expectancy is 1 year or longer.

It is still unclear whether SCA and ventricular arrhythmias during an acute myocardial infarction should prompt ICD implantation. It is currently believed that such cases are not at significant future risk of SCA. However, the underlying substrate (myocardial scar tissue) may persist even if revascularization is successful. A range of factors that are not routinely assessed may influence future risk of ventricular arrhythmias [1-8]. This was presumably demonstrated in the AVID registry, which showed that patients not randomized to ICD, with transient or reversible causes of SCA, had comparable or possibly inferior outcomes compared to those randomized to ICD [7].

The aim of this study was to study the association between having an ICD at discharge and survival among patients surviving an out-of-hospital cardiac arrest (OHCA) due to cardiac etiology or with an initial shockable rhythm.

Methods

All cases enrolled in the Swedish Registry for Cardiopulmonary Resuscitation (SRCR) from Jan 1st 2010 to June 11th 2020 were assessed. This included a total of 54956 cases of OHCA, of whom 54568 were first events. We excluded all recurring events (n=388), such that only the first OHCA was assessed for each individual. A total of 10836 cases had information on ICD,

For peer review only - http://bmjopen.bmj.com/site/about/guidelines.xhtml

of whom 3851 were discharged alive. Among these 3851 patients we included those with cardiac etiology or initial shockable rhythm, resulting in a final study population of 3005 patients.

Exposure

Implantation of an ICD during hospitalization was recorded as a binary variable in the registry.

Outcomes

The primary outcomes were the composite of death, recurrent OHCA or IHCA (in-hospital cardiac arrest). Mortality data was retrieved from the Swedish Cause of Death Registry, which has complete ascertainment. Recurrent OHCA and new IHCA events were retrieved using the SRCR, with follow-up ending in June 11th 2020.

Initial variable selection

A total of 919 candidate predictors were available to create propensity scores for ICD implantation. These predictors included 41 predictors relating to patient phenotype, initial presentation, pre-hospital and in-hospital management (43 predictors), socioeconomic variables (43 predictors), medications (19 agents), previous co-existing conditions and diagnoses received during index hospitalization (814 diagnoses), refer to Supplementary Table 1 and Supplementary Figure 1 and 2 for further details. We removed variables with zero variance, as well as factor variables with >30 levels. With regards to the latter, all variables represented socioeconomic data, which were still adequately represented by remaining variables. The final data set contained 438 candidate predictors of ICD implantation. These predictors were used in several models (see below) to predict having an ICD at discharge (i.e. the propensity score).

Covariate balance

Covariate balance obtained using GBM and top 50 predictors identified by RF yielded the best covariate balance, as judged by differences in standardized mean difference, coefficient of variation and distribution of weights. Bayesian additive regression trees yielded the second best balance. Figure 1 displays the unadjusted and weight-adjusted covariate balance across all ten models (left panel), along with the RF derived variable importance

For peer review only - http://bmjopen.bmj.com/site/about/guidelines.xhtml

For peer review only

(right panel). After adjusting for weights, the GBM model had not balanced the following covariates: history of arthrosis, breathing at EMS arrival, OHCA witnessed by ambulance, use of anticoagulants prior to OHCA, place of cardiac arrest and arrhythmias during hospitalization. The latter was the 41st most important predictor, and included diagnoses such as sick sinus syndrome, brady-tachy arrhythmias, premature beats and ventricular fibrillation (ICD-10 code I.49). That variable was not among the 50 most important predictors for the GBM model.

Final feature selection

We used both random forest (RF) and gradient boosting (GBM) to identify the 50 most important predictors of ICD implantation. This was done by computing two separate models with all 438 predictors, using ICD implantation as the dependent variable. The random number generator was not fixed, in order to ensure reproducibility across computations. We checked the consistency between random forest and gradient boosting, knowing that these methods yield different variable importance.

Propensity score and inverse probability of treatment weighting (IPTW)

We estimated propensity scores and generated balancing weights to estimate the average treatment effect (ATE) and average treatment effect on the treated (ATT). ATE measures the average treatment effect in the entire population, should the entire population receive the treatment. ATT measures the treatment effect within the group of patients who actually received the treatment. Interested readers are referred to *Greifer et al* [8]. Weights were generated using gradient boosting (GBM), extreme gradient boosting (XGBOOST), Bayesian additive regression trees (BART), neural networks and logistic regression. Each modelling framework was used to model ICD implantation using the 50 top predictors identified by GBM as well as RF, in separate models. Tree-based methods were tuned with regards to tree depth, number of trees and learning rate. Tuning was optimized to minimize covariate standardized mean difference. Standardized mean differences (SMD) below 0.1 were considered non-consequential [9]. The weights were used in a Cox proportional hazards models to calculate hazard ratios for the endpoints (death, recurrent OHCA, IHCA) from discharge to end of follow-up. Subgroup analyses were performed in relation to age, sex, comorbidities and patient characteristics relating to the event.

For peer review only - http://bmjopen.bmj.com/site/about/guidelines.xhtml

Patient and Public Involvement

None.

Ethical consideration

The study was approved by the Swedish Ethical Review Authority.

Results

Baseline characteristics

A total of 3005 survivors of OHCA were included, of whom 959 had an ICD implanted and 2046 were discharged without an ICD. Mean age was 58.5 years and 63.6 years in cases with and without ICD implantation, respectively. Women constituted 21% of cases without ICD implanted, compared with 18% of those with an ICD implanted (Table 1).

A history of hypertension was equally prevalent. Previous ischemic heart disease, dyslipidemia, angina pectoris, atrial fibrillation, heart failure, myocardial infarction and diabetes were more prevalent prior to OHCA in cases who received an ICD. Cardiovascular medications were also more common in patients who received an ICD (Table 1).

Pre-hospital defibrillations were performed in 91.1% of cases who received an ICD, compared with 85.8% of those who did not receive an ICD. Adrenaline and amiodarone were more frequently administered in cases who received an ICD. The initial rhythm was shockable in 96.2% of cases who received an ICD, compared with 92.6% of cases who did not. The vast majority (95.8% and 95.3% in those with and without ICD, respectively) in both groups displayed signs of spontaneous circulation at arrival to hospital. PCI was performed more frequently in those who did not receive an ICD (Table 1).

For peer review only

For peer review only - http://bmjopen.bmj.com/site/about/guidelines.xhtml

Table 1. Baseline characteristics (abbreviated) in patients who were discharged after out-of-hospital cardiac arrest, in relation to ICD implantation

Variable	No ICD	ICD	P-value	SMD
	2046	959		
Women – n (%)	435 (21.3)	173 (18.1)	0.046	0.081
Age – mean (SD)	63.6 (13.7)	58.5 (15.4)	<0.001	0.347
**Coexisting conditions prior to OHCA**				
Hypertension	607 (29.7)	292 (30.4)	0.694	0.017
Ischemic heart disease	280 (13.7)	242 (25.2)	<0.001	0.295
Stable or unstable angina	213 (10.4)	168 (17.5)	<0.001	0.206
Heart failure	181 (8.8)	174 (18.1)	<0.001	0.275
Acute myocardial infarction	178 (8.7)	161 (16.8)	<0.001	0.244
Type 2 diabetes	164 (8.0)	114 (11.9)	0.001	0.130
Stroke	79 (3.9)	33 (3.4)	0.643	0.022
Renal failure	73 (3.6)	32 (3.3)	0.830	0.013
**Medications prior to OHCA**			Nan	
RAAS blockers	548 (26.8)	339 (35.3)	<0.001	0.186
Anticoagulants	405 (19.8)	331 (34.5)	<0.001	0.336
Beta-blockers	434 (21.2)	293 (30.6)	<0.001	0.214
Lipid lowering drugs	349 (17.1)	267 (27.8)	<0.001	0.261
**Location of cardiac arrest**			<0.001	0.477
Home	832 (40.7)	411 (42.9)		
Public place	644 (31.5)	447 (46.6)		
Other places	568 (27.8)	101 (10.5)		
**Prehospital interventions**				
Mechanical compressions	514 (26.2)	257 (27.7)	0.416	0.034

For peer review only - http://bmjopen.bmj.com/site/about/guidelines.xhtml

Intubation	295 (14.7)	147 (15.6)	0.591	0.023
Defibrillated, any	1738 (85.8)	867 (91.1)	<0.001	0.165
Adrenaline	719 (36.0)	411 (43.8)	<0.001	0.161
Amiodarone	352 (17.7)	246 (26.4)	<0.001	0.211
**Critical time intervals, minutes – median (IQR)**			Nan	
Arrest to CPR start	0.00 [0.00, 2.00]	1.00 [0.00, 4.00]	<0.001	0.180
Arrest to first defibrillation	6.00 [1.00, 12.00]	10.00 [6.00, 13.00]	<0.001	0.330
Arrest to ambulance arrival	9.00 [6.00, 13.00]	9.00 [7.00, 14.00]	0.021	0.095
Arrest to ROSC	11.00 [4.00, 18.00]	13.00 [8.50, 21.00]	<0.001	0.263
**Initial rhythm**			0.001	0.167
VF/pVT	1667 (92.6)	852 (96.2)		
PEA	65 (3.6)	12 (1.4)		
Asystole	69 (3.8)	22 (2.5)		
Circulation on hospital arrival	1932 (95.3)	913 (95.8)	0.614	0.024
Conscious on hospital arrival	1021 (50.9)	345 (36.8)	<0.001	0.288
**Circumstances at time of arrest**			Nan	
Witnessed arrest	1879 (92.7)	866 (91.4)	0.283	0.045
Bystander CPR	1080 (54.4)	743 (78.6)	<0.001	0.531
**Inhospital interventions**			Nan	
PCI	1495 (74.0)	364 (38.9)	<0.001	0.756
CABG	99 (4.9)	34 (3.6)	0.148	0.063

Abbreviations:
ROSC = Return of Spontaneous Circulation, PCI = Percutaneous coronary intervention, CABG = Coronary artery bypass grafting, ECMO = Extracorporeal membrane oxygenation, ICD = Implantable cardioverter defibrillator, VF = Ventricular fibrillation, PVT = Pulseless ventricular tachycardia, EMS = Emergency medical system, PEA = Pulseless electrical activity, AED = Automated external defibrillator, EU = European Union.
SMD = Standardized mean difference, is calculated as the difference in means between the two groups, divided by their combined standard deviation. Balance was defined as SMD <0.1.

For peer review only - http://bmjopen.bmj.com/site/about/guidelines.xhtml

Survival

A total of 320 deaths, 138 recurrent OHCAs and 70 IHCAs occurred after discharge. The most pronounced survival trend was noted for cases who did not receive an ICD, of whom the majority of deaths occurred early after discharge (Figure 2). In men and women, 45% and 58%, respectively, of those who experienced the composite endpoint did so within 90 days, with the majority doing so within 30 days. Among those experiencing events, 25% did so within 90 days in the ICD group, compared with 52% within 90 days among the others.

Association between ICD implantation and outcomes

All point estimates, for both ATE and ATT analyses were below 1.0, with the vast majority being significant, favoring ICD implantation (Figure 3).

With regards to ATE, the overall hazard ratio (95% CI) for ICD vs no ICD was 0.38 (0.26-0.56). The point estimate was 0.40 among women, although not statistically significant (8 events occurred in females with ICD). For cases with VF/pVT (pulseless ventricular tachycardia) as initial rhythm HR was 0.42 (0.28-0.63). For cases with PEA/asystole HR was 0.18 (0.06-0.58). HR for those with a history of CAD, heart failure and diabetes were 0.32 (0.20-0.53), 0.36 (0.22-0.61), 0.30 (0.13-0.69), respectively. The lowest point estimates were noted for cases with an acute MI, as well as those who did not undergo PCI or CABG.

With regards to ATT, the hazard ratios for ICD vs no ICD were aligned with those obtained for ATE.

With regards to non-balanced covariate, adding them to the Cox regression did not affect any point estimate (Supplementary Figure 3).

Discussion

Our results clearly demonstrate that those discharged with an ICD were at substantially lower risk of death or recurrent cardiac arrest, with all hazard ratios around 0.3, suggesting a 70% reduction in the probability of death or recurrent arrest. While these results encourage a broader use of ICDs, one cannot exclude confounding by indication given the observational

For peer review only - http://bmjopen.bmj.com/site/about/guidelines.xhtml

nature of the data. We found that the highest incidence rate for death or recurrent cardiac arrest was seen early after discharge (Figure 1). Aligned with these results, a Danish study (concluded during 2007-2011) found that patients with early ICD implantation (during hospitalization) had a higher survival probability post discharge, with hazard ratios similar to ours; hazard ratio for death in the ICD group *vs.* no ICD was 0.44 (95% CI 0.23–0.88) [10]. Other studies have presented similar trends in results [1,2,10,11].

Current clinical guidelines suggests that patients developing ventricular arrythmias due to a cardiac event, in which the underlying condition resolves does not have an increased risk for future SCA, thus not eligible for ICD therapy. However, previous reports have suggested otherwise, showing these individuals have an elevated risk for future malignant arrhythmias [2,7,8]. These ideas are supported by and described in the Antiarrhythmics Versus Implantable Defibrillators (AVID) study [1]. In this study, we approached this issue by examining the average treatment effect (ATE) and the average treatment effect on those treated (ATT) in cases with OHCA of presumed cardiac etiology, or with an initial shockable rhythm.

European clinical guidelines recommends ICD implantation for patients with malignant arrhythmias after an acute myocardial infarction with pre-existing left ventricular dysfunction or incomplete revascularization (*class IIb C indication*) [12-16]. ICD implantation is recommended regardless of etiology for secondary prevention in patients with dilated cardiomyopathy or hypokinetic non-dilated cardiomyopathy in survivors of OHCA with an initial shockable rhythm, or with hemodynamically significant monomorphic ventricular tachycardia (*class I recommendation*) [16]. After a cardiac arrest, patients are followed at an outpatient clinic and if left ventricular ejection fraction is not restored adequately, then an ICD is recommended. However, our study suggests that this initial time period after discharge carries the highest risk for these patients and future guidelines should take this into consideration [11].

In this study, we also performed 15 subgroup analyses (Figure 3) for both ATE and ATT. All analyses demonstrated similar results, with a strong reduction in the probability of death or recurrent cardiac arrest in individuals receiving an ICD. Notable exceptions to this was the non-significance in the subgroups consisting of women, type 1 diabetes, obesity, acute heart

For peer review only - http://bmjopen.bmj.com/site/about/guidelines.xhtml

failure, and those undergoing PCI or CABG. With regards to women, although the ATE estimate was not significant, the ATT was clearly significant (HR 0.30 [95% CI 0.13-0.66]). Regarding individuals with type 1 diabetes, obesity, acute heart failure, PCI or CABG performed, it is likely that we were underpowered to assess these subgroups.

Another interesting finding was that PEA/asystole on presentation was associated with a lower risk of outcome in the ICD group. This may be explained by the fact that ambulance response times are increasing which is strongly associated with reductions in the rate of cases presenting with shockable rhythms [17]. Hence, a significant proportion of those with asystole may have VF/pVT as their instigating event, but deteriorate to non-shockable rhythms by the time the first ECG is recorded. Moreover, in these cases we do not have data on subsequent (after ROSC) occurrences of ventricular arrhythmias, which would increase their likelihood of receiving an ICD.

This study is a registry-based study, which hampers our ability to draw causal inferences. We do not have access to exact timing of ICD implantation, and it is possible that some individuals may have had ICDs implanted prior to cardiac arrest. However our intention-to-treat design emphasizes on having an ICD at discharge. We do not have information on in-hospital ECG monitoring data. Residual confounding and confounding by indication are important caveats of propensity score methods. Confounding by indication in this context implies that the propensity score method may be unable to completely remove confounders related to the decision to implant an ICD (e.g patients having longer expected survival, etc).

The DanICD study (NCT ID NCT04576130) will contribute valuable evidence to this topic. It is a randomized study with the aim to assess whether there is a benefit of ICD-implantation in patients with coronary artery disease (including acute myocardial infarction), who survive cardiac arrest due to ventricular fibrillation/sustained ventricular tachycardia and undergo revascularization and with an LVEF greater than 35%.

Conclusion

Our study suggests that, on a population and individual level, broad use of ICDs are associated with markedly lower risk of death and recurrent cardiac arrest after OHCA. ICD

For peer review only - http://bmjopen.bmj.com/site/about/guidelines.xhtml

therapy was associated with approximately 60% reduction in the probability of these outcomes. Randomized trials are warranted to further unravel the optimal use of ICDs post cardiac arrest.

Funding

This work was supported by the Swedish Research Council (2019–02019), Swedish state under the agreement between the Swedish government, and the county councils (ALFGBG-971482).

Conflict of interest

None declared

Data availability statement

Please contact AR (araz.rawshani@gu.se) for information regarding data availability, which is governed by Swedish law.

Authors' contribution statement

Study concept design and computations were done by Pedram Sultanian and Araz Rawshani. First draft of the manuscript was written by Pedram Sultanian. Peter Lundgren, Aidin Rawshani, Sebastian Möller, Arash Hadi Jafari, Laura David, Shavan Yasinsson, Anna Myredal, Cecilia Rorsman, Amar Taha, Annica Ravn-Fischer, Andreas Martinsson and Johan Herlitz contributed to interpretation of results, refining the manuscript and responding to reviewer concerns.

For peer review only

For peer review only - http://bmjopen.bmj.com/site/about/guidelines.xhtml

References

1. Goldenberg, I. *et al.* Long-term benefit of primary prevention with an implantable cardioverter-defibrillator: an extended 8-year follow-up study of the Multicenter Automatic Defibrillator Implantation Trial II. *Circulation* **122**, 1265–1271 (2010).

2. Hussein, A. A. & Wilkoff, B. L. Cardiac Implantable Electronic Device Therapy in Heart Failure. *Circ Res* **124**, 1584–1597 (2019).

3. Schmidt, A. *et al.* Infarct Tissue Heterogeneity by Magnetic Resonance Imaging Identifies Enhanced Cardiac Arrhythmia Susceptibility in Patients With Left Ventricular Dysfunction. *Circulation* **115**, 2006–2014 (2007).

4. Tamaki, S. *et al.* Cardiac Iodine-123 Metaiodobenzylguanidine Imaging Predicts Sudden Cardiac Death Independently of Left Ventricular Ejection Fraction in Patients With Chronic Heart Failure and Left Ventricular Systolic Dysfunction. *Journal of the American College of Cardiology* **53**, 426–435 (2009).

5. Yan, A. T. *et al.* Characterization of the Peri-Infarct Zone by Contrast-Enhanced Cardiac Magnetic Resonance Imaging Is a Powerful Predictor of Post–Myocardial Infarction Mortality. *Circulation* **114**, 32–39 (2006).

6. Myerburg, R. J. & Junttila, M. J. Sudden Cardiac Death Caused by Coronary Heart Disease. *Circulation* **125**, 1043–1052 (2012).

7. Wyse D.George *et al.* Life-threatening ventricular arrhythmias due to transient or correctable causes: high risk for death in follow-up. *Journal of the American College of Cardiology* **38**, 1718–1724 (2001).

8. Greifer, N. & Stuart, E. A. Choosing the Causal Estimand for Propensity Score Analysis of Observational Studies. Preprint at http://arxiv.org/abs/2106.10577 (2023).

9. Austin, P. C. An Introduction to Propensity Score Methods for Reducing the Effects of Confounding in Observational Studies. *Multivariate Behavioral Research* **46**, 399–424 (2011).

10. Helle Søholm, Early ICD implantation in cardiac arrest survivors with acute coronary syndrome – predictors of implantation, ICD-therapy and long-term survival. Scandinavian Cardiovascular Journal. 2021, VOL. 55, NO. 4, 205-212.

For peer review only

11. Matilde Winther-Jensen et al. Implantable caridioverter defibrillator and survival after out of hospital cardiac arrest due to acute myocardial infarction in Denmark in the years 2001-2012, a nationwide study, European Heart Journal: Acute Cardiovascular Care 2017, Vol 6(2) 144-154

12. Connolly SJ, Gent M, Roberts RS, et al. Canadian Implantable Defibrillator Study (CIDS): a randomized trial of the implantable cardioverter defibrillator against amiodarone. Circulation. 2000;101(11):1297–1302.

13. Kuck KH, Cappato R, Siebels J, et al. Randomized comparison of antiarrhythmic drug therapy with implantable defibrillators in patients resuscitated from cardiac arrest: the Cardiac Arrest Study Hamburg (CASH)). Circulation. 2000;102(7):748–754.

14. Priori SG, Blomstrom-Lundqvist C, Mazzanti A, et al. 2015 ESC Guidelines for the management of patients with ventricular arrhythmias and the prevention of sudden cardiac death: The Task Force for the Management of Patients with Ventricular Arrhythmias and the Prevention of Sudden Cardiac Death of the European Society of Cardiology (ESC). Endorsed by: Association for European Paediatric and Congenital Cardiology (AEPC). Eur Heart J. 2015;36(41):2793–2867.

15. Zipes DP, Camm AJ, Borggrefe M, et al. ACC/AHA/ESC 2006 guidelines for management of patients with ventricular arrhythmias and the prevention of sudden cardiac death: A report of the American College of Cardiology/American Heart Association Task Force and the European Society of Cardiology Committee for Practice Guidelines (Writing Committee to Develop guidelines for management of patients with ventricular arrhythmias and the prevention of sudden cardiac death) developed in collaboration with the European Heart Rhythm Association and the Heart Rhythm Society. Europace. 2006; 8(9):746–837.

16. Task force members of the European Heart Journal, 2022 ESC Guidelines for the management of patients with ventricular arrhythmias and the prevention of sudden cardiac death, European Heart Journal (2022) 43, 3997-4126

17. M Jerkeman, P Sultanian et al. Trends in survival after cardiac arrest: a Swedish nationwide study over 30 years. European Heart Journal, Volume 43, Issue 46, 7 December 2022, Pages 4817–4829

For peer review only

For peer review only - http://bmjopen.bmj.com/site/about/guidelines.xhtml

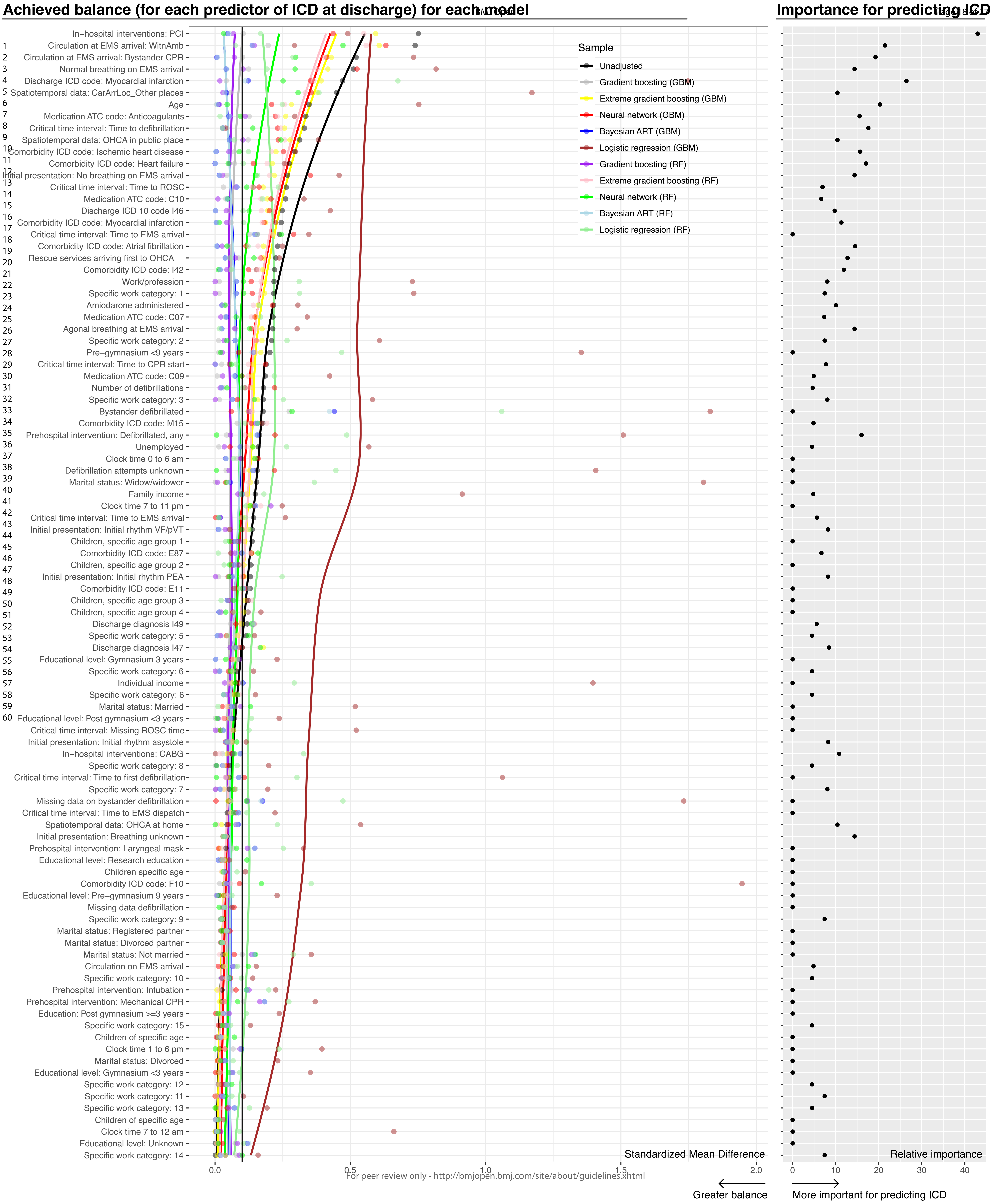

For peer review only - http://bmjopen.bmj.com/site/about/guidelines.xhtml

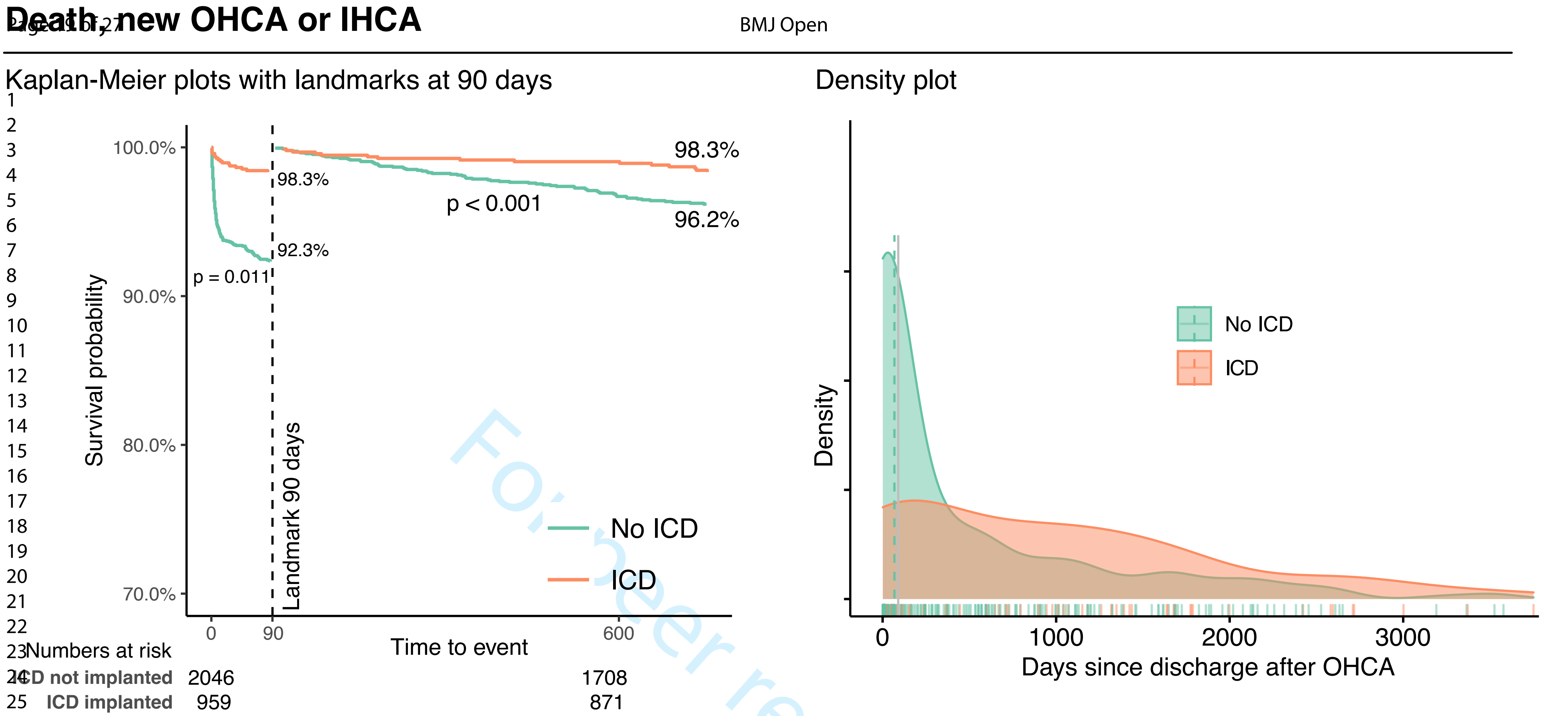

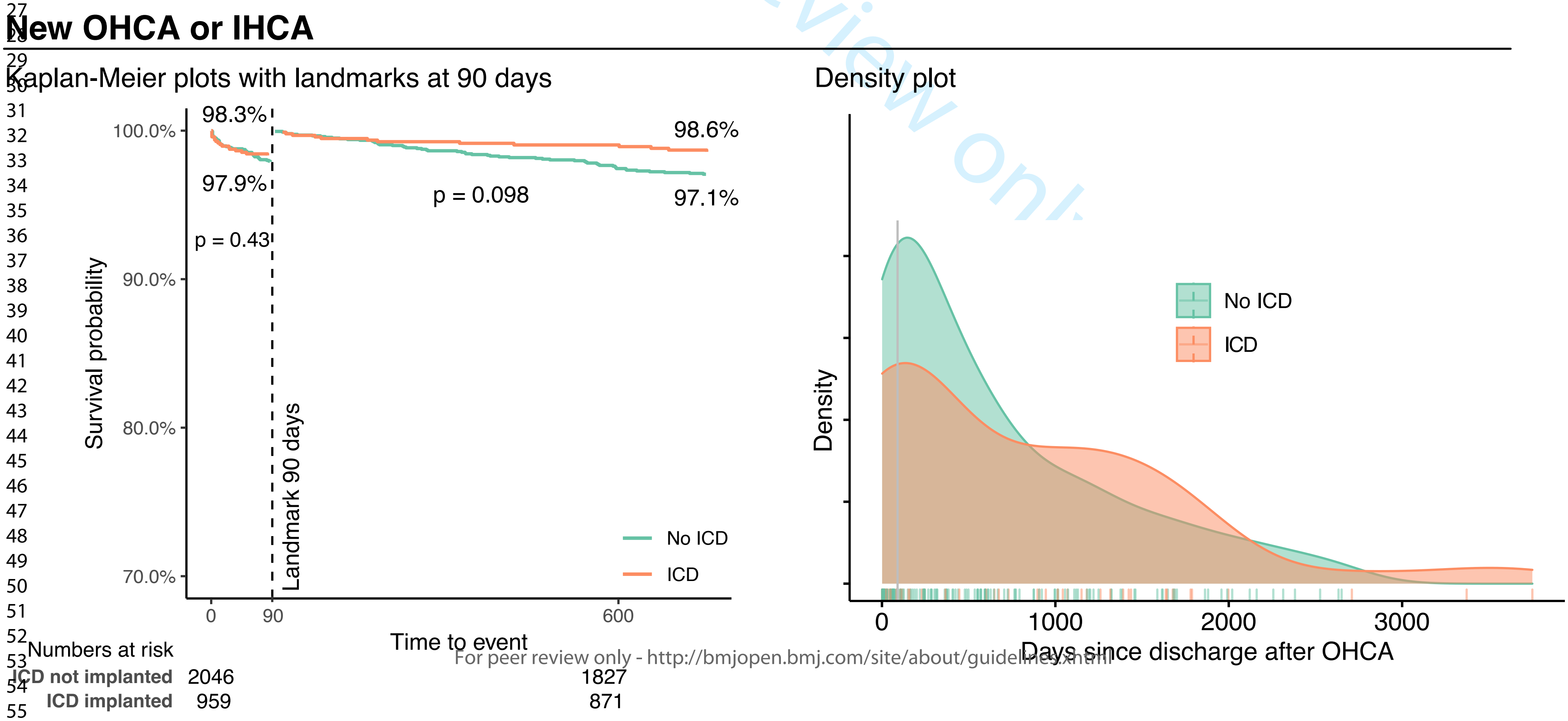

For peer review only

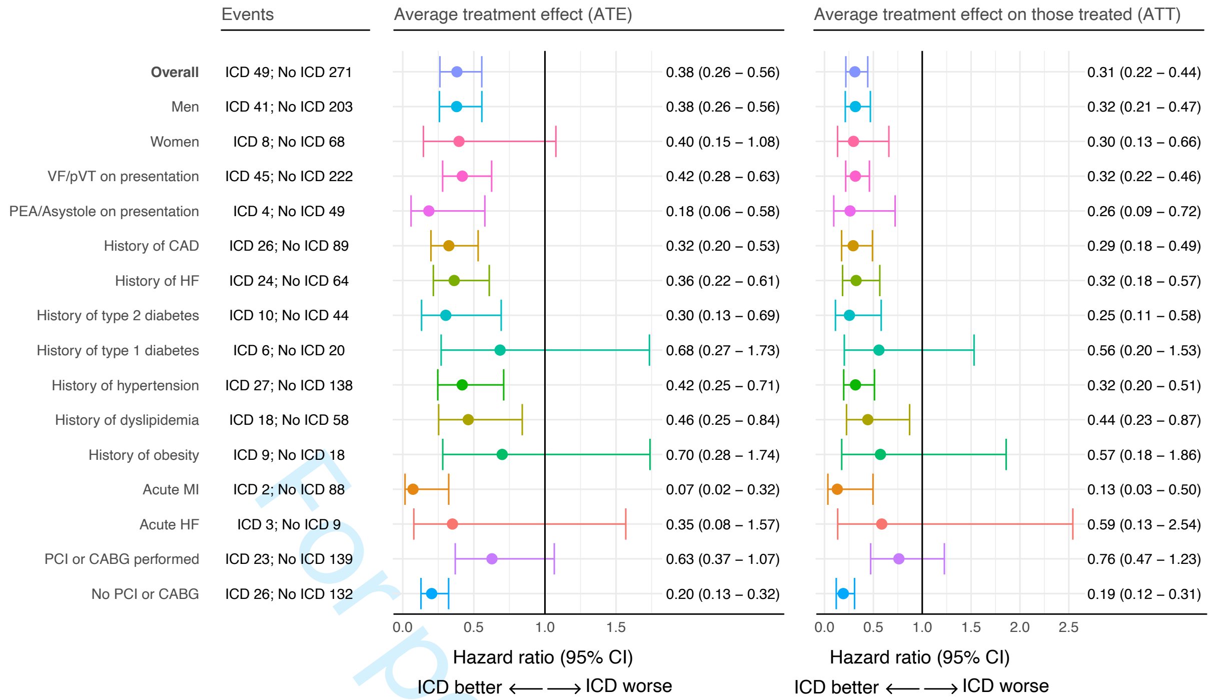

Figure 3. Hazard ratios for death, recurrent OHCA or IHCA, using the final model.

For peer review only

Supplementary Figure 1. Relative importance using Random Forest

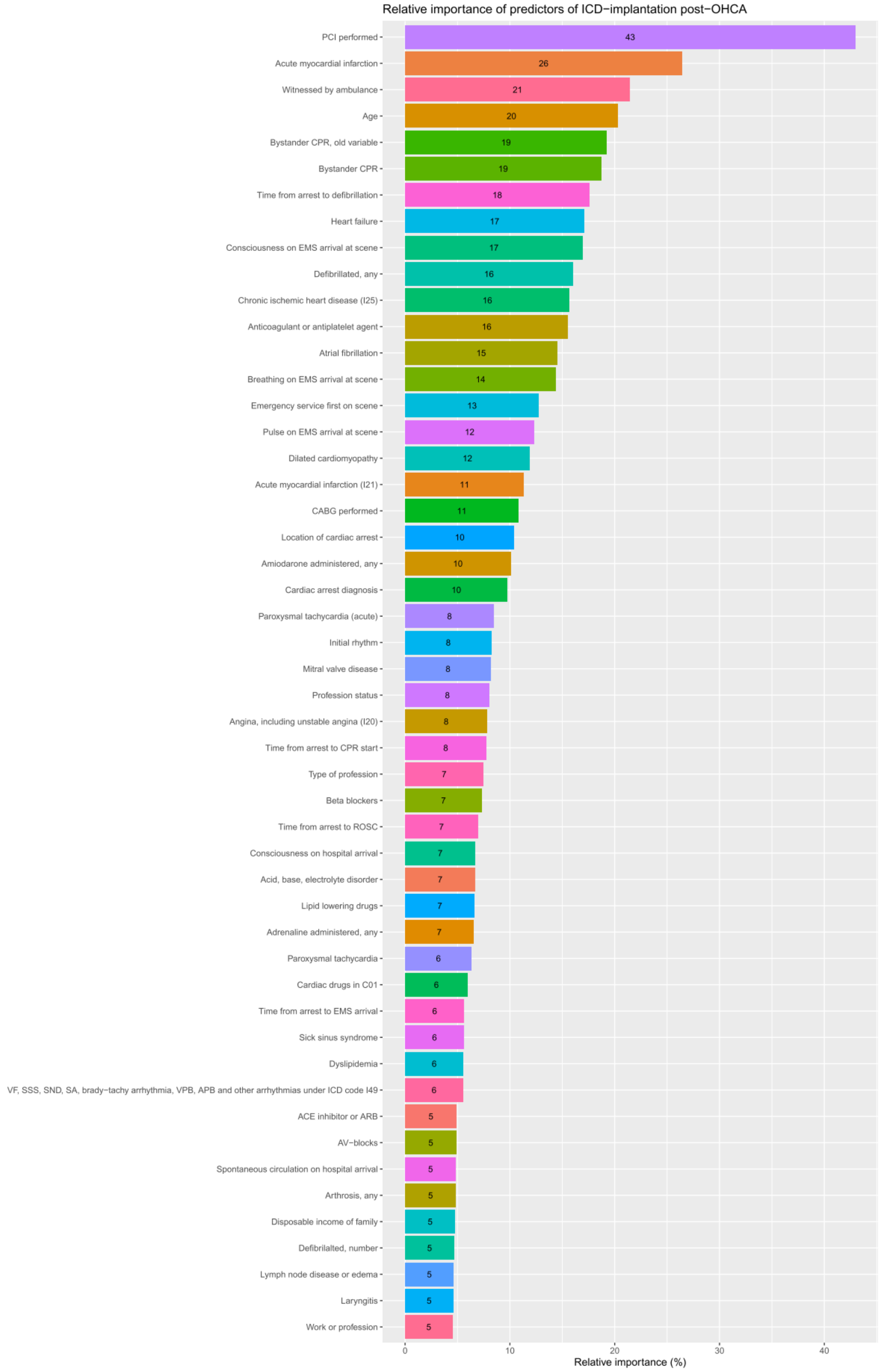

Supplementary Figure 1. Relative importance of top 50 predictors of ICD implantation using random forest. The relative importance is defined as the predictors ability to predict the outcome (i.e. having an ICD at discharge). The direction of the association is not depicted, merely the strength of the association.

Supplementary Figure 2. Relative importance using Gradient Boosting

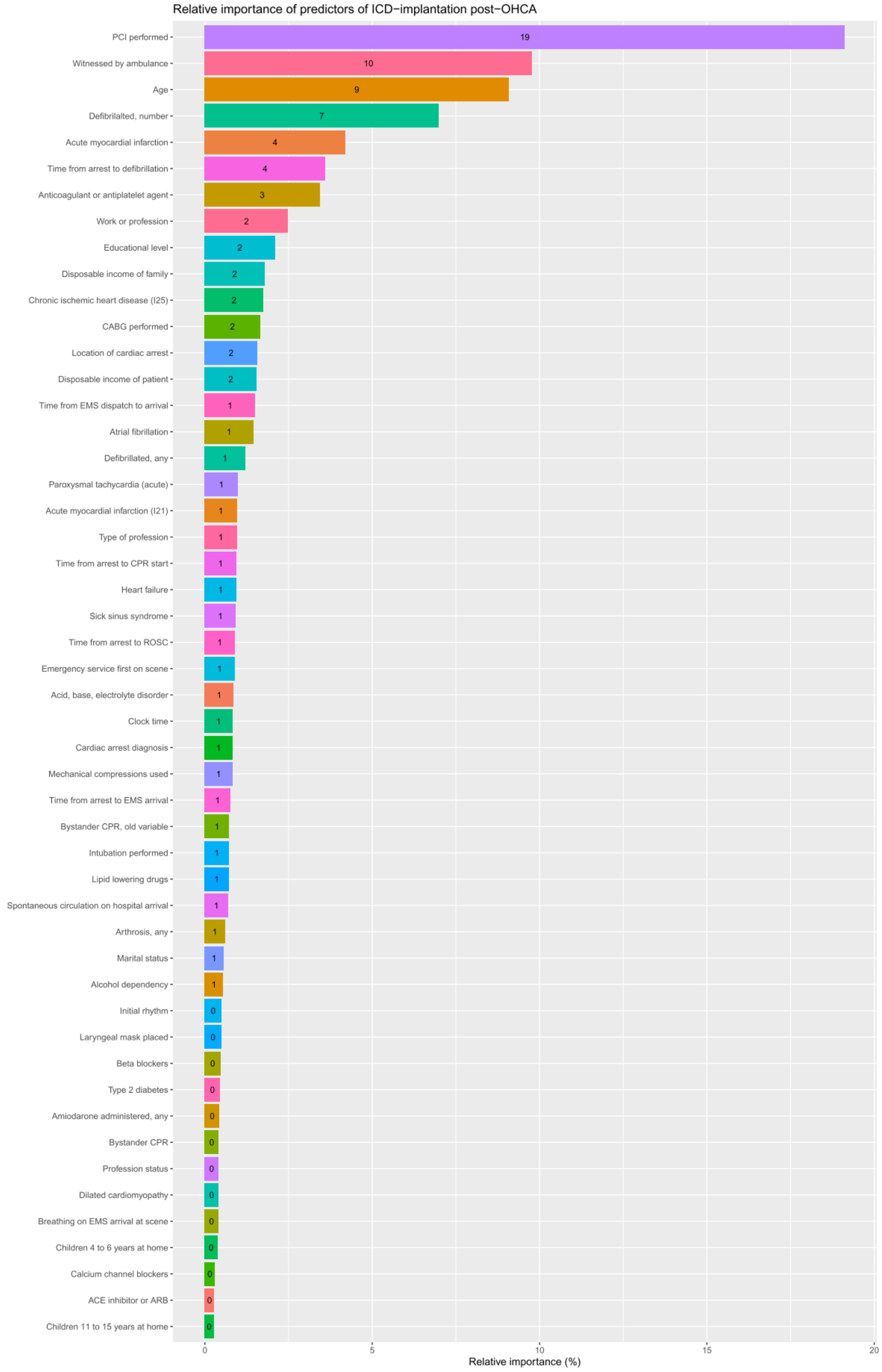

Supplementary Figure 2. Relative importance of top 50 predictors of ICD implantation using gradient boosting. The relative importance is defined as the predictors ability to predict the outcome (i.e. having an ICD at discharge). The direction of the association is not depicted, merely the strength of the association.

Supplementary Figure 3. Cox proportional hazards model for non-balanced covariates

(A)

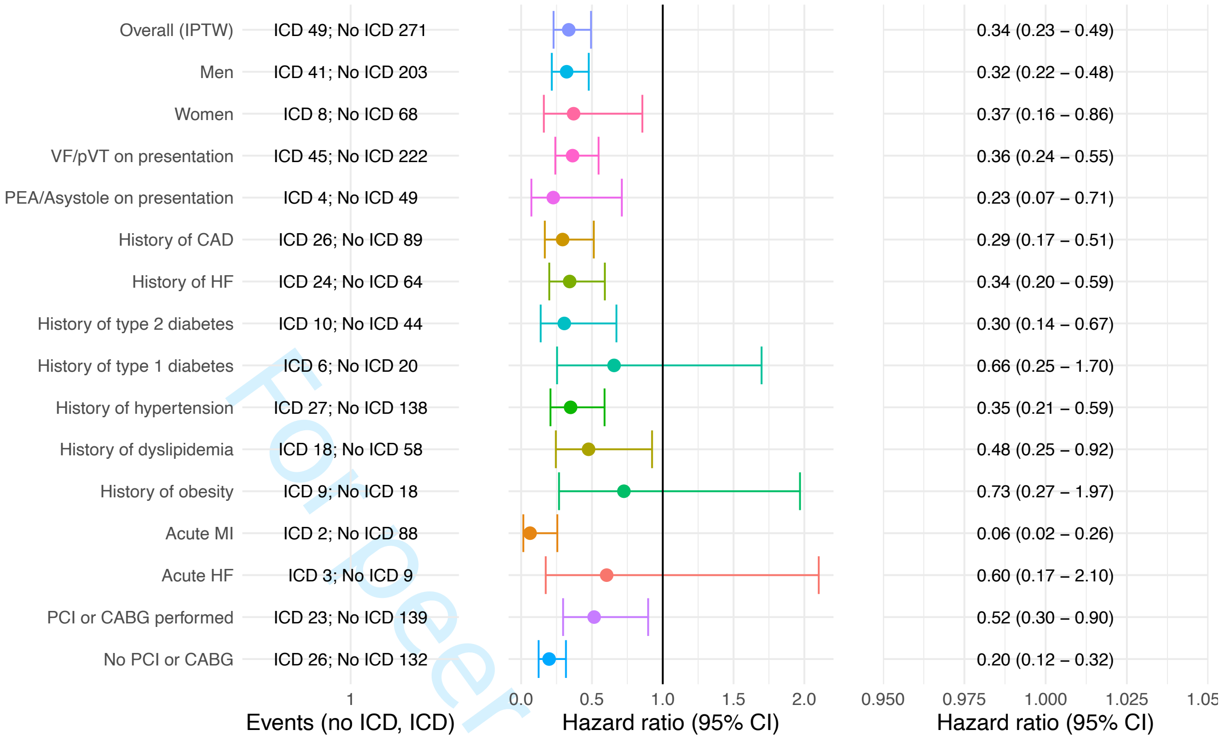

(B)

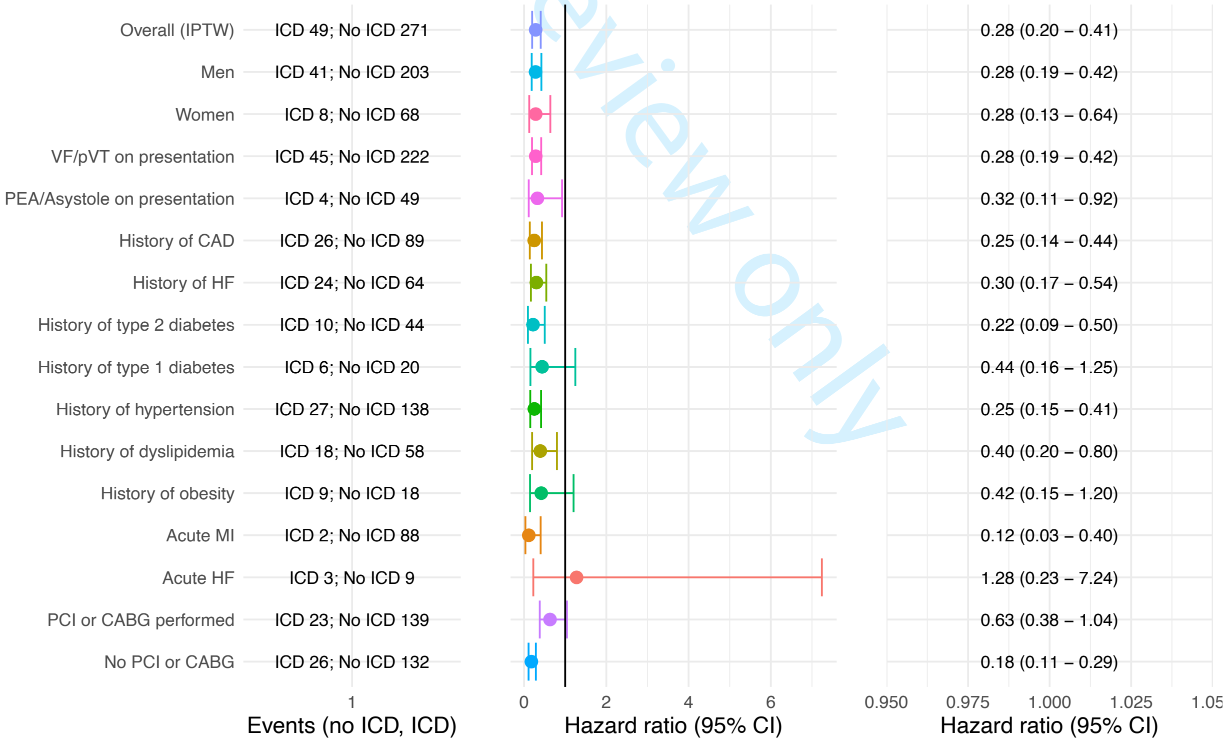

Supplementary figure 3. Cox proportional hazards model for death, recurrent OHCA or IHCA, with adjustments for non-balanced covariates. As evident, adding the non-balanced covariates to the Cox regression did not affect the results. (A) shows results from ATE analysis, and (B) shows results from ATT analysis.

For peer review only

Supplementary Table 1. Baseline characteristics (complete table) in patients who were discharged after out-of-hospital cardiac arrest, in relation to ICD implantation

Variable	No ICD	ICD	P-value	SMD
	2046	959		
Women – n (%)	435 (21.3)	173 (18.1)	0.046	0.081
Age – mean (SD)	63.6 (13.7)	58.5 (15.4)	<0.001	0.347
Born abroad	317 (15.6)	148 (15.5)		
Disposable income, family – median (IQR)	8870.00 [6643.00, 11624.75]	9754.00 [7462.00, 12052.00]	<0.001	0.144
Disposable income, individual – median (IQR)	4454.00 [3062.00, 6171.00]	4951.00 [3353.00, 6296.00]	0.001	0.078
Not employed	978 (52.3)	384 (43.9)		
**Educational level**			0.001	0.203
Pre gymnasium <9 years	344 (18.2)	106 (11.8)		
Post gymnasium 3 years	218 (11.5)	123 (13.7)		
Post gymnasium 3 years or longer	240 (12.7)	124 (13.9)		
Married	1070 (52.7)	540 (56.3)		
**Coexisting conditions prior to OHCA**			Nan	
Hypertension	607 (29.7)	292 (30.4)	0.694	0.017
Ischemic heart disease	280 (13.7)	242 (25.2)	<0.001	0.295
Dyslipidemia	245 (12.0)	171 (17.8)	<0.001	0.165
Stable or unstable angina	213 (10.4)	168 (17.5)	<0.001	0.206
Atrial fibrillation	199 (9.7)	169 (17.6)	<0.001	0.231
Heart failure	181 (8.8)	174 (18.1)	<0.001	0.275
Acute myocardial infarction	178 (8.7)	161 (16.8)	<0.001	0.244
Type 2 diabetes	164 (8.0)	114 (11.9)	0.001	0.130
Alcohol dependency	150 (7.3)	62 (6.5)	0.431	0.034

For peer review only

For peer review only - http://bmjopen.bmj.com/site/about/guidelines.xhtml

Phobic syndromes	125 (6.1)	48 (5.0)	0.260	0.048
Manic episode	114 (5.6)	34 (3.5)	0.021	0.097
Malignant melanoma	93 (4.5)	34 (3.5)	0.241	0.051
Stroke	79 (3.9)	33 (3.4)	0.643	0.022
Transient ischemic attack (TIA)	63 (3.1)	46 (4.8)	0.025	0.088
Renal failure	73 (3.6)	32 (3.3)	0.830	0.013
Aortic stenosis	54 (2.6)	42 (4.4)	0.016	0.095
Type 1 diabetes	60 (2.9)	31 (3.2)	0.739	0.017
**Medications prior to OHCA**			Nan	
RAAS blockers	548 (26.8)	339 (35.3)	<0.001	0.186
Anticoagulants	405 (19.8)	331 (34.5)	<0.001	0.336
Beta-blockers	434 (21.2)	293 (30.6)	<0.001	0.214
Lipid lowering drugs	349 (17.1)	267 (27.8)	<0.001	0.261
Calcium channel blockers	310 (15.2)	105 (10.9)	0.002	0.125
Diuretics	250 (12.2)	129 (13.5)	0.374	0.037
Anti-acidic peptic drugs	209 (10.2)	87 (9.1)	0.360	0.039
**Location of cardiac arrest**			<0.001	0.477
Home	832 (40.7)	411 (42.9)		
Public place	644 (31.5)	447 (46.6)		
Other places	568 (27.8)	101 (10.5)		
**Prehospital interventions**				
Mechanical compressions	514 (26.2)	257 (27.7)	0.416	0.034
Intubation	295 (14.7)	147 (15.6)	0.591	0.023
Laryngeal airway	426 (33.9)	227 (38.7)	0.050	0.100
Defibrillated, any	1738 (85.8)	867 (91.1)	<0.001	0.165

For peer review only - http://bmjopen.bmj.com/site/about/guidelines.xhtml

Defibrillated, times – mean (SD)	2.57 (2.27)	2.99 (2.44)	<0.001	0.179
Adrenaline	719 (36.0)	411 (43.8)	<0.001	0.161
Amiodarone	352 (17.7)	246 (26.4)	<0.001	0.211
**Critical time intervals, minutes – median (IQR)**			Nan	
Arrest to EMS dispatch	2.00 [1.00, 3.00]	2.00 [1.00, 3.00]	0.361	0.065
Arrest to CPR start	0.00 [0.00, 2.00]	1.00 [0.00, 4.00]	<0.001	0.180
Arrest to first defibrillation	6.00 [1.00, 12.00]	10.00 [6.00, 13.00]	<0.001	0.330
Arrest to ambulance arrival	9.00 [6.00, 13.00]	9.00 [7.00, 14.00]	0.021	0.095
Emergency call to ambulance arrival	8.00 [5.00, 13.00]	7.00 [5.00, 11.00]	<0.001	0.245
Arrest to ROSC	11.00 [4.00, 18.00]	13.00 [8.50, 21.00]	<0.001	0.263
**Initial rhythm**			0.001	0.167
VF/pVT	1667 (92.6)	852 (96.2)		
PEA	65 (3.6)	12 (1.4)		
Asystole	69 (3.8)	22 (2.5)		
Circulation on hospital arrival	1932 (95.3)	913 (95.8)	0.614	0.024
Conscious on hospital arrival	1021 (50.9)	345 (36.8)	<0.001	0.288
**Circumstances at time of arrest**			Nan	
Witnessed arrest	1879 (92.7)	866 (91.4)	0.283	0.045
Bystander CPR	1080 (54.4)	743 (78.6)	<0.001	0.531
Telephone CPR	304 (51.3)	233 (57.0)	0.086	0.115
Bystander education			0.643	0.071
Laymen not CPR educated	150 (34.2)	109 (37.3)		
Laymen CPR educated	169 (38.6)	104 (35.6)		

For peer review only - http://bmjopen.bmj.com/site/about/guidelines.xhtml

Health care professional	119 (27.2)	79 (27.1)		
Bystander connected AED	167 (15.0)	93 (16.9)	0.354	0.052
Bystander used AED	128 (76.6)	77 (83.7)	0.239	0.177
Witnessed by ambulance	428 (39.3)	50 (10.0)	<0.001	0.724
Ambulance first on scene	1019 (84.6)	430 (77.2)	<0.001	0.190
**Inhospital interventions**			Nan	
PCI	1495 (74.0)	364 (38.9)	<0.001	0.756
CABG	99 (4.9)	34 (3.6)	0.148	0.063

Abbreviations: ROSC = Return of Spontaneous Circulation, PCI = Percutaneous coronary intervention, CABG = Coronary artery bypass grafting, ECMO = Extracorporeal membrane oxygenation, ICD = Implantable cardioverter defibrillator, VF = Ventricular fibrillation, PVT = Pulseless ventricular tachycardia, EMS = Emergency medical system (ambulance), PEA = Pulseless electrical activity, AED = Automated external defibrillator, EU= European Union. SMD = Standardized mean difference, is calculated as the difference in means between the intervention vs non-intervention group, divided of the SD for the covariate. Balance was considered for SMD < 0.1.

1

For peer review only - http://bmjopen.bmj.com/site/about/guidelines.xhtml

STROBE Statement—checklist of items that should be included in reports of observational studies

	Item No	Recommendation	Page No
**Title and abstract**	1	(*a*) Indicate the study's design with a commonly used term in the title or the abstract	2
		(*b*) Provide in the abstract an informative and balanced summary of what was done and what was found	2
**Introduction**			
Background/rationale	2	Explain the scientific background and rationale for the investigation being reported	4
Objectives	3	State specific objectives, including any prespecified hypotheses	4
**Methods**			
Study design	4	Present key elements of study design early in the paper	4,5
Setting	5	Describe the setting, locations, and relevant dates, including periods of recruitment, exposure, follow-up, and data collection	4,5
Participants	6	(*a*) *Cohort study*—Give the eligibility criteria, and the sources and methods of selection of participants. Describe methods of follow-up<br>*Case-control study*—Give the eligibility criteria, and the sources and methods of case ascertainment and control selection. Give the rationale for the choice of cases and controls<br>*Cross-sectional study*—Give the eligibility criteria, and the sources and methods of selection of participants	4,5
		(*b*) *Cohort study*—For matched studies, give matching criteria and number of exposed and unexposed<br>*Case-control study*—For matched studies, give matching criteria and the number of controls per case	4,5
Variables	7	Clearly define all outcomes, exposures, predictors, potential confounders, and effect modifiers. Give diagnostic criteria, if applicable	4-6
Data sources/ measurement	8*	For each variable of interest, give sources of data and details of methods of assessment (measurement). Describe comparability of assessment methods if there is more than one group	4-6
Bias	9	Describe any efforts to address potential sources of bias	5-6
Study size	10	Explain how the study size was arrived at	4-5
Quantitative variables	11	Explain how quantitative variables were handled in the analyses. If applicable, describe which groupings were chosen and why	5-6
Statistical methods	12	(*a*) Describe all statistical methods, including those used to control for confounding	5-6
		(*b*) Describe any methods used to examine subgroups and interactions	5-6
		(*c*) Explain how missing data were addressed	4-5
		(*d*) *Cohort study*—If applicable, explain how loss to follow-up was addressed<br>*Case-control study*—If applicable, explain how matching of cases and controls was addressed<br>*Cross-sectional study*—If applicable, describe analytical methods taking account of sampling strategy	
		(*e*) Describe any sensitivity analyses	

Continued on next page

For peer review only - http://bmjopen.bmj.com/site/about/guidelines.xhtml

For peer review only

**Results**			
Participants	13*	(a) Report numbers of individuals at each stage of study—eg numbers potentially eligible, examined for eligibility, confirmed eligible, included in the study, completing follow-up, and analysed	7,10
		(b) Give reasons for non-participation at each stage	
		(c) Consider use of a flow diagram	
Descriptive data	14*	(a) Give characteristics of study participants (eg demographic, clinical, social) and information on exposures and potential confounders	7,10
		(b) Indicate number of participants with missing data for each variable of interest	
		(c) *Cohort study*—Summarise follow-up time (eg, average and total amount)	
Outcome data	15*	*Cohort study*—Report numbers of outcome events or summary measures over time	7,10
		Case-control study—Report numbers in each exposure category, or summary measures of exposure	
		Cross-sectional study—Report numbers of outcome events or summary measures	
Main results	16	(*a*) Give unadjusted estimates and, if applicable, confounder-adjusted estimates and their precision (eg, 95% confidence interval). Make clear which confounders were adjusted for and why they were included	7,10
		(*b*) Report category boundaries when continuous variables were categorized	
		(*c*) If relevant, consider translating estimates of relative risk into absolute risk for a meaningful time period	
Other analyses	17	Report other analyses done—eg analyses of subgroups and interactions, and sensitivity analyses	10
**Discussion**			
Key results	18	Summarise key results with reference to study objectives	10
Limitations	19	Discuss limitations of the study, taking into account sources of potential bias or imprecision. Discuss both direction and magnitude of any potential bias	12
Interpretation	20	Give a cautious overall interpretation of results considering objectives, limitations, multiplicity of analyses, results from similar studies, and other relevant evidence	12
Generalisability	21	Discuss the generalisability (external validity) of the study results	12
**Other information**			
Funding	22	Give the source of funding and the role of the funders for the present study and, if applicable, for the original study on which the present article is based	13

*Give information separately for cases and controls in case-control studies and, if applicable, for exposed and unexposed groups in cohort and cross-sectional studies.

Note: An Explanation and Elaboration article discusses each checklist item and gives methodological background and published examples of transparent reporting. The STROBE checklist is best used in conjunction with this article (freely available on the Web sites of PLoS Medicine at http://www.plosmedicine.org/, Annals of Internal Medicine at http://www.annals.org/, and Epidemiology at http://www.epidem.com/). Information on the STROBE Initiative is available at www.strobe-statement.org.

For peer review only

For peer review only - http://bmjopen.bmj.com/site/about/guidelines.xhtml

Early ICD implantation following out-of-hospital cardiac arrest – A retrospective cohort study from the Swedish Registry for Cardiopulmonary Resuscitation

Journal:	*BMJ Open*
Manuscript ID	bmjopen-2023-077137.R2
Article Type:	Original research
Date Submitted by the Author:	21-Dec-2023
Complete List of Authors:	Sultanian, Pedram; University of Gothenburg, Department of Molecular and Clinical Medicine<br>Lundgren, Peter; University of Gothenburg, Department of molecular and clinical medicine; Sahlgrenska University Hospital, Department of Cardiology<br>Rawshani, Aidin; University of Gothenburg, Department of molecular and clinical medicine<br>Möller, Sebastian; Sahlgrenska University Hospital, Department of Cardiology<br>Jafari, Arash Hadi; Halmstad Hospital, Department of Cardiology<br>David, Laura; Sahlgrenska University Hospital, Department of Cardiology<br>Yassinson, Shavan; Kungälvs sjukhus, Department of Cardiology<br>Myredal, Anna; University of Gothenburg, Department of molecular and clinical medicine<br>Rorsman, Cecilia; Varberg Sjukhus, Department of Cardiology<br>Taha, Amar; University of Gothenburg, Department of molecular and clinical medicine; Sahlgrenska University Hospital, Department of Cardiology<br>Ravn-Fischer, Annica; Institution of Molecular and Clinical Medicine, Sahlgrenska University Hospital<br>Martinsson, Andreas; University of Gothenburg, Department of molecular and clinical medicine; Sahlgrenska University Hospital, Department of Cardiology<br>Herlitz, Johan; Faculty of Caring Science, Work Life and Social Welfare, University of Borås<br>Rawshani, Araz; Institute of Medicine, Department of Molecular and Clinical Medicine
**Primary Subject Heading**:	Cardiovascular medicine
Secondary Subject Heading:	Epidemiology
Keywords:	Out-of-Hospital Cardiac Arrest, Cardiac Epidemiology < CARDIOLOGY, Cardiopulmonary Resuscitation

SCHOLARONE™
Manuscripts

I, the Submitting Author has the right to grant and does grant on behalf of all authors of the Work (as defined in the below author licence), an exclusive licence and/or a non-exclusive licence for contributions from authors who are: i) UK Crown employees; ii) where BMJ has agreed a CC-BY licence shall apply, and/or iii) in accordance with the terms applicable for US Federal Government officers or employees acting as part of their official duties; on a worldwide, perpetual, irrevocable, royalty-free basis to BMJ Publishing Group Ltd ("BMJ") its licensees and where the relevant Journal is co-owned by BMJ to the co-owners of the Journal, to publish the Work in this journal and any other BMJ products and to exploit all rights, as set out in our licence.

The Submitting Author accepts and understands that any supply made under these terms is made by BMJ to the Submitting Author unless you are acting as an employee on behalf of your employer or a postgraduate student of an affiliated institution which is paying any applicable article publishing charge ("APC") for Open Access articles. Where the Submitting Author wishes to make the Work available on an Open Access basis (and intends to pay the relevant APC), the terms of reuse of such Open Access shall be governed by a Creative Commons licence – details of these licences and which Creative Commons licence will apply to this Work are set out in our licence referred to above.

Other than as permitted in any relevant BMJ Author's Self Archiving Policies, I confirm this Work has not been accepted for publication elsewhere, is not being considered for publication elsewhere and does not duplicate material already published. I confirm all authors consent to publication of this Work and authorise the granting of this licence.

For peer review only

Early ICD implantation following out-of-hospital cardiac arrest – A retrospective cohort study from the Swedish Registry for Cardiopulmonary Resuscitation

[1]Department of molecular and clinical medicine, Institute of medicine, University of Gothenburg, Gothenburg, Sweden

[2]The Swedish Registry for Cardiopulmonary Resuscitation, Gothenburg, Sweden

[3]Department of Cardiology, Sahlgrenska University Hospital, Gothenburg, Sweden

[4]Department of Cardiology, Halmstad Hospital, Halmstad, Sweden

[5]Department of Cardiology, Varberg Sjukhus, Varberg, Sweden

[6]Department of Cardiology, Kungälvs Sjukhus, Kungälv, Sweden

[7]The Wallenberg Centre for Molecular and Translational Medicine, Gothenburg, Sweden

Pedram Sultanian[1*], Peter Lundgren[1,3], Aidin Rawshani[1], Sebastian Möller[3], Arash Hadi Jafari[4], Laura David[3], Shavan Yassinson[6], Anna Myredal[1], Cecilia Rorsman[5], Amar Taha[1,3], Annica Ravn-Fischer[1,3], Andreas Martinsson[1,3], Johan Herlitz[1,2], Araz Rawshani[1-3]

* Corresponding author. Department of Molecular and Clinical Medicine, University of Gothenburg, Bruna stråket 16, 413 45 Gothenburg, Sweden. Tel: +46 735836248, Email: pedram_sultanian@hotmail.com

For peer review only

Abstract

Background: It is unclear whether an implantable cardioverter-defibrillator (ICD) is generally beneficial in survivors of out-of-hospital cardiac arrest (OHCA).

Objective: We studied the association between ICD implantation prior to discharge and survival in patients with cardiac etiology or initial shockable rhythm in OHCA.

Design: We conducted a retrospective cohort study in the Swedish Registry for Cardiopulmonary Resuscitation. Treatment associations were estimated using propensity scores. We used gradient boosting, Bayesian additive regression trees, neural networks, extreme gradient boosting and logistic regression to generate multiple propensity scores. We selected the model yielding maximum covariate balance to obtain weights, which were used in a Cox regression to calculate hazard ratios (HR) for death or recurrent cardiac arrest.

Participants: All cases discharged alive during 2010 to 2020 with a cardiac etiology or initial shockable rhythm were included. A total of 959 individuals were discharged with an ICD, and 2,046 were discharged without one.

Results: Among those experiencing events, 25% did so within 90 days in the ICD group, compared with 52% in the other group. All HRs favored ICD implantation. The overall HR (95% CI) for ICD vs no ICD was 0.38 (0.26-0.56). The HR was 0.42 (0.28-0.63) in cases with initial shockable rhythm; 0.18 (0.06-0.58) in non-shockable rhythm; 0.32 (0.20-0.53) in cases with a history of coronary artery disease; 0.36 (0.22-0.61) in heart failure and 0.30 (0.13-0.69) in those with diabetes. Similar associations were noted in all subgroups.

Conclusion: Among survivors of OHCA, those discharged with an ICD had approximately 60% lower risk of death or recurrent cardiac arrest. A randomized trial is warranted to study this further.

For peer review only

Strengths and limitations of this study

- **Large and comprehensive dataset**: The study included all cases enrolled in a nationwide registry, with very high coverage, including cases over a long period (2010-2020).
- **State-of-the-art data analysis**: We used a comprehensive data driven machine learning approach to generate propensity scores, and based our estimates on the score yielding maximum covariate balance.
- **Retrospective cohort study, the nature of data**: This study is a retrospective cohort study which limits causal inferences.
- **Timing of ICD implantation**: ICD at discharge was defined as having an ICD at hospital discharge, without specifying when the ICD had been implanted, such that the results should be interpreted as the association between having an ICD at discharge and survival.

For peer review only

Introduction

An implantable cardioverter-defibrillator (ICD) is indicated in primary and secondary prevention of sudden cardiac arrest (SCA) from ventricular fibrillation (VF) or ventricular tachycardia (VT). ICDs are considered in patients with persisting significant risk of VT/VF if guideline-directed medical therapy (GDMT) and invasive procedures are insufficient to eliminate future risk of VF/VT. Several randomized trials have compared ICD with GDMT for primary prevention of SCA. With regards to secondary prevention, the

Antiarrhythmics Versus Implantable Defibrillators (AVID) demonstrated that an ICD is superior to amiodarone for preventing a recurrent cardiac arrest [1,2]. Guidelines currently recommend that an ICD should be considered in cases without transient or reversible causes of SCA due to VF/VT, hemodynamically unstable VT, sustained VT, provided that life-expectancy is 1 year or longer.

It is still unclear whether SCA and ventricular arrhythmias during an acute myocardial infarction should prompt ICD implantation. It is currently believed that such cases are not at significant future risk of SCA. However, the underlying substrate (myocardial scar tissue) may persist even if revascularization is successful. A range of factors that are not routinely assessed may influence future risk of ventricular arrhythmias [1-8]. This was presumably demonstrated in the AVID registry, which showed that patients not randomized to ICD, with transient or reversible causes of SCA, had comparable or possibly inferior outcomes compared to those randomized to ICD [7].

The aim of this study was to study the association between having an ICD at discharge and survival among patients surviving an out-of-hospital cardiac arrest (OHCA) due to cardiac etiology or with an initial shockable rhythm.

Methods

All cases enrolled in the Swedish Registry for Cardiopulmonary Resuscitation (SRCR) from Jan 1st 2010 to June 11th 2020 were assessed (these dates were arbitrarily chosen to include roughly 10 years, and we did not have data beyond the dates the ethical approval was submitted). This included a total of 54956 cases

For peer review only

of OHCA, of whom 54568 were first events. We excluded all recurring events (n=388), such that only the first OHCA was assessed for each individual. A total of 10836 cases had information on ICD, of whom 3851 were discharged alive. Among these 3851 patients we included those with cardiac etiology or initial shockable rhythm, resulting in a final study population of 3005 patients.

Exposure

Implantation of an ICD during hospitalization was recorded as a binary variable in the registry.

Outcomes

The primary outcomes were the composite of death, recurrent OHCA or IHCA (in-hospital cardiac arrest). Mortality data was retrieved from the Swedish Cause of Death Registry, which has complete ascertainment. Recurrent OHCA and new IHCA events were retrieved using the SRCR, with follow-up ending in June 11th 2020.

Variable selection

A total of 919 candidate predictors were assessed. These included 41 predictors relating to patient phenotype, initial presentation, pre-hospital and in-hospital management (43 predictors), socioeconomic variables (43 predictors), medications (19 agents), previous co-existing conditions and diagnoses received during index hospitalization (814 diagnoses). Refer to Table 1 and Supplementary Table 1 for further details. We removed variables with zero variance, as well as factor variables with >30 levels. With regards to the latter, all variables represented socioeconomic data, which was still adequately represented by remaining variables. The final data set contained 438 candidate predictors of ICD implantation.

Feature selection

We used both random forest and gradient boosting to identify the 50 most important predictors of ICD implantation, refer to Supplementary Figure 1 and 2. This was done by computing two separate models with all 438 predictors, using ICD implantation as the dependent variable. The random number generator was not fixed in order to ensure reproducibility across computations, which we confirmed. We checked the consistency between random forest and gradient boosting, knowing that these methods yield

For peer review only

different variable importance. Thus, we used random forest and gradient boosting to identify – according to each algorithm – the top 50 predictors of ICD implantation. These 50 predictors were then used in several models (outlined below) to obtain several propensity scores, in order to find the model, maximizing covariate balance.

Propensity score and inverse probability of treatment weighting (IPTW)

We estimated propensity scores and generated balancing weights to estimate the average treatment effect (ATE) and average treatment effect on the treated (ATT). ATE measures the average treatment effect in the entire population, should the entire population receive the treatment. ATT measures the treatment effect within the group of patients who actually received the treatment. Interested readers are referred to *Greifer et al* [8]. Weights were generated using gradient boosting (GBM), extreme gradient boosting (XGBOOST), Bayesian additive regression trees (BART), neural networks and logistic regression. Each modelling framework was used to model ICD implantation using the 50 top predictors identified by GBM as well as RF, as explained above. Tree-based methods were tuned with regards to tree depth, number of trees and learning rate. Tuning was optimized to minimize covariate standardized mean difference. Standardized mean differences (SMD) below 0.1 were considered non-consequential [9]. The weights were used in Cox proportional hazards models to calculate hazard ratios for the endpoints (death, recurrent OHCA, IHCA) from discharge to end of follow-up. Subgroup analyses were performed in relation to age, sex, comorbidities and patient characteristics relating to the event.

The purpose of our approach to estimate the final propensity score was to consider all available variables, identify the most important ones and then consider several different prediction models to finally obtain the maximum covariate balance. Ultimately, this allows us to estimate the effect of having an ICD at discharge, on the risk of the outcomes (composite of death, recurrent OHCA or IHCA), as can be estimated using observational data.

Patient and Public Involvement

None.

Ethical consideration

For peer review only

The study was approved by the Swedish Ethical Review Authority (#2020-02017). The Ethical Review Authority exempted the need for informed consent, due to the retrospective nature of data.

Results

Baseline characteristics

A total of 3005 survivors of OHCA were included, of whom 959 had an ICD implanted and 2046 were discharged without an ICD. Mean age was 58.5 years and 63.6 years in cases with and without ICD implantation, respectively. Women constituted 21% of cases without ICD implanted, compared with 18% of those with an ICD implanted (Table 1).

A history of hypertension was equally prevalent. Previous ischemic heart disease, dyslipidemia, angina pectoris, atrial fibrillation, heart failure, myocardial infarction and diabetes were more prevalent prior to OHCA in cases who received an ICD. Cardiovascular medications were also more common in patients who received an ICD (Table 1).

Pre-hospital defibrillations were performed in 91.1% of cases who received an ICD, compared with 85.8% of those who did not receive an ICD. Adrenaline and amiodarone were more frequently administered in cases who received an ICD. The initial rhythm was shockable in 96.2% of cases who received an ICD, compared with 92.6% of cases who did not. The vast majority (95.8% and 95.3% in those with and without ICD, respectively) in both groups displayed signs of spontaneous circulation at arrival to hospital. PCI was performed more frequently in those who did not receive an ICD (Table 1).

Survival

A total of 320 deaths, 138 recurrent OHCAs and 70 IHCAs occurred after discharge. The most pronounced survival trend was noted for cases who did not receive an ICD, of whom the majority of deaths occurred early after discharge (Figure 1). In men and women, 45% and 58%, respectively, of those who experienced the composite endpoint did so within 90 days, with the majority doing so within 30 days. Among those experiencing events, 25% did so within 90 days in the ICD group, compared with 52% within 90 days among the others.

For peer review only

Modelling results

Random forest (RF) and gradient boosting (GBM) identified (in separate models) 50 predictors with greatest influence in ICD implantation. The predictors not shared by both frameworks were the following: educational level, individual income, time from emergency dispatch to EMS arrival, clock at time of cardiac arrest, use of mechanical compressions, intubation, use of laryngeal intubation, marital status, alcohol misuse, type 2 diabetes, children aged 4 to 6 and 11 to 15 at home, use of calcium channel blockers.

Covariate balance

Covariate balance obtained using GBM and top 50 predictors identified by RF yielded the best covariate balance, as judged by differences in standardized mean difference, coefficient of variation and distribution of weights. Bayesian additive regression trees yielded the second best balance. Figure 2 displays the unadjusted and weight-adjusted covariate balance across all ten models (left panel), along with the RF derived variable importance (right panel). After adjusting for weights, the GBM model had not balanced the following covariates: history of arthrosis, breathing at EMS arrival, OHCA witnessed by ambulance, use of anticoagulants prior to OHCA, place of cardiac arrest and arrhythmias during hospitalization. The latter was the 41st most important predictor, and included diagnoses such as sick sinus syndrome, brady-tachy arrhythmias, premature beats and ventricular fibrillation (ICD-10 code I.49). That variable was not among the 50 most important predictors for the GBM model.

Association between ICD implantation and outcomes

All point estimates, for both ATE and ATT analyses were below 1.0, with the vast majority being significant, favoring ICD implantation (Figure 3).

With regards to ATE, the overall hazard ratio (95% CI) for ICD vs no ICD was 0.38 (0.26-0.56). The point estimate was 0.40 among women, although not statistically significant (8 events occurred in females with ICD). For cases with VF/pVT (pulseless ventricular tachycardia) as initial rhythm HR was 0.42 (0.28-0.63). For cases with PEA/asystole HR was 0.18 (0.06-0.58). HR for those with a history of CAD, heart failure and diabetes were 0.32 (0.20-0.53), 0.36 (0.22-0.61), 0.30 (0.13-0.69), respectively. The lowest point estimates were noted for cases with an acute MI, PEA/asystole on presentation and those who did not undergo PCI or CABG.

For peer review only

With regards to ATT, the hazard ratios for ICD vs no ICD were aligned with those obtained for ATE.

With regards to non-balanced covariate, adding them to the Cox regression did not affect any point estimate (Supplementary Figure 3).

For peer review only

Discussion

Our results clearly demonstrate that those discharged with an ICD were at substantially lower risk of death or recurrent cardiac arrest, with all hazard ratios around 0.3, suggesting a 70% reduction in the probability of death or recurrent arrest. While these results encourage a broader use of ICDs, one cannot exclude confounding by indication given the observational nature of the data. We found that the highest incidence rate for death or recurrent cardiac arrest was seen early after discharge (Figure 1). Aligned with these results, a Danish study (concluded during 2007-2011) found that patients with early ICD implantation (during hospitalization) had a higher survival probability post discharge, with hazard ratios similar to ours; hazard ratio for death in the ICD group *vs.* no ICD was 0.44 (95% CI 0.23–0.88) [10]. Other studies have presented similar trends in results [1,2,10,11].

Current clinical guidelines suggests that patients suffering from ventricular arrythmias due to a resolving cardiac event do not have an increased risk for future SCA. Thus, excluding these patients from receiving treatment with an ICD. However, previous reports have suggested otherwise, showing these individuals

have an elevated risk for future malignant arrhythmias [2,7,8]. These ideas are supported by and described in the Antiarrhythmics Versus Implantable Defibrillators (AVID) study [1]. In this study, we approached this issue by examining the average treatment effect (ATE) and the average treatment effect on those treated (ATT) in cases with OHCA of presumed cardiac etiology, or with an initial shockable rhythm.

European clinical guidelines recommends ICD implantation for patients with malignant arrhythmias after an acute myocardial infarction with pre-existing left ventricular dysfunction or incomplete revascularization (*class IIb C indication*) [12-16]. ICD implantation is recommended regardless of etiology for secondary prevention in patients with dilated cardiomyopathy or hypokinetic non-dilated cardiomyopathy in survivors of OHCA with an initial shockable rhythm, or with hemodynamically significant monomorphic ventricular tachycardia (*class I recommendation*) [16]. After a cardiac arrest, patients are followed at an outpatient clinic and if left ventricular ejection fraction is not restored adequately, then an ICD is recommended. However, our study suggests that this initial time period after discharge carries the highest risk for these patients and future guidelines should take this into consideration [11].

In this study, we also performed 15 subgroup analyses (Figure 3) for both ATE and ATT. All analyses demonstrated similar results, with a strong reduction in the probability of death or recurrent cardiac arrest in individuals receiving an ICD. Notable exceptions to this was the non-significance in the subgroups consisting of women, type 1 diabetes, obesity, acute heart failure, and those undergoing PCI or CABG. With regards to women, although the ATE estimate was not significant, the ATT was clearly significant (HR 0.30 [95% CI 0.13-0.66]). Regarding individuals with type 1 diabetes, obesity, acute heart failure, PCI or CABG performed, it is likely that we were underpowered to assess these subgroups.

Another interesting finding was that PEA/asystole on presentation was associated with a lower risk of outcome in the ICD group. This may be explained by the fact that ambulance response times are increasing which is strongly associated with reductions in the rate of cases presenting with shockable rhythms [17]. Hence, a significant proportion of those with asystole may have VF/pVT as their instigating event, but deteriorate to non-shockable rhythms by the time the first ECG is recorded. Moreover, in these cases we do not have data on subsequent (after ROSC) occurrences of ventricular arrhythmias, which would increase their likelihood of receiving an ICD.

For peer review only

This study is a registry-based study, which hampers our ability to draw causal inferences. We do not have access to exact timing of ICD implantation, and it is possible that some individuals may have had ICDs implanted prior to cardiac arrest. However our intention-to-treat design emphasizes on having an ICD at discharge. We do not have information on in-hospital ECG monitoring data. Residual confounding and confounding by indication are important caveats of propensity score methods. Confounding by indication in this context implies that the propensity score method may be unable to completely remove confounders related to the decision to implant an ICD (e.g patients having longer expected survival, etc). Even with these considerations, the propensity score, especially when generated through machine learning techniques, stands out as a leading method for imitating a randomized controlled trial using observational data [18,19]. This approach is especially pertinent when utilizing real-world data that encompasses the whole population targeted for inferences, as in this national registry. In regard to missing data, we found that the rate for ICD implantation was around 15% among survivors with cardiac etiology. Missingness may affect the robustness of our estimates, although we argue that the pronounced and consistent reduction of the outcomes cannot be explained by missingness which is more likely to be due to randomness.

The DanICD study (NCT ID NCT04576130) will contribute valuable evidence to this topic. It is a randomized study with the aim to assess whether there is a benefit of ICD-implantation in patients with coronary artery disease (including acute myocardial infarction), who survive cardiac arrest due to ventricular fibrillation/sustained ventricular tachycardia and undergo revascularization and with an LVEF greater than 35%.

Conclusion

Our study suggests that, on a population and individual level, broad use of ICDs are associated with markedly lower risk of death and recurrent cardiac arrest after OHCA. ICD therapy was associated with approximately 60% reduction in the probability of these outcomes. Randomized trials are warranted to further unravel the optimal use of ICDs post cardiac arrest.

Funding

This work was supported by the Swedish Research Council (2019–02019), Swedish state under the agreement between the Swedish government, and the county councils (ALFGBG-971482).

For peer review only

Conflict of interest

None declared

Data availability statement

Please contact AR (araz.rawshani@gu.se) for information regarding data availability, which is governed by Swedish law.

Authors' contribution statement

Study concept, design and calculations were done by Pedram Sultanian and Araz Rawshani. First draft of the manuscript was written by Pedram Sultanian. Araz Rawshani ensured data acquisition.

Peter Lundgren, Aidin Rawshani, Sebastian Möller, Johan Herlitz, Laura David, Anna Myredal, Cecilia Rorsman, Amar Taha, Annica Ravn-Fischer, Arash Hadi Jafari, Shavan Yassinson and Andreas Martinsson contributed significantly to interpretation of data and revision of the manuscript.

All authors unanimously agreed on all aspects of the work, including the accuracy and integrity of the manuscript as well as the results. All authors were also involved in responding to reviewer concerns.

For peer review only

References

1. Goldenberg, I. *et al.* Long-term benefit of primary prevention with an implantable cardioverter-defibrillator: an extended 8-year follow-up study of the Multicenter Automatic Defibrillator Implantation Trial II. *Circulation* **122**, 1265–1271 (2010).
2. Hussein, A. A. & Wilkoff, B. L. Cardiac Implantable Electronic Device Therapy in Heart Failure. *Circ Res* **124**, 1584–1597 (2019).
3. Schmidt, A. *et al.* Infarct Tissue Heterogeneity by Magnetic Resonance Imaging Identifies Enhanced Cardiac Arrhythmia Susceptibility in Patients With Left Ventricular Dysfunction. *Circulation* **115**, 2006–2014 (2007).
4. Tamaki, S. *et al.* Cardiac Iodine-123 Metaiodobenzylguanidine Imaging Predicts Sudden Cardiac Death Independently of Left Ventricular Ejection Fraction in Patients With Chronic Heart Failure and Left Ventricular Systolic Dysfunction. *Journal of the American College of Cardiology* **53**, 426–435 (2009).
5. Yan, A. T. *et al.* Characterization of the Peri-Infarct Zone by Contrast-Enhanced Cardiac Magnetic Resonance Imaging Is a Powerful Predictor of Post–Myocardial Infarction Mortality. *Circulation* **114**, 32–39 (2006).
6. Myerburg, R. J. & Junttila, M. J. Sudden Cardiac Death Caused by Coronary Heart Disease. *Circulation* **125**, 1043–1052 (2012).
7. Wyse D.George *et al.* Life-threatening ventricular arrhythmias due to transient or correctable causes: high risk for death in follow-up. *Journal of the American College of Cardiology* **38**, 1718–1724 (2001).
8. Greifer, N. & Stuart, E. A. Choosing the Causal Estimand for Propensity Score Analysis of Observational Studies. Preprint at http://arxiv.org/abs/2106.10577 (2023).
9. Austin, P. C. An Introduction to Propensity Score Methods for Reducing the Effects of Confounding in Observational Studies. *Multivariate Behavioral Research* **46**, 399–424 (2011).
10. Helle Søholm, Early ICD implantation in cardiac arrest survivors with acute coronary syndrome – predictors of implantation, ICD-therapy and long-term survival. Scandinavian Cardiovascular Journal. 2021, VOL. 55, NO. 4, 205-212.

11. Matilde Winther-Jensen et al. Implantable cardioverter defibrillator and survival after out of hospital cardiac arrest due to acute myocardial infarction in Denmark in the years 2001-2012, a nationwide study, European Heart Journal: Acute Cardiovascular Care 2017, Vol 6(2) 144-154.

12. Connolly SJ, Gent M, Roberts RS, et al. Canadian Implantable Defibrillator Study (CIDS): a randomized trial of the implantable cardioverter defibrillator against amiodarone. Circulation. 2000;101(11):1297–1302.

13. Kuck KH, Cappato R, Siebels J, et al. Randomized comparison of antiarrhythmic drug therapy with implantable defibrillators in patients resuscitated from cardiac arrest: the Cardiac Arrest Study Hamburg (CASH). Circulation. 2000;102(7):748–754.

14. Priori SG, Blomstrom-Lundqvist C, Mazzanti A, et al. 2015 ESC Guidelines for the management of patients with ventricular arrhythmias and the prevention of sudden cardiac death: The Task Force for the Management of Patients with Ventricular Arrhythmias and the Prevention of Sudden Cardiac Death of the European Society of Cardiology (ESC). Endorsed by: Association for European Paediatric and Congenital Cardiology (AEPC). Eur Heart J. 2015;36(41):2793–2867.

15. Zipes DP, Camm AJ, Borggrefe M, et al. ACC/AHA/ESC 2006 guidelines for management of patients with ventricular arrhythmias and the prevention of sudden cardiac death: A report of the American College of Cardiology/American Heart Association Task Force and the European Society of Cardiology Committee for Practice Guidelines (Writing Committee to Develop guidelines for management of patients with ventricular arrhythmias and the prevention of sudden cardiac death) developed in collaboration with the European Heart Rhythm Association and the Heart Rhythm Society. Europace. 2006; 8(9):746–837.

16. Task force members of the European Heart Journal, 2022 ESC Guidelines for the management of patients with ventricular arrhythmias and the prevention of sudden cardiac death, European Heart Journal (2022) 43, 3997-4126.

17. M Jerkeman, P Sultanian et al. Trends in survival after cardiac arrest: a Swedish nationwide study over 30 years. European Heart Journal, Volume 43, Issue 46, 7 December 2022, Pages 4817–4829.

18. Peter C. Austin. An Introduction to Propensity Score Methods for Reducing the Effects of Confounding in Observational Studies. Multivariate Behavioral Research, 46:399–424, 2011.

19. Judea Pearl. The seven tools of causal inference, with reflections on machine learning. Communications of the ACM. March 2019. VOL. 62 NO. 3. DOI:10.1145/3241036.

For peer review only

Table 1. Baseline characteristics (abbreviated) in patients who were discharged after out-of-hospital cardiac arrest, in relation to ICD implantation

Variable	No ICD	ICD	P-value	SMD
	2046	959		
Women – n (%)	435 (21.3)	173 (18.1)	0.046	0.081
Age – mean (SD)	63.6 (13.7)	58.5 (15.4)	<0.001	0.347
**Coexisting conditions prior to OHCA**				
Hypertension	607 (29.7)	292 (30.4)	0.694	0.017
Ischemic heart disease	280 (13.7)	242 (25.2)	<0.001	0.295
Stable or unstable angina	213 (10.4)	168 (17.5)	<0.001	0.206
Heart failure	181 (8.8)	174 (18.1)	<0.001	0.275
Acute myocardial infarction	178 (8.7)	161 (16.8)	<0.001	0.244
Type 2 diabetes	164 (8.0)	114 (11.9)	0.001	0.130
Stroke	79 (3.9)	33 (3.4)	0.643	0.022
Renal failure	73 (3.6)	32 (3.3)	0.830	0.013
**Medications prior to OHCA**			Nan	
RAAS blockers	548 (26.8)	339 (35.3)	<0.001	0.186
Anticoagulants	405 (19.8)	331 (34.5)	<0.001	0.336
Beta-blockers	434 (21.2)	293 (30.6)	<0.001	0.214
Lipid lowering drugs	349 (17.1)	267 (27.8)	<0.001	0.261
**Location of cardiac arrest**			<0.001	0.477
Home	832 (40.7)	411 (42.9)		
Public place	644 (31.5)	447 (46.6)		
Other places	568 (27.8)	101 (10.5)		
**Prehospital interventions**				
Mechanical compressions	514 (26.2)	257 (27.7)	0.416	0.034

Intubation	295 (14.7)	147 (15.6)	0.591	0.023
Defibrillated, any	1738 (85.8)	867 (91.1)	<0.001	0.165
Adrenaline	719 (36.0)	411 (43.8)	<0.001	0.161
Amiodarone	352 (17.7)	246 (26.4)	<0.001	0.211
**Critical time intervals, minutes – median (IQR)**			Nan	
Arrest to CPR start	0.00 [0.00, 2.00]	1.00 [0.00, 4.00]	<0.001	0.180
Arrest to first defibrillation	6.00 [1.00, 12.00]	10.00 [6.00, 13.00]	<0.001	0.330
Arrest to ambulance arrival	9.00 [6.00, 13.00]	9.00 [7.00, 14.00]	0.021	0.095
Arrest to ROSC	11.00 [4.00, 18.00]	13.00 [8.50, 21.00]	<0.001	0.263
**Initial rhythm**			0.001	0.167
VF/pVT	1667 (92.6)	852 (96.2)		
PEA	65 (3.6)	12 (1.4)		
Asystole	69 (3.8)	22 (2.5)		
Circulation on hospital arrival	1932 (95.3)	913 (95.8)	0.614	0.024
Conscious on hospital arrival	1021 (50.9)	345 (36.8)	<0.001	0.288
**Circumstances at time of arrest**			Nan	
Witnessed arrest	1879 (92.7)	866 (91.4)	0.283	0.045
Bystander CPR	1080 (54.4)	743 (78.6)	<0.001	0.531
**Inhospital interventions**			Nan	
PCI	1495 (74.0)	364 (38.9)	<0.001	0.756
CABG	99 (4.9)	34 (3.6)	0.148	0.063

Abbreviations:
ROSC = Return of Spontaneous Circulation, PCI = Percutaneous coronary intervention, CABG = Coronary artery bypass grafting, ECMO = Extracorporeal membrane oxygenation, ICD = Implantable cardioverter defibrillator, VF = Ventricular fibrillation, PVT = Pulseless ventricular tachycardia, EMS = Emergency medical system, PEA = Pulseless electrical activity, AED = Automated external defibrillator, EU = European Union.
SMD = Standardized mean difference, is calculated as the difference in means between the two groups, divided by their combined standard deviation. Balance was defined as SMD <0.1.

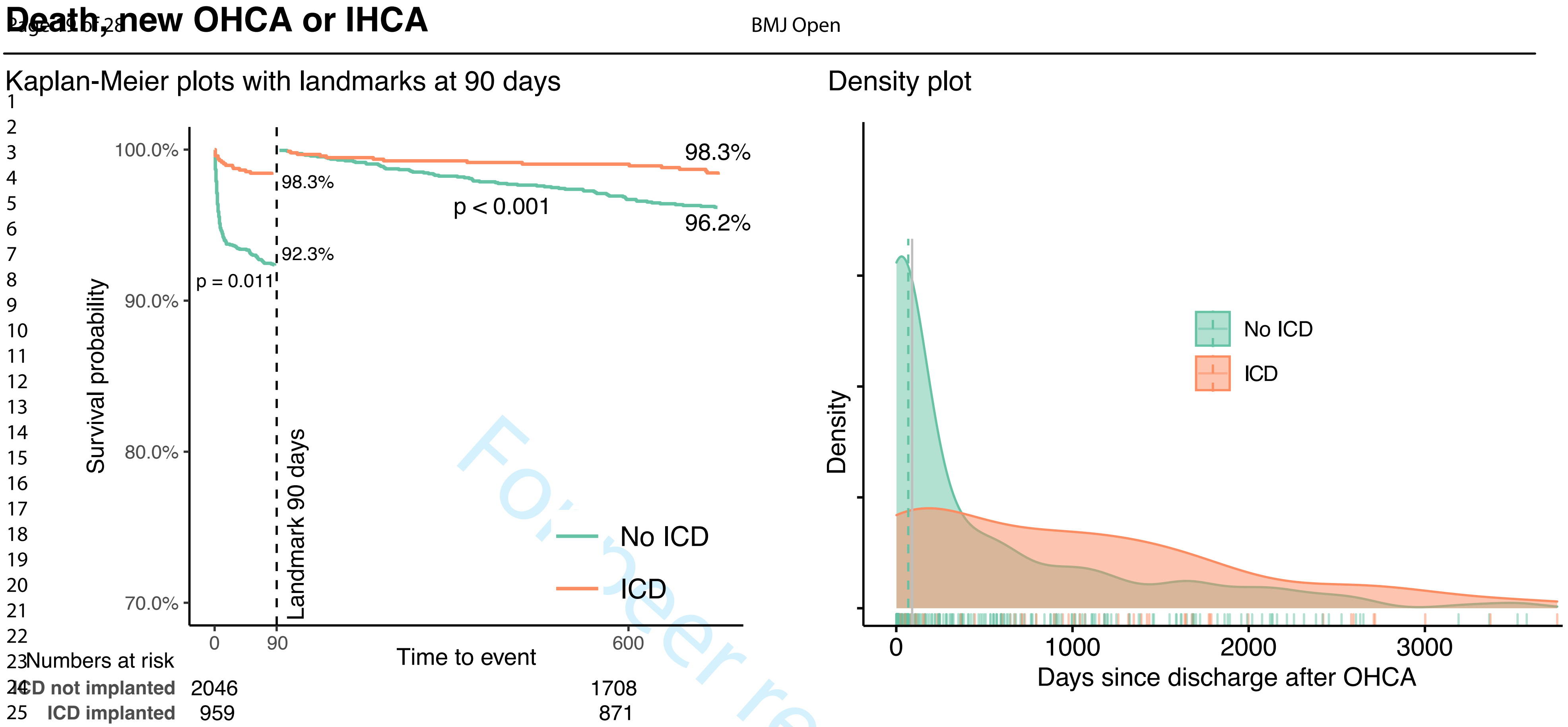
Death, new OHCA or IHCA
Kaplan-Meier plots with landmarks at 90 days
100.0%
90.0%
80.0%
70.0%
Survival probability
98.3%
92.3%
p = 0.011
Landmark 90 days
98.3%
p < 0.001
96.2%
No ICD
ICD
0
90
600
Time to event
Numbers at risk
ICD not implanted 2046 1708
ICD implanted 959 871
Density plot
Density
No ICD
ICD
0
1000
2000
3000
Days since discharge after OHCA

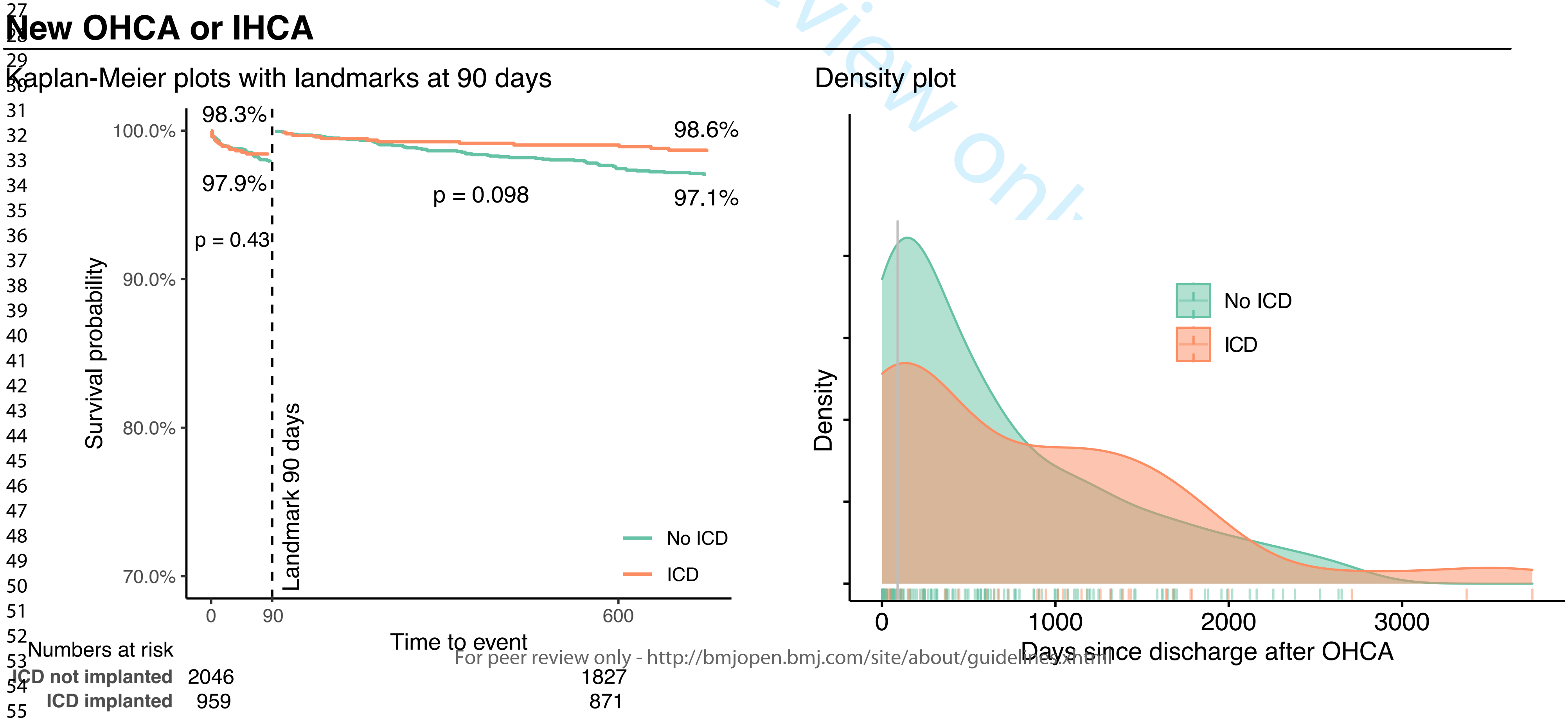
New OHCA or IHCA
Kaplan-Meier plots with landmarks at 90 days
100.0%
90.0%
80.0%
70.0%
Survival probability
98.3%
97.9%
p = 0.43
Landmark 90 days
98.6%
p = 0.098
97.1%
No ICD
ICD
0
90
600
Time to event
Numbers at risk
ICD not implanted 2046 1827
ICD implanted 959 871
Density plot
Density
No ICD
ICD
0
1000
2000
3000
Days since discharge after OHCA

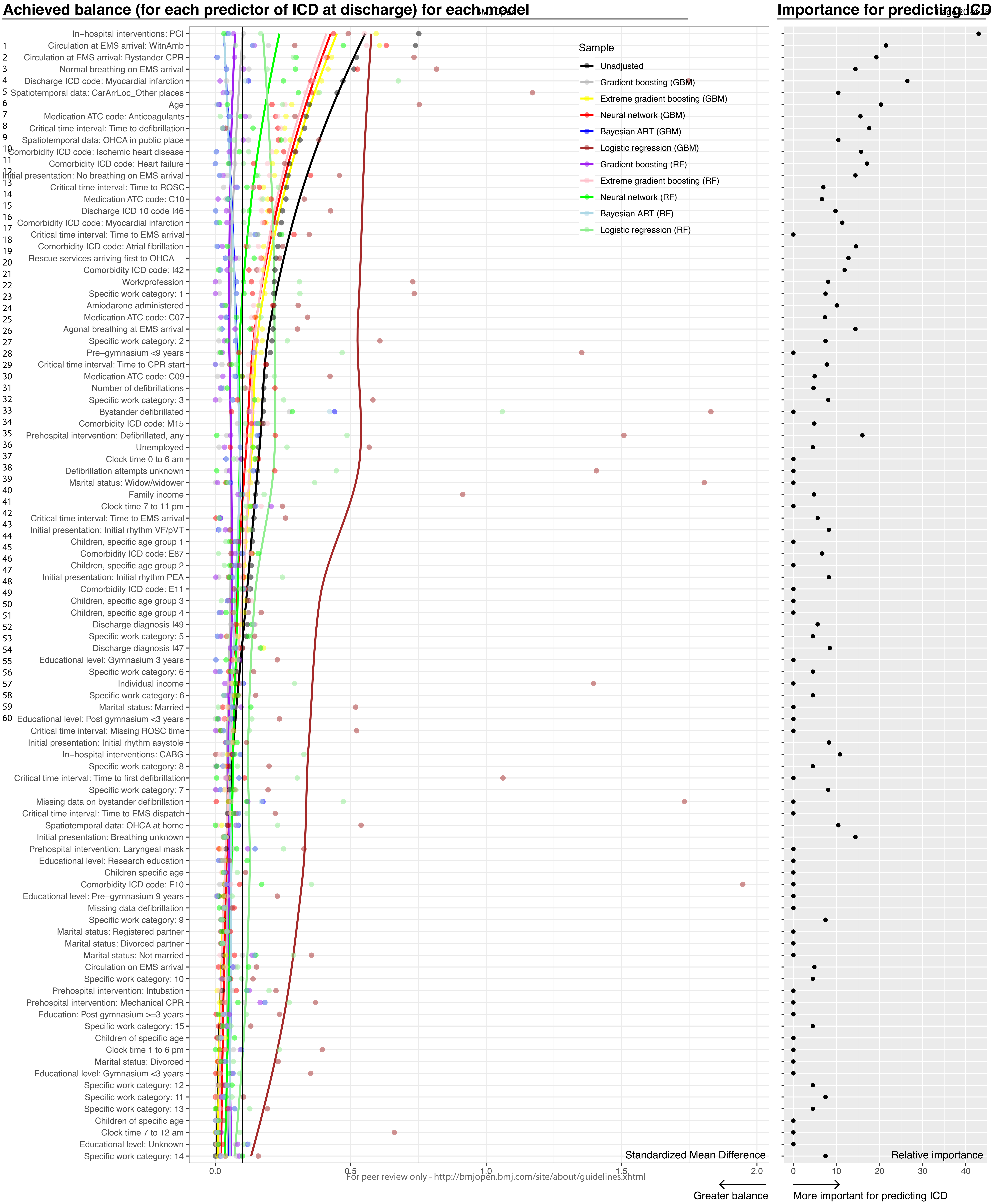
Achieved balance (for each predictor of ICD at discharge) for each model
Importance for predicting ICD
Sample
Unadjusted
Gradient boosting (GBM)
Extreme gradient boosting (GBM)
Neural network (GBM)
Bayesian ART (GBM)
Logistic regression (GBM)
Gradient boosting (RF)
Extreme gradient boosting (RF)
Neural network (RF)
Bayesian ART (RF)
Logistic regression (RF)
In−hospital interventions: PCI
Circulation at EMS arrival: WitnAmb
Circulation at EMS arrival: Bystander CPR
Normal breathing on EMS arrival
Discharge ICD code: Myocardial infarction
Spatiotemporal data: CarArrLoc_Other places
Age
Medication ATC code: Anticoagulants
Critical time interval: Time to defibrillation
Spatiotemporal data: OHCA in public place
Comorbidity ICD code: Ischemic heart disease
Comorbidity ICD code: Heart failure
Initial presentation: No breathing on EMS arrival
Critical time interval: Time to ROSC
Medication ATC code: C10
Discharge ICD 10 code I46
Comorbidity ICD code: Myocardial infarction
Critical time interval: Time to EMS arrival
Comorbidity ICD code: Atrial fibrillation
Rescue services arriving first to OHCA
Comorbidity ICD code: I42
Work/profession
Specific work category: 1
Amiodarone administered
Medication ATC code: C07
Agonal breathing at EMS arrival
Specific work category: 2
Pre−gymnasium <9 years
Critical time interval: Time to CPR start
Medication ATC code: C09
Number of defibrillations
Specific work category: 3
Bystander defibrillated
Comorbidity ICD code: M15
Prehospital intervention: Defibrillated, any
Unemployed
Clock time 0 to 6 am
Defibrillation attempts unknown
Marital status: Widow/widower
Family income
Clock time 7 to 11 pm
Critical time interval: Time to EMS arrival
Initial presentation: Initial rhythm VF/pVT
Children, specific age group 1
Comorbidity ICD code: E87
Children, specific age group 2
Initial presentation: Initial rhythm PEA
Comorbidity ICD code: E11
Children, specific age group 3
Children, specific age group 4
Discharge diagnosis I49
Specific work category: 5
Discharge diagnosis I47
Educational level: Gymnasium 3 years
Specific work category: 6
Individual income
Specific work category: 6
Marital status: Married
Educational level: Post gymnasium <3 years
Critical time interval: Missing ROSC time
Initial presentation: Initial rhythm asystole
In−hospital interventions: CABG
Specific work category: 8
Critical time interval: Time to first defibrillation
Specific work category: 7
Missing data on bystander defibrillation
Critical time interval: Time to EMS dispatch
Spatiotemporal data: OHCA at home
Initial presentation: Breathing unknown
Prehospital intervention: Laryngeal mask
Educational level: Research education
Children specific age
Comorbidity ICD code: F10
Educational level: Pre−gymnasium 9 years
Missing data defibrillation
Specific work category: 9
Marital status: Registered partner
Marital status: Divorced partner
Marital status: Not married
Circulation on EMS arrival
Specific work category: 10
Prehospital intervention: Intubation
Prehospital intervention: Mechanical CPR
Education: Post gymnasium >=3 years
Specific work category: 15
Children of specific age
Clock time 1 to 6 pm
Marital status: Divorced
Educational level: Gymnasium <3 years
Specific work category: 12
Specific work category: 11
Specific work category: 13
Children of specific age
Clock time 7 to 12 am
Educational level: Unknown
Specific work category: 14
Standardized Mean Difference
0.0
0.5
1.0
1.5
2.0
Greater balance
Relative importance
0
10
20
30
40
More important for predicting ICD

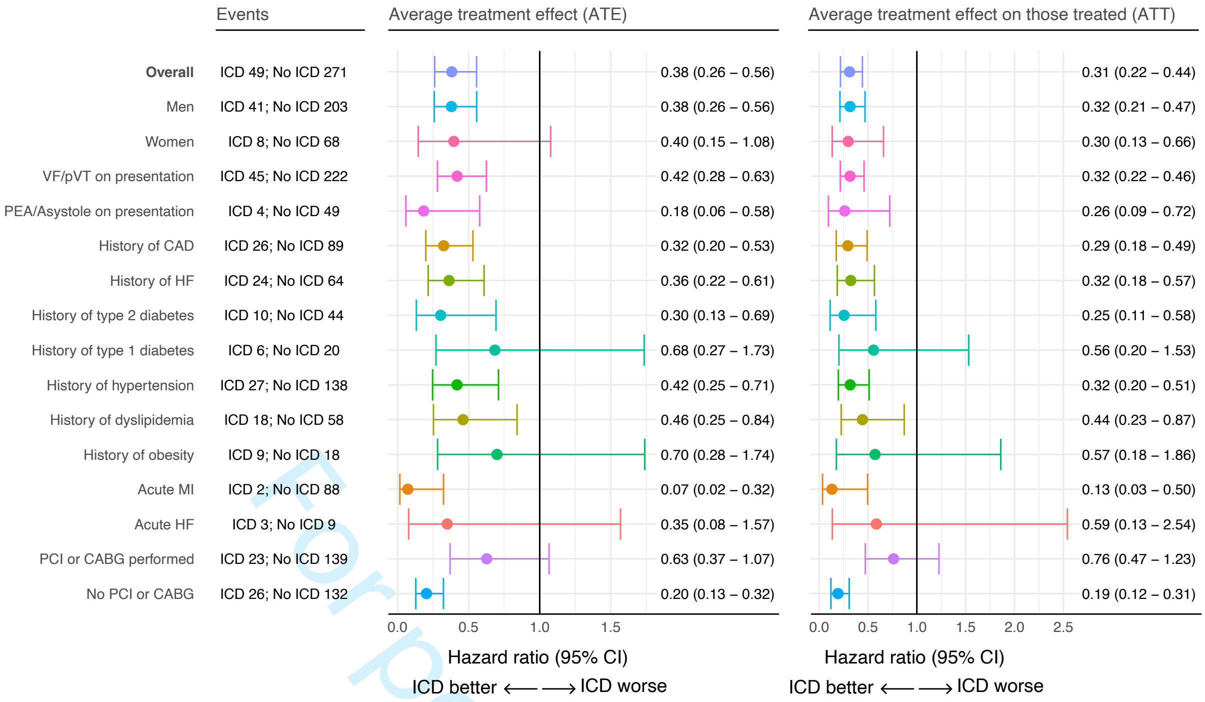

Figure 3. Hazard ratios for death, recurrent OHCA or IHCA, using the final model.

For peer review only

Supplementary Table 1. Baseline characteristics (complete table) in patients who were discharged after out-of-hospital cardiac arrest, in relation to ICD implantation

Variable	No ICD	ICD	P-value	SMD
	2046	959		
Women – n (%)	435 (21.3)	173 (18.1)	0.046	0.081
Age – mean (SD)	63.6 (13.7)	58.5 (15.4)	<0.001	0.347
Born abroad	317 (15.6)	148 (15.5)		
Disposable income, family – median (IQR)	8870.00 [6643.00, 11624.75]	9754.00 [7462.00, 12052.00]	<0.001	0.144
Disposable income, individual – median (IQR)	4454.00 [3062.00, 6171.00]	4951.00 [3353.00, 6296.00]	0.001	0.078
Not employed	978 (52.3)	384 (43.9)		
**Educational level**			0.001	0.203
Pre gymnasium <9 years	344 (18.2)	106 (11.8)		
Post gymnasium 3 years	218 (11.5)	123 (13.7)		
Post gymnasium 3 years or longer	240 (12.7)	124 (13.9)		
Married	1070 (52.7)	540 (56.3)		
**Coexisting conditions prior to OHCA**			Nan	
Hypertension	607 (29.7)	292 (30.4)	0.694	0.017
Ischemic heart disease	280 (13.7)	242 (25.2)	<0.001	0.295
Dyslipidemia	245 (12.0)	171 (17.8)	<0.001	0.165
Stable or unstable angina	213 (10.4)	168 (17.5)	<0.001	0.206
Atrial fibrillation	199 (9.7)	169 (17.6)	<0.001	0.231
Heart failure	181 (8.8)	174 (18.1)	<0.001	0.275
Acute myocardial infarction	178 (8.7)	161 (16.8)	<0.001	0.244
Type 2 diabetes	164 (8.0)	114 (11.9)	0.001	0.130
Alcohol dependency	150 (7.3)	62 (6.5)	0.431	0.034

For peer review only - http://bmjopen.bmj.com/site/about/guidelines.xhtml

Phobic syndromes	125 (6.1)	48 (5.0)	0.260	0.048
Manic episode	114 (5.6)	34 (3.5)	0.021	0.097
Malignant melanoma	93 (4.5)	34 (3.5)	0.241	0.051
Stroke	79 (3.9)	33 (3.4)	0.643	0.022
Transient ischemic attack (TIA)	63 (3.1)	46 (4.8)	0.025	0.088
Renal failure	73 (3.6)	32 (3.3)	0.830	0.013
Aortic stenosis	54 (2.6)	42 (4.4)	0.016	0.095
Type 1 diabetes	60 (2.9)	31 (3.2)	0.739	0.017
**Medications prior to OHCA**			Nan	
RAAS blockers	548 (26.8)	339 (35.3)	<0.001	0.186
Anticoagulants	405 (19.8)	331 (34.5)	<0.001	0.336
Beta-blockers	434 (21.2)	293 (30.6)	<0.001	0.214
Lipid lowering drugs	349 (17.1)	267 (27.8)	<0.001	0.261
Calcium channel blockers	310 (15.2)	105 (10.9)	0.002	0.125
Diuretics	250 (12.2)	129 (13.5)	0.374	0.037
Anti-acidic peptic drugs	209 (10.2)	87 (9.1)	0.360	0.039
**Location of cardiac arrest**			<0.001	0.477
Home	832 (40.7)	411 (42.9)		
Public place	644 (31.5)	447 (46.6)		
Other places	568 (27.8)	101 (10.5)		
**Prehospital interventions**				
Mechanical compressions	514 (26.2)	257 (27.7)	0.416	0.034
Intubation	295 (14.7)	147 (15.6)	0.591	0.023
Laryngeal airway	426 (33.9)	227 (38.7)	0.050	0.100
Defibrillated, any	1738 (85.8)	867 (91.1)	<0.001	0.165

For peer review only - http://bmjopen.bmj.com/site/about/guidelines.xhtml

Defibrillated, times – mean (SD)	2.57 (2.27)	2.99 (2.44)	<0.001	0.179
Adrenaline	719 (36.0)	411 (43.8)	<0.001	0.161
Amiodarone	352 (17.7)	246 (26.4)	<0.001	0.211
**Critical time intervals, minutes – median (IQR)**			Nan	
Arrest to EMS dispatch	2.00 [1.00, 3.00]	2.00 [1.00, 3.00]	0.361	0.065
Arrest to CPR start	0.00 [0.00, 2.00]	1.00 [0.00, 4.00]	<0.001	0.180
Arrest to first defibrillation	6.00 [1.00, 12.00]	10.00 [6.00, 13.00]	<0.001	0.330
Arrest to ambulance arrival	9.00 [6.00, 13.00]	9.00 [7.00, 14.00]	0.021	0.095
Emergency call to ambulance arrival	8.00 [5.00, 13.00]	7.00 [5.00, 11.00]	<0.001	0.245
Arrest to ROSC	11.00 [4.00, 18.00]	13.00 [8.50, 21.00]	<0.001	0.263
**Initial rhythm**			0.001	0.167
VF/pVT	1667 (92.6)	852 (96.2)		
PEA	65 (3.6)	12 (1.4)		
Asystole	69 (3.8)	22 (2.5)		
Circulation on hospital arrival	1932 (95.3)	913 (95.8)	0.614	0.024
Conscious on hospital arrival	1021 (50.9)	345 (36.8)	<0.001	0.288
**Circumstances at time of arrest**			Nan	
Witnessed arrest	1879 (92.7)	866 (91.4)	0.283	0.045
Bystander CPR	1080 (54.4)	743 (78.6)	<0.001	0.531
Telephone CPR	304 (51.3)	233 (57.0)	0.086	0.115
Bystander education			0.643	0.071
Laymen not CPR educated	150 (34.2)	109 (37.3)		
Laymen CPR educated	169 (38.6)	104 (35.6)		

For peer review only - http://bmjopen.bmj.com/site/about/guidelines.xhtml

Health care professional	119 (27.2)	79 (27.1)		
Bystander connected AED	167 (15.0)	93 (16.9)	0.354	0.052
Bystander used AED	128 (76.6)	77 (83.7)	0.239	0.177
Witnessed by ambulance	428 (39.3)	50 (10.0)	<0.001	0.724
Ambulance first on scene	1019 (84.6)	430 (77.2)	<0.001	0.190
**Inhospital interventions**			Nan	
PCI	1495 (74.0)	364 (38.9)	<0.001	0.756
CABG	99 (4.9)	34 (3.6)	0.148	0.063

Abbreviations: ROSC = Return of Spontaneous Circulation, PCI = Percutaneous coronary intervention, CABG = Coronary artery bypass grafting, ECMO = Extracorporeal membrane oxygenation, ICD = Implantable cardioverter defibrillator, VF = Ventricular fibrillation, PVT = Pulseless ventricular tachycardia, EMS = Emergency medical system (ambulance), PEA = Pulseless electrical activity, AED = Automated external defibrillator, EU= European Union. SMD = Standardized mean difference, is calculated as the difference in means between the intervention vs non-intervention group, divided of the SD for the covariate. Balance was considered for SMD < 0.1.

1

For peer review only - http://bmjopen.bmj.com/site/about/guidelines.xhtml

Supplementary Figure 1. Relative importance using Random Forest

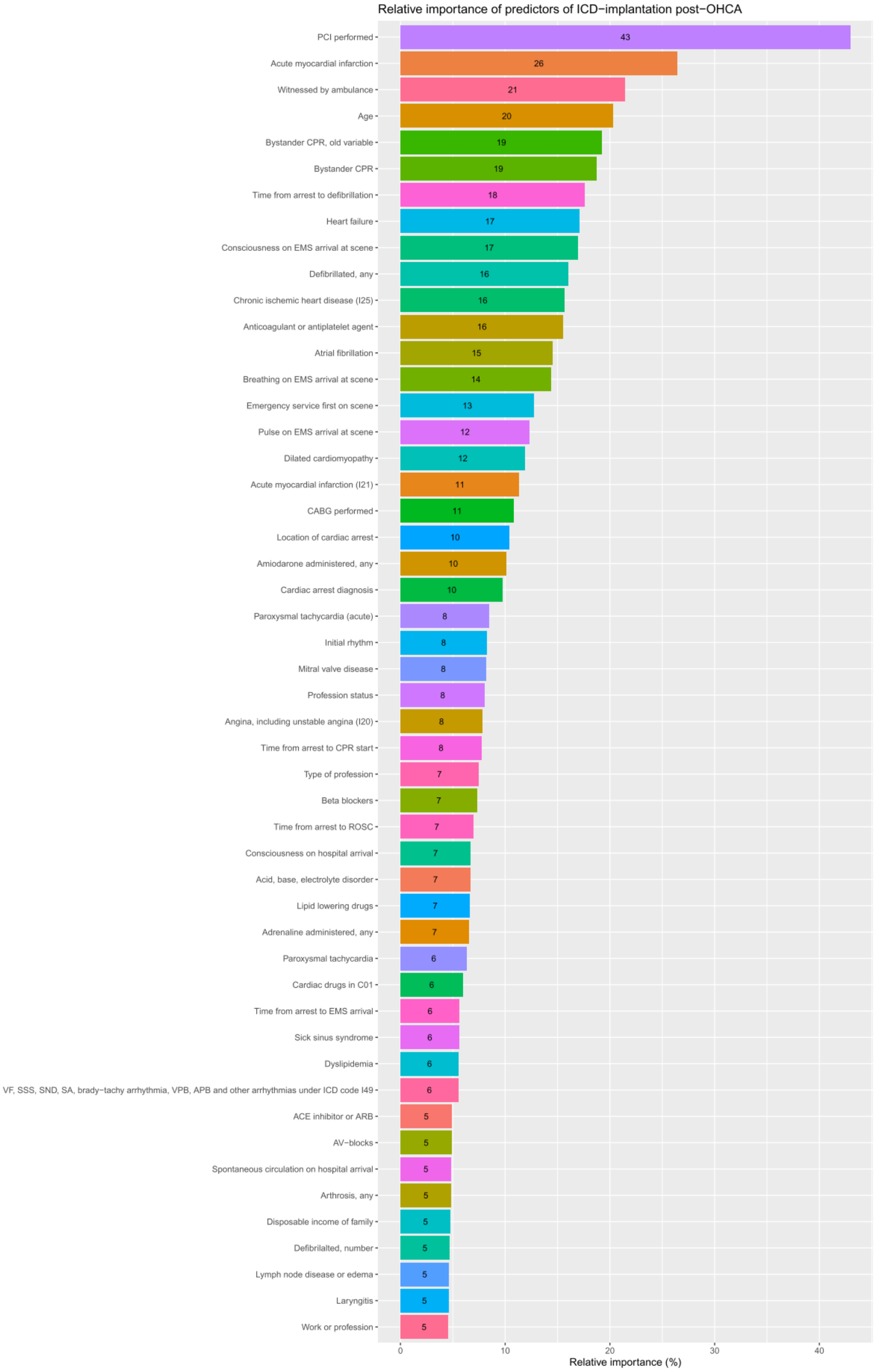

Supplementary Figure 1. Relative importance of top 50 predictors of ICD implantation using random forest. The relative importance is defined as the predictors ability to predict the outcome (i.e. having an ICD at discharge). The direction of the association is not depicted, merely the strength of the association.

Supplementary Figure 2. Relative importance using Gradient Boosting

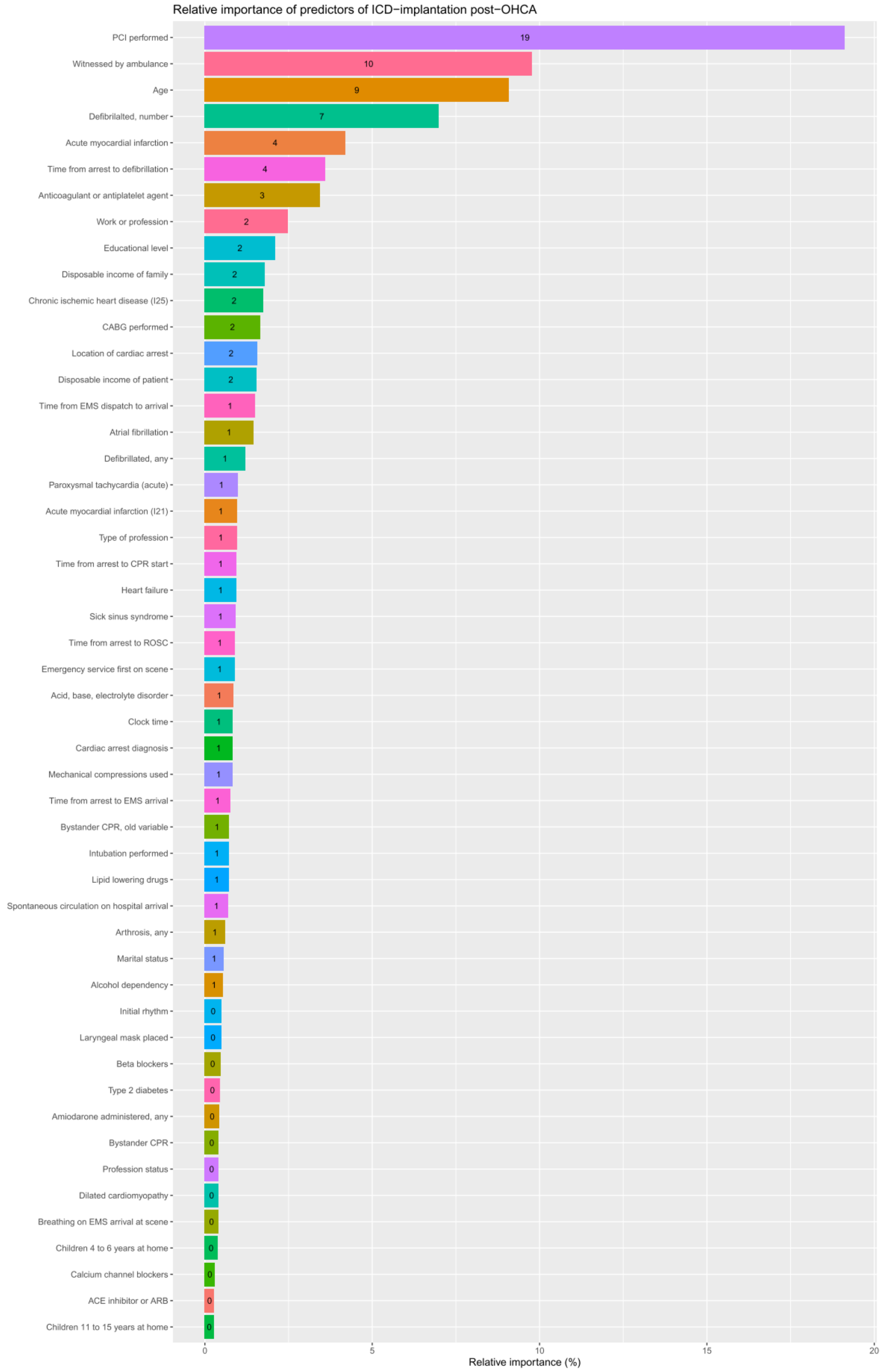

Supplementary Figure 2. Relative importance of top 50 predictors of ICD implantation using gradient boosting. The relative importance is defined as the predictors ability to predict the outcome (i.e. having an ICD at discharge). The direction of the association is not depicted, merely the strength of the association.

Supplementary Figure 3. Cox proportional hazards model for non-balanced covariates

(A)

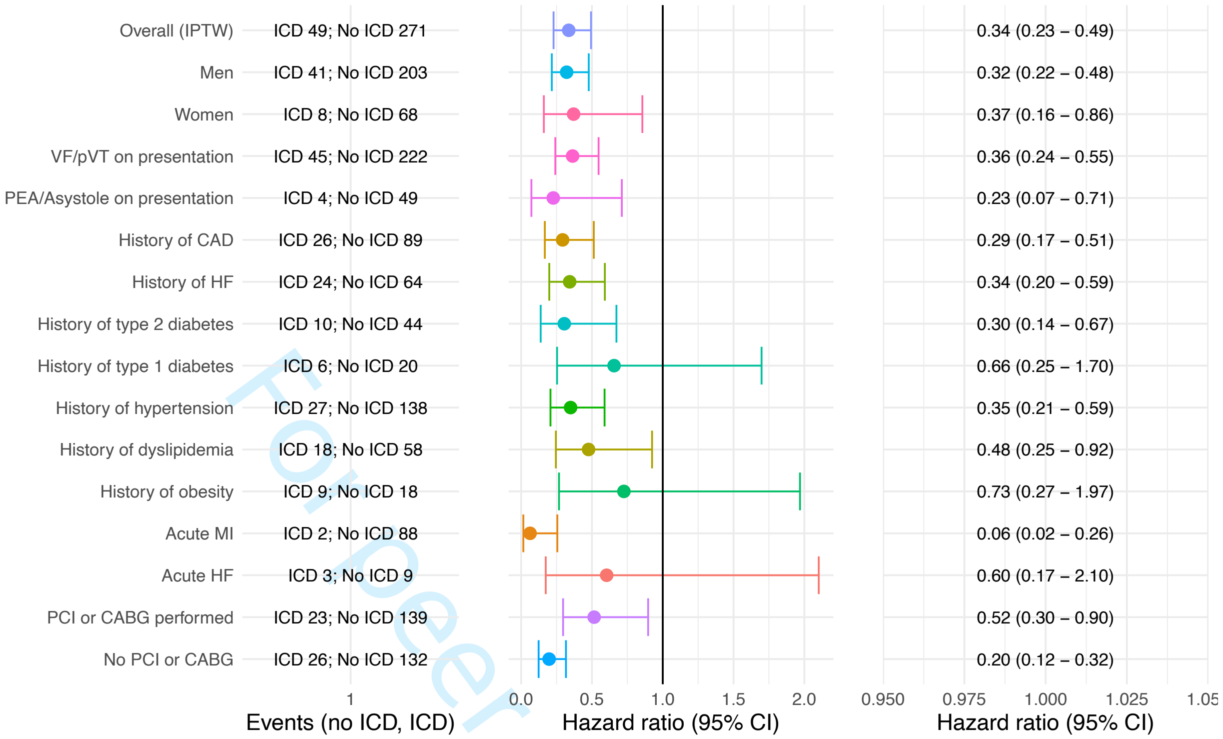

(B)

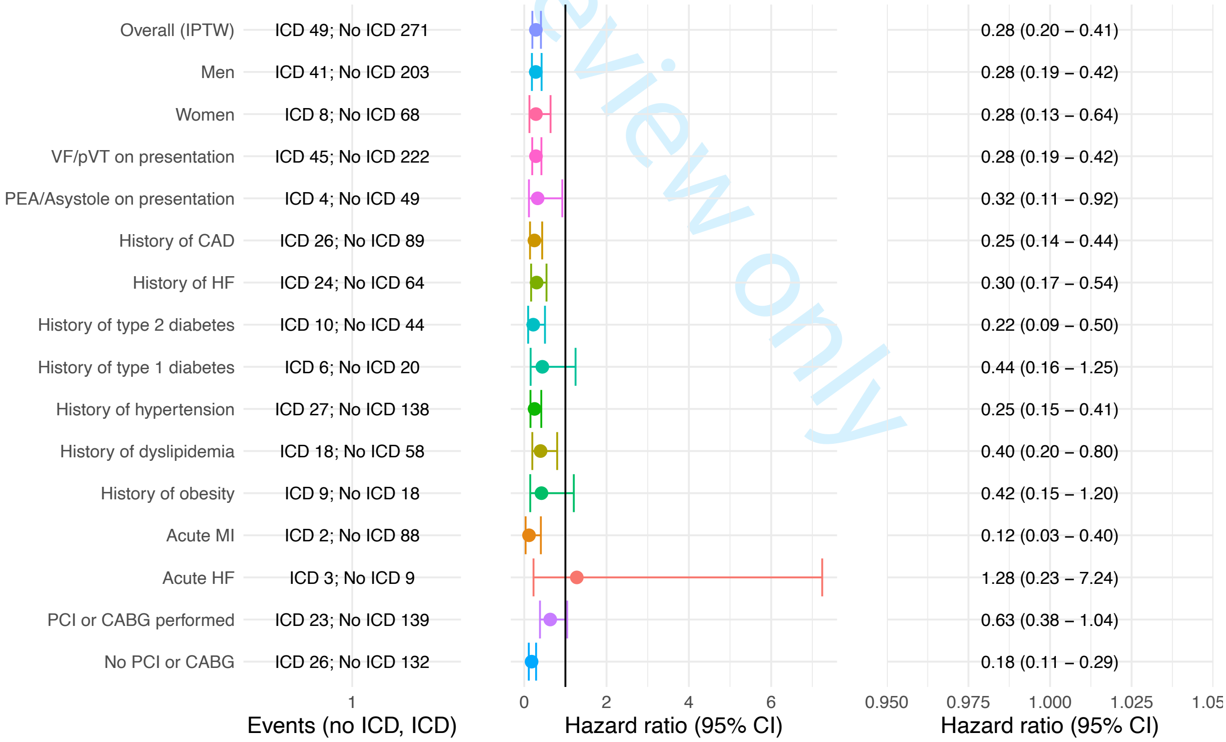

Supplementary figure 3. Cox proportional hazards model for death, recurrent OHCA or IHCA, with adjustments for non-balanced covariates. As evident, adding the non-balanced covariates to the Cox regression did not affect the results. (A) shows results from ATE analysis, and (B) shows results from ATT analysis.

For peer review only

STROBE Statement—checklist of items that should be included in reports of observational studies

	Item No	Recommendation	Page No
**Title and abstract**	1	(*a*) Indicate the study's design with a commonly used term in the title or the abstract	2
		(*b*) Provide in the abstract an informative and balanced summary of what was done and what was found	2
**Introduction**			
Background/rationale	2	Explain the scientific background and rationale for the investigation being reported	4
Objectives	3	State specific objectives, including any prespecified hypotheses	4
**Methods**			
Study design	4	Present key elements of study design early in the paper	5
Setting	5	Describe the setting, locations, and relevant dates, including periods of recruitment, exposure, follow-up, and data collection	5
Participants	6	(*a*) *Cohort study*—Give the eligibility criteria, and the sources and methods of selection of participants. Describe methods of follow-up<br>*Case-control study*—Give the eligibility criteria, and the sources and methods of case ascertainment and control selection. Give the rationale for the choice of cases and controls<br>*Cross-sectional study*—Give the eligibility criteria, and the sources and methods of selection of participants	5
		(*b*) *Cohort study*—For matched studies, give matching criteria and number of exposed and unexposed<br>*Case-control study*—For matched studies, give matching criteria and the number of controls per case	5
Variables	7	Clearly define all outcomes, exposures, predictors, potential confounders, and effect modifiers. Give diagnostic criteria, if applicable	5-7
Data sources/ measurement	8*	For each variable of interest, give sources of data and details of methods of assessment (measurement). Describe comparability of assessment methods if there is more than one group	5-6
Bias	9	Describe any efforts to address potential sources of bias	5-7
Study size	10	Explain how the study size was arrived at	5
Quantitative variables	11	Explain how quantitative variables were handled in the analyses. If applicable, describe which groupings were chosen and why	5-6
Statistical methods	12	(*a*) Describe all statistical methods, including those used to control for confounding	5-7
		(*b*) Describe any methods used to examine subgroups and interactions	5-7
		(*c*) Explain how missing data were addressed	5-7
		(*d*) *Cohort study*—If applicable, explain how loss to follow-up was addressed<br>*Case-control study*—If applicable, explain how matching of cases and controls was addressed<br>*Cross-sectional study*—If applicable, describe analytical methods taking account of sampling strategy	
		(*e*) Describe any sensitivity analyses	

Continued on next page

For peer review only - http://bmjopen.bmj.com/site/about/guidelines.xhtml

**Results**			
Participants	13*	(a) Report numbers of individuals at each stage of study—eg numbers potentially eligible, examined for eligibility, confirmed eligible, included in the study, completing follow-up, and analysed	7-9
		(b) Give reasons for non-participation at each stage	
		(c) Consider use of a flow diagram	
Descriptive data	14*	(a) Give characteristics of study participants (eg demographic, clinical, social) and information on exposures and potential confounders	7-9
		(b) Indicate number of participants with missing data for each variable of interest	
		(c) *Cohort study*—Summarise follow-up time (eg, average and total amount)	
Outcome data	15*	*Cohort study*—Report numbers of outcome events or summary measures over time	8,9
		Case-control study—Report numbers in each exposure category, or summary measures of exposure	
		Cross-sectional study—Report numbers of outcome events or summary measures	
Main results	16	(*a*) Give unadjusted estimates and, if applicable, confounder-adjusted estimates and their precision (eg, 95% confidence interval). Make clear which confounders were adjusted for and why they were included	8,9
		(*b*) Report category boundaries when continuous variables were categorized	
		(*c*) If relevant, consider translating estimates of relative risk into absolute risk for a meaningful time period	
Other analyses	17	Report other analyses done—eg analyses of subgroups and interactions, and sensitivity analyses	9
**Discussion**			
Key results	18	Summarise key results with reference to study objectives	10
Limitations	19	Discuss limitations of the study, taking into account sources of potential bias or imprecision. Discuss both direction and magnitude of any potential bias	11,12
Interpretation	20	Give a cautious overall interpretation of results considering objectives, limitations, multiplicity of analyses, results from similar studies, and other relevant evidence	11,12
Generalisability	21	Discuss the generalisability (external validity) of the study results	10-12
**Other information**			
Funding	22	Give the source of funding and the role of the funders for the present study and, if applicable, for the original study on which the present article is based	12

*Give information separately for cases and controls in case-control studies and, if applicable, for exposed and unexposed groups in cohort and cross-sectional studies.

Note: An Explanation and Elaboration article discusses each checklist item and gives methodological background and published examples of transparent reporting. The STROBE checklist is best used in conjunction with this article (freely available on the Web sites of PLoS Medicine at http://www.plosmedicine.org/, Annals of Internal Medicine at http://www.annals.org/, and Epidemiology at http://www.epidem.com/). Information on the STROBE Initiative is available at www.strobe-statement.org.

For peer review only

For peer review only - http://bmjopen.bmj.com/site/about/guidelines.xhtml
